# Activation of the Ground and Excited State of a Luminescent Osmium(VI) Dioxo Dicyano Complex with Lewis Acids

**DOI:** 10.1021/jacs.6c04322

**Published:** 2026-05-04

**Authors:** Li-Xin Wang, Fang Xu, Ailin Gao, Jing Xiang, Yi Pan, Rui-Yue Qi, Ji-Yan Liu, Tian-Ci Li, Kai-Chung Lau, Tai-Chu Lau

**Affiliations:** † Key Laboratory of Optoelectronic Chemical Materials and Devices (Ministry of Education), School of Optoelectronic Materials and Technology, 74777 Jianghan University, Wuhan 430100, China; ‡ Department of Chemistry, 53025 City University of Hong Kong, Tat Chee Avenue, Kowloon Tong, Hong Kong 999077, China

## Abstract

The activation by Lewis acids (LAs) and visible-light excitation are two appealing strategies for generating highly oxidizing metal oxo species for catalytic oxidation reactions. Herein, we report the first systematic study on the activation of both the ground and excited states of a luminescent osmium­(VI) dioxo dicyano complex (**OsO_2_**). Strong interactions of **OsO_2_** with a variety of LAs were found via binding to the cyano ligands: LA–NC–Os–CN–LA, which allow the isolation and structural determination of the first examples of M=O/LA adducts. **OsO_2_/2LA** interactions also cause a large increase in the reduction potentials/oxidizing power of the adducts in both ground and excited states. Notably, the reduction potential (*E*_pc_) of **OsO_2_** is shifted from −0.04 V (vs. NHE) to 1.21 V in the presence of Sc­(OTf)_3_, which enables it to oxidize a variety of organic substrates. More significantly, the *E*_pc_ for the excited state of **OsO_2_** (OsO_2_*) is shifted from 2.21 to 3.50 V upon binding to Sc­(OTf)_3_, which to our knowledge is the strongest metal oxo oxidant ever generated in solution. OsO_2_*/2Sc­(OTf)_3_ readily catalyzes the oxidation of benzene and its derivatives by H_2_O_2_ at ambient conditions, with a TON up to 8660 and gram-scale production of phenol from benzene.

## Graphical abstract

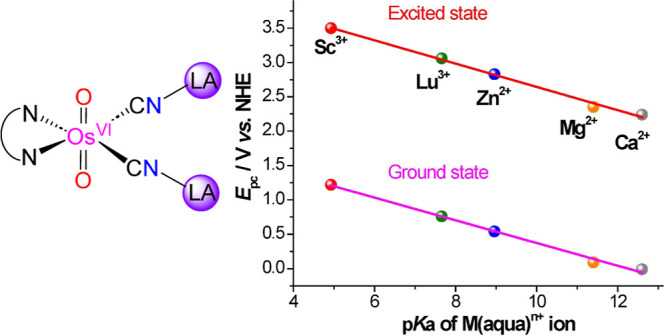

## Introduction

High-valent metal oxo species (M=O) play important roles in numerous biological and chemical oxidation processes.^1–9^ Nature make use of iron oxo species in cytochrome P450 to oxidize a variety of substrates, including unactivated C–H bonds;^10–13^ and (μ-oxo) diiron species in methane monoxygenase to oxidize methane.^14,15^ On the other hand, few stable synthetic metal oxo species have oxidizing power comparable to these enzymes.^16–23^ Highly oxidizing metal oxos generated by chemical oxidation of low valent precursors often decompose easily or are stable only at low temperatures.^24–28^ An appealing strategy to overcome this problem is to design luminescent metal oxo species that possess long-lived, highly oxidizing excited states that can be readily generated by visible-light excitation. Pioneering work by Che and co-workers over three decades ago have shown that *trans*-dioxoosmium­(VI) complexes bearing cyanide and diimine ligands, such as [Os­(O)_2_(4,4′-Me_2_bipy)­(CN)_2_][Bibr ref29] (4,4′-Me_2_bipy = 4,4′-dimethyl-2,2′-bipyridine) and [Os­(O)_2_(dpphen)­(CN)_2_] (**OsO_2_**) (dpphen = 4,7-diphenyl-1,10-phenanthroline),^30^ possess long-lived, strongly oxidizing excited states that can oxidize alkenes and alkanes. In particular, **OsO_2_** is able to undergo aerobic oxidation of cyclohexane.

In the past two decades, there has also been intense interest in the use of Lewis acids to activate metal oxo species. One of us reported in 2006 that the oxidation of cyclohexane by KMnO_4_ is accelerated by over 6 orders of magnitude by adding just a few equivalents of BF_3_, which functions as a Lewis acid that activates MnO_4_^–^ by binding to an oxo ligand.^31^ Subsequently, we and others have reported the use of Lewis acids to activate various other metal oxo species.^32–40^ Another stimulus for this research comes from the discovery of the Mn_4_CaO_5_ active site in the oxygen-evolving center of Photosystem II, where the Ca^2+^ ion in the cluster may function as a Lewis acid.^41–45^ To provide insights into the role of Ca^2+^, Agapie et al. have designed a number of manganese μ-oxo clusters.^46–50^

Extensive work has shown that the oxidizing power of metal oxo species can be enhanced by Lewis acids. It is anticipated that much stronger oxidants may be generated by using Lewis acids to activate the excited states of metal oxos. So far, there is only one report on such a study; the excited state of a Mn­(IV) oxo complex attached to Sc­(OTf)_3_, [(Bn-TPEN)­Mn^IV^(O)]^2+^-(Sc­(OTf)_3_)_2_ (Bn-TPEN = *N*-benzyl-*N*,*N*′,*N*′-tris­(2-pyridylmethyl)-1,2-diamino-ethane), can stoichiometrically oxidize benzene to phenol with 24% yield. The excited state reduction potential of the Mn­(IV)–O is 1.36 V vs. SCE, which is shifted to 2.1 V upon binding to Sc­(OTf)_3_.^51^ However, the detailed interaction of Lewis acids with M=O excited states remains elusive.

Herein, we report the first systemic studies on the activation by a series of Lewis acids (LAs) on both the ground and excited states of an osmium­(VI) dioxo dicyano complex, [Os­(O)_2_(dpphen)­(CN)_2_] (**OsO_2_**), which was reported by Che.^30^ Such a study allows a direct comparison of the ground and excited state interactions with Lewis acids and their reactivity toward various substrates. Notably, in **OsO_2_**, strong binding of LAs via interaction with the cyanide ligands (:N≡C–Os–C≡N: + 2LA → LA-N≡C–Os–C≡N-LA) was found. Such strong binding leads to a large increase in reduction potential and reactivity of the metal oxo; it also enables us to isolate and structurally characterize a number of oxidizing **OsO_2_/2LA** adducts. So far, M=O–LA adducts can only be observed spectroscopically and/or supported by DFT calculations,^34–40^ evidently due in general to the relatively weak binding of M=O to LAs. A number of M-O-LA adducts have been isolated, but the metals are typically in low oxidation states with M-O single bonds (Figure 1).^52–55^ We found that both the ground and excited states are strongly activated by various Lewis acids. Notably, the reduction potential (*E*_pc_) of **OsO_2_** is shifted from −0.04 V (vs. NHE) to 1.21 V in the presence of Sc­(OTf)_3_, which allows it to oxidize a variety of substrates. More significantly, the *E*_pc_ of OsO_2_* is shifted from 2.21 to 3.50 V upon binding to Sc­(OTf)_3_. Such high reduction potential enables OsO_2_*/Sc­(OTf)_3_ to readily oxidize benzene to phenol via an electron-transfer mechanism at ambient conditions; in addition, the oxidation can be made catalytic using H_2_O_2_ as terminal oxidant, reaching a TON of 8660 and gram-scale production of phenol.

**Figure 1:**
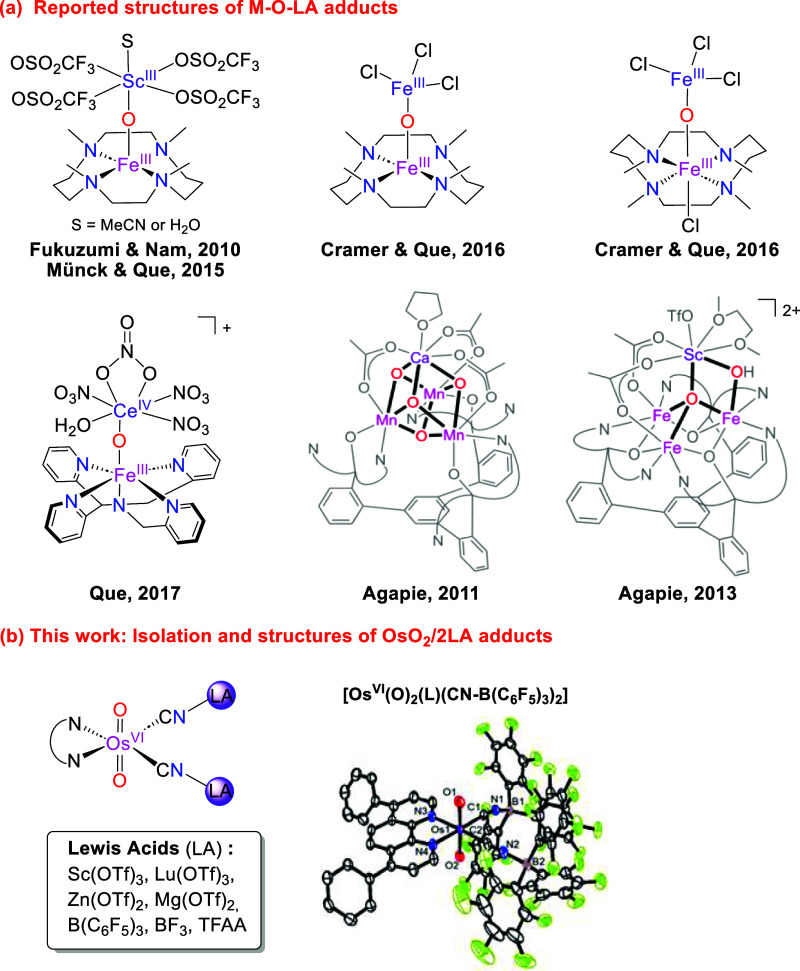
Isolation and structures of M-O/M=O with Lewis acids.

## Results and Discussion

### Cyclic Voltammetry (CV) of OsO_2_ in the Presence of LAs

The CV of **OsO**_**2**_ shows a reversible Os^VI/V^ couple at *E*_1/2_ = −0.68 V (vs Fc^+/0^) in CH_3_CN solution (Figure 2a). The peak current density is linearly dependent on the square root of the scan rate, typical of a diffusion-controlled electron transfer process. Upon the addition of various nonredox active metal salts, the CV became irreversible/quasi-reversible (Figure 2a), with the cathodic peaks (*E*_pc_) shifted to more positive potentials (Table 1), consistent with electron-withdrawing effects of the LAs on **OsO_2_**. The loss of reversibility of the CV upon addition of LAs is attributed to an electrochemical-chemical (EC) process; the OsO_2_/LA species produced are much more oxidizing and unstable, and probably decompose by oxidation of the solvent. A straight line is obtained from the plot of *E*_pc_ versus the p*K*_a_ of M^n+^ aqua ions (Figure 2b), which is a measure of their Lewis acidity. Notably, a positive shift of *E*_pc_ of 1.25 V is found for Sc­(OTf)_3_, the strongest Lewis acid used (Table 1, S1, and Figure S1); such a shift is much larger than those on other metal oxos.^34–40^ Similar *E* vs. p*K*_a_ plots were also reported for the Mn μ-oxo clusters “MMn_3_O_4_” and “MMn_3_O_2_”.^48,50^

**Figure 2:**
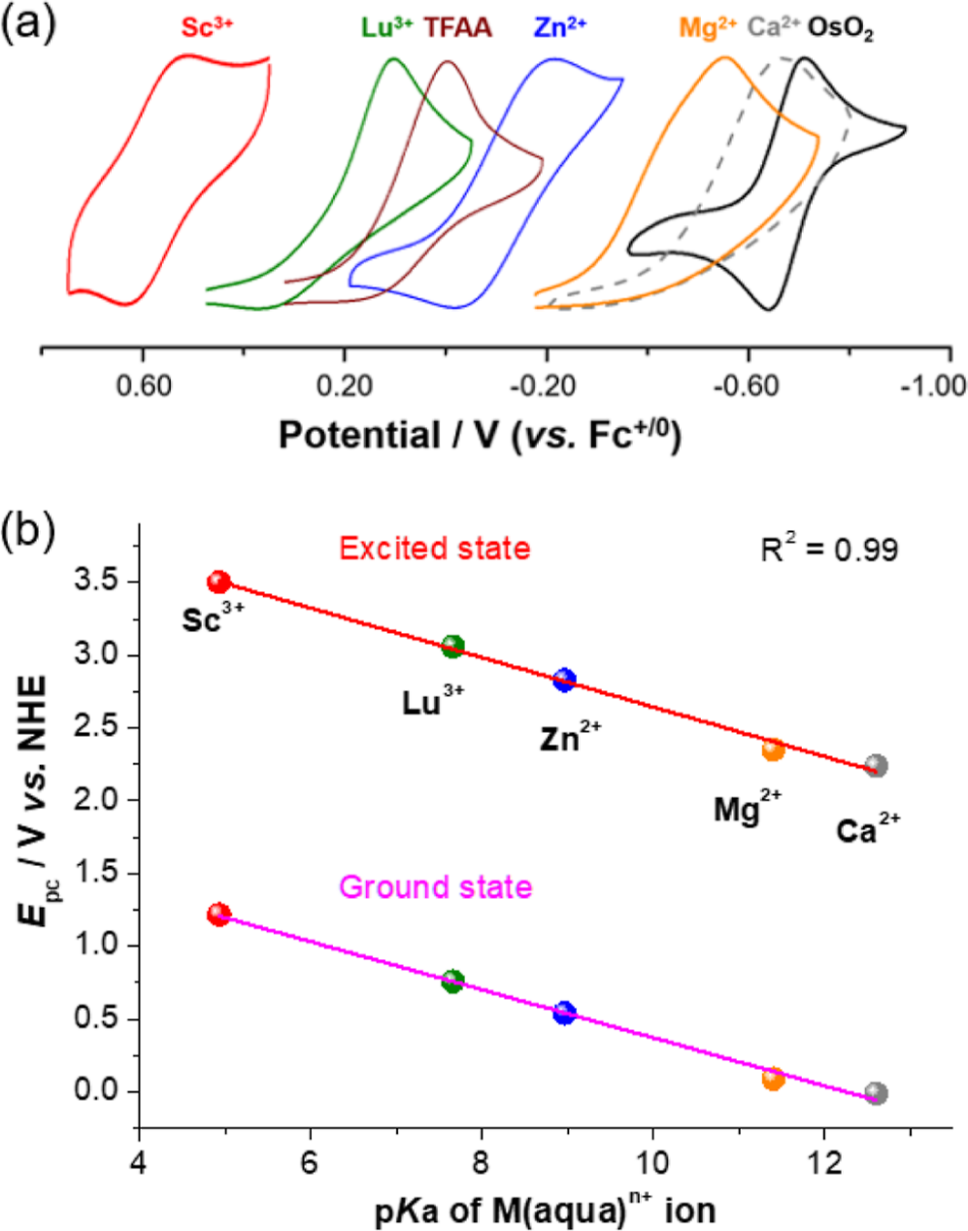
(a) CVs of **OsO_2_** (0.8 mM) in the presence of 10 equiv of LAs (8 mM) in 0.1 M [^n^Bu_4_N]­PF_6_ in CH_3_CN under argon at 23 °C. Scan rate = 0.1 V s^–1^. (b) Plots of *E*_pc_ of the ground and excited state vs p*K*_a_ of the metal aqua ion. Potentials versus Fc^+^/Fc were converted to NHE by adding 0.64 V.

**Table 1:** Ground and Excited State *E*(Os^VI/V^) of **OsO_2_** in the Presence of 10 equiv of LAs

LA	*E*(Os^VI/V^)/V (vs NHE)	*E*_0–0_/eV	*E*(Os^VI*^ ^/V^)/V (vs NHE)
Nil	–0.04	2.25	2.21
Sc(OTf)_3_	1.21	2.29	3.50
Lu(OTf)_3_	0.75	2.31	3.06
Zn(OTf)_2_	0.53	2.30	2.83
Mg(OTf)_2_	0.08	2.27	2.35
Ca(OTf)_2_	–0.02	2.26	2.24
TFAA	0.65	2.26	2.91
B(C_6_F_5_)_3_ ^a^	0.62	2.47	3.09
BF_3_	0.67	2.43	3.10
TFA	0.44	2.26	2.70
HOTf	0.66	2.25	2.91

a 0.2 equiv. was added to **OsO_2_**. *E*_0–0_ estimated from emission spectra at 298 K in degassed MeCN solution.

In addition to the metal salts, other LAs such as trifluoroacetic anhydride (TFAA) (0.65 V), BF_3_ (0.67 V), and B­(C_6_F_5_)_3_ (0.62 V) also cause a positive shift of the *E*_pc_ of Os^VI/V^ (Table 1 and Figure S2). The *E*_pc_ of **OsO_2_** is also influenced by Bronsted acids (BAs) such as CF_3_COOH (TFA) and CF_3_SO_3_H (HOTf), but the effect is less remarkable (*E*_pc_ = 0.44 and 0.66 V in the presence of 10 equiv of TFA and HOTf, respectively).^56^

### Detection and Structures of the OsO_2_/LA Adducts

The CVs described above indicate that there are strong interactions between **OsO_2_** and the LAs. These adducts may be detected by electrospray ionization-mass spectrometry (ESI/MS). The ESI/MS of **OsO_2_** with 5 equiv of Sc­(OTf)_3_ in CF_3_CH_2_OH in the -ve mode exhibits a predominant peak at *m*/*z* 1741, which is assigned to {[Os^VI^(O)_2_(dpphen)­(CN)_2_]·2Sc­(CF_3_SO_3_)_3_·CF_3_SO_3_}^−^ (Figure S3). This suggests the presence of **OsO_2_/2Sc­(OTf)_3_** in solution. The ESI/MS of **OsO_2_** with 5 equiv of Zn­(OTf)_2_ in the -ve mode exhibits a predominant peak at *m*/*z* 1450, assigned to {[Os^VI^(O)_2_(dpphen)­(CN)_2_]·2Zn­(CF_3_SO_3_)_2_·H_2_O·CF_3_CH_2_O}^−^, again suggesting the presence of **OsO_2_/2Zn­(OTf)_2_** in solution (Figure S4).

The **OsO_2_/2LA** adducts could be isolated by treatment of **OsO_2_** with 5-fold excess of LAs in CH_2_Cl_2_. The molecular structure of the following adducts were also determined by X-ray crystallography: [Os^VI^(O)_2_(dpphen)­(CN–B­(C_6_F_5_)_3_)_2_] (**OsO_2_/2B­(C_6_F_5_)_3_**), [Os^VI^(O)_2_(dpphen)­(CN)]­(μ-CN)­[Zn­(Cl)_2_(MeCN)] (**OsO_2_/Zn**), [Os^VI^(O)_2_(dpphen)­(CN)­(CN···TFA)] (**OsO_2_/TFA**) and [Os^VI^(O)_2_(dpphen)­(CN···TFA)_2_] (**OsO_2_/2TFA**) (Figures 1b and 3). **OsO_2_/Zn** was obtained by treatment of **OsO_2_** with 2.5 equiv of ZnCl_2_. **OsO_2_/TFA** and **OsO_2_/2TFA** were prepared by treatment of **OsO_2_** with one and two equiv of TFA, respectively. Selected bond parameters (Å, °) are summarized in Table S2. In all these structures, the LAs and TFA are bonded to **OsO_2_** via the cyanide ligands rather than the oxo ligands. In **OsO_2_**, the two Os–O bond lengths are close to 1.750(3) Å. The corresponding Os–O bond lengths in **OsO_2_/2B­(C_6_F_5_)_3_**, **OsO_2_/Zn**, **OsO_2_/TFA**, and **OsO_2_/2TFA**, are similar to those in **OsO_2_** and are in the range of 1.741(2) to 1.767(2) Å. In addition, these Os–C (1.956–2.056 Å) and C≡N (1.133–1.151 Å) bond lengths in these compounds are also similar to those of **OsO_2_**. In **OsO_2_/TFA**, the TFA forms a strong H-bonding interaction with –CN, as evidenced by a short N···O bond distance of 2.699 Å.

**Figure 3:**
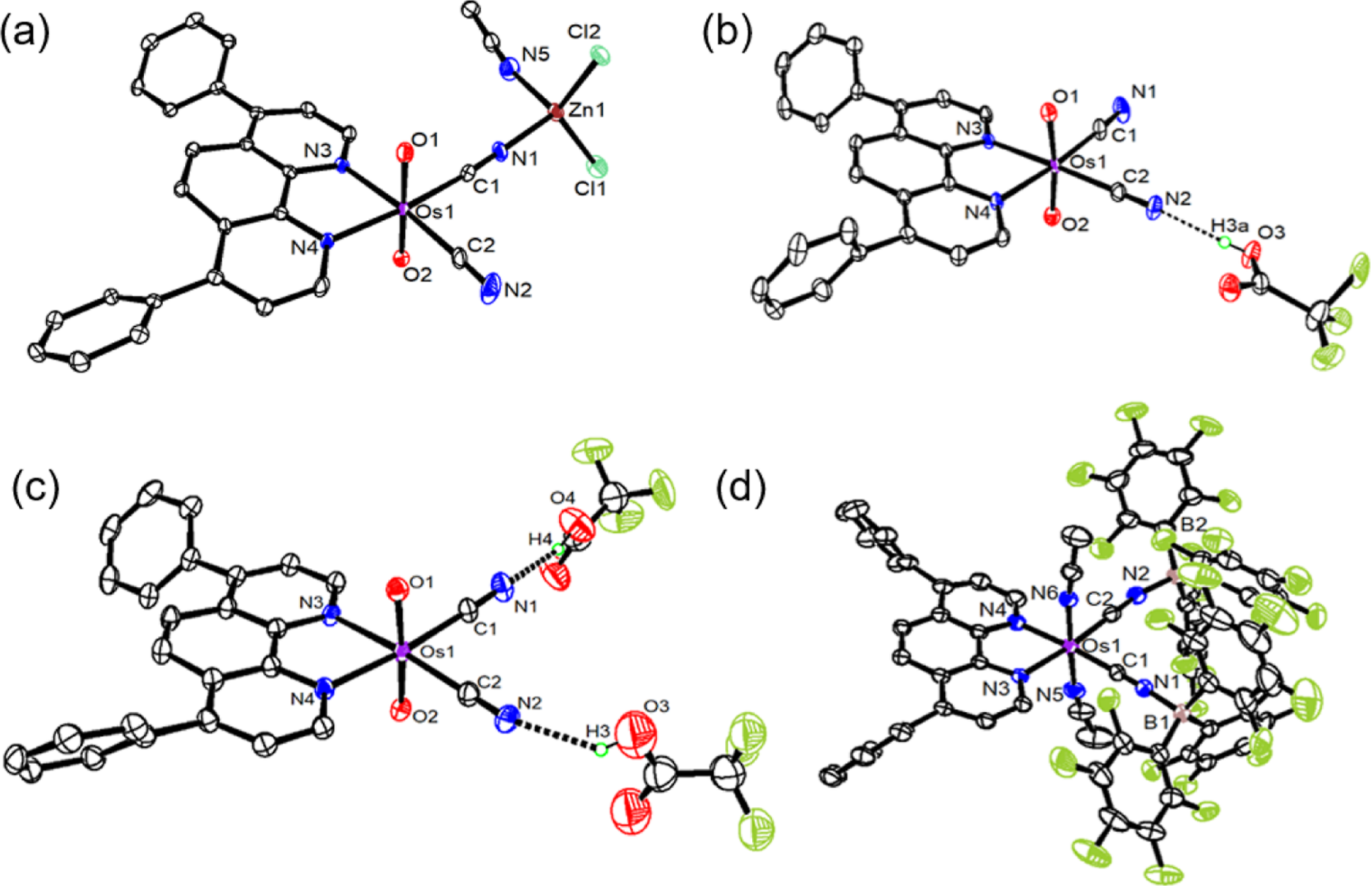
Crystal structures of (a) **OsO_2_/Zn**, (b) **OsO_2_/TFA**, (c) **OsO_2_/2TFA**, and (d) **Os^II^(MeCN)_2_/2B­(C_6_F_5_)_3_**.

The solid adducts were characterized by IR spectroscopy. The IR spectrum of **OsO_2_** shows *v*(C≡N) and *v*(Os=O) stretches at 2158 and 856 cm^–1^, respectively (Figure S6).^30^ In the IR of isolated OsO_2_ + LA/TFA adducts, large shifts in the *v*(C≡N) stretches occur, while there are relatively small shifts in *v*(Os=O) stretches, suggesting that the LAs and TFA bind to the –CN groups rather than to the oxo ligands, which is not unexpected since –CN should be more electron-donating than the oxo ligands. For **OsO_2_/2B­(C_6_F_5_)_3_** and **OsO_2_/Zn**, the *v*(C≡N) stretch occurs at 2260 cm^–1^ and 2216 cm^–1^, and the *v*(Os=O) stretches are found at 856 cm^–1^ and 849 cm^–1^, respectively. Similarly, large shifts of *v*(C≡N) stretches are observed in the IR spectra of other **OsO_2_/2LA** solids, indicating that these LAs mainly interact with the –CN group (Figure S7).

Although there are substantial impacts of LAs on the redox potential and IR of **OsO_2_**, the UV/vis spectrum of **OsO_2_** is much less affected (Figures S8 and S9), which is not unexpected as the absorptions mainly arise from dpphen π–π* transitions.^30^ The ^1^H NMR spectra of these adducts show the proton signals of the dpphen ligand in the normal ranges, in agreement with the diamagnetic properties of the d^2^ electron configuration (Figures S10–S13).

We conclude from the characterizations described above that the adducts formed between **OsO**_**2**_ and excess LAs (∼10 equiv) involve binding of 2LA molecules to the two cyanide ligands, i.e., LA-N≡C–Os­(O)_2_–C≡N-LA. Binding to the cyanide ligands lead to a much larger increase in the reduction potential of **OsO_2_** compared to binding of LAs to the oxo ligands in other metal oxos.

### Photoluminescence Properties of OsO_2_/2LA Adducts

**OsO_2_** exhibits an emission maximum at λ_em_ around 650 nm with an excited state lifetime of τ = 0.95 μs in MeCN at room temperature.^30^ Interestingly, the addition of various metal LAs described above results in little change in the emission maximum (Figures 4 and S14); on the other hand, there is a slight and steady increase in the lifetime and quantum yield with increasing Lewis acidity of the LA (Table 2). For BF_3_ and B­(C_6_F_5_)_3_, the emission maximum changes to 608 and 638 nm, respectively.

**Figure 4:**
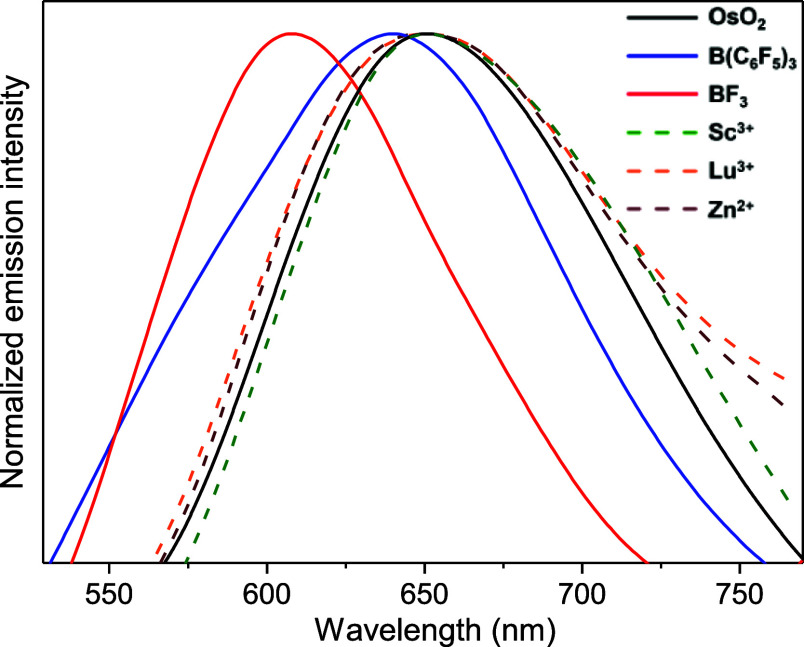
Emission spectra of **OsO_2_** and its adducts with LAs in CH_3_CN at 298 K.

**Table 2:** Photophysical Data for **OsO_2_** in the Presence of 10 equiv of LAs in Degassed MeCN at 298 K

LAs	Emission (λ_max_/nm)^b^	τ_0_/μs	ϕ_em_ (×10^–4^)^c^
Nil	650	0.95	5.0
Sc(OTf)_3_	650	1.18	14.0
Lu(OTf)_3_	650	1.13	11.5
Zn(OTf)_2_	650	1.02	8.9
Mg(OTf)_2_	650	1.06	6.3
Ca(OTf)_2_	650	1.01	5.7
TFAA	650	0.97	5.2
B(C_6_F_5_)_3_ ^a^	638	1.50	9.0
BF_3_	608	1.21	11.5
TFA	650	1.60	4.4
HOTf	650	1.54	4.7

a 2 equiv. added.

b Excited at 380 nm.

c Measured by the relative method using a 0.1 M H_2_SO_4_ solution of quinine sulfate as a standard.

DFT calculations showed that the emission of **OsO_2_** arises from the LMCT state [*p*_π_(O^2–^) → *d*_π*_(Os^VI^)], where *d*_π*_ = *d*_*xz*_, *d*_*yz*_, as proposed by Che and co-workers.^29^ On the other hand, the emissions of **OsO_2_/BF_3_** and **OsO_2_/Sc­(OTf)_3_** are found to arise from LMCT [π­(phen) → *d*_*yz*_(Os^VI^)] state, which should not be significantly affected by LA coordination to –CN group.

The *E*_0–0_ value of **OsO_2_**, estimated from the emission spectrum is 2.25 eV and the *E* ^0^(Os^VI/V^) obtained by cyclic voltammetry in MeCN is −0.04 V vs NHE. Using the equation: *E* ^0^(Os^VI*/V^) = *E* ^0^ (Os^VI/V^) + *E*_0–0_, the excited state *E* ^0^(Os^VI*/V^) = 2.21 V vs NHE. Using the same equation, the excited state *E*_pc_ for the various OsO_2_ + LA adducts are estimated and shown in Table 1 (see detailed calculation method in the SI).

Notably, *E*_pc_ with the strongest LA, **OsO_2_/2Sc­(OTf)_3_**, is 3.50 V, which is among the strongest metal oxo oxidants generated in solution. An Fe­(V) oxo complex with *E*_pc_ = 3.8 V vs SCE, generated in situ via photo-oxidation of its Fe­(IV) precursor, was also recently reported.^57^ **OsO_2_/2Sc­(OTf)_3_** readily oxidizes benzene (oxidation potential *E* = 2.50 V vs NHE) via an electron transfer process, as described later.

### The Reactivity of OsO_2_/2Sc­(OTf)_3_ in the Ground State

**OsO_2_** in the ground state is highly stable and does not exhibit any oxidizing properties toward common organic and inorganic substrates, as expected from its rather low reduction potential (*E* ^0^ = −0.04 V vs NHE). However, upon binding to LAs via its CN^–^ ligands, the potential is shifted to more positive values, as shown in Table 1. Notably, **OsO_2_/2Sc­(OTf)_3_** with an *E*_pc_ of +1.21 V vs. NHE is a strong oxidant, and it readily oxidizes a variety of substrates via various pathways. It undergoes a one-electron transfer reaction with ferrocene, an O-atom transfer reaction with PPh_3_, and reaction with hydroquinone to benzoquinone via a H-atom abstraction mechanism. It also undergoes C–H bond activation with hydrocarbons (Figure 5). In the reaction with all these substrates, **OsO**_**2**_ with 10 equiv of Sc­(OTf)_3_ was used; under this condition, the predominant species is **OsO_2_/2Sc­(OTf)_3_**. With all substrates investigated, **OsO_2_/2Sc­(OTf)_3_** functions as a four-electron oxidant; it is reduced by substrates in two consecutive steps: Os^VI^O_2_/2Sc­(OTf)_3_ + 2H^+^ + 2e^–^ → Os^IV^O/2Sc­(OTf)_3_ + H_2_O; Os^IV^O/2Sc­(OTf)_3_ + 2H^+^ + 2e^–^ → Os^II^/2Sc­(OTf)_3_ + H_2_O. The Os­(II) species [Os^II^(MeCN)_2_(dpphen)­(CN)_2_{Sc^III^(CF_3_SO_3_)_3_}_2_] could be isolated. Inductively coupled plasma atomic emission spectroscopy (ICP-AES) indicates that the mole ratio of Os to Sc is 1:2, and the IR spectrum shows the *v*(C≡N) stretch at 2080 cm^–1^. The molecular structure of the Os^II^ complex has also been determined by X-ray crystallography (Figure 3d).

**Figure 5:**
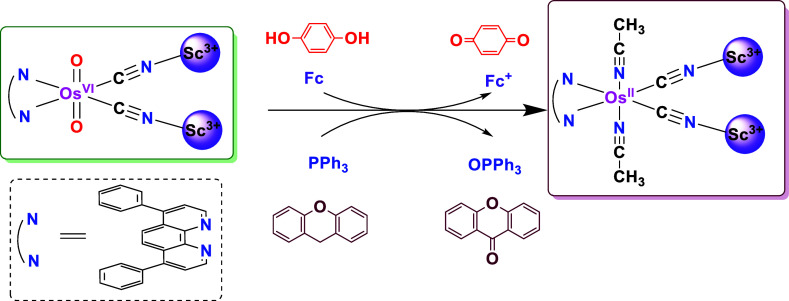
Reactivity of **OsO_2_/2Sc­(OTf)_3_** in the ground state.

For all substrates, the second step is much faster than the first step, so only rates correspond to that of the first step were observed, with the rate law: Rate = *k*_2_[**OsO_2_/2Sc­(OTf)_3_**]­[substrate].

**OsO_2_/2Sc­(OTf)_3_** rapidly oxidizes ferrocene to give 4 equiv of ferrocenium (λ_max_ = 620 nm, ε = 500 M^–1^·s^–1^).^53^ ESI-MS (+ ve mode) shows a ferrocenium peak at *m*/*z* = 186. Kinetics were carried out using bromoferrocene, which is oxidized at a slower and more measurable rate, and *k*_2_ was found to be (70.1 ± 1.6) M^–1^·s^–1^ at 298 K (Figure S15).

**OsO_2_/2Sc­(OTf)_3_** readily undergoes O-atom transfer with PPh_3_ to give 2 equiv of O=PPh_3_, with *k*_2_ = (55.6 ± 0.9) M^–1^·s^–1^ at 298 K (Figures S16 and S17).

Reaction of **OsO_2_/2Sc­(OTf)_3_** with hydroquinone (H_2_Q) produces 2 equiv of 1,4-benzoquinone (Q), with *k*_2_ = (0.53 ± 0.02) M^–1^·s^–1^ at 298 K. The kinetic isotope effect (KIE) was determined by comparing reaction rates in 18:1 MeCN/H_2_O (*k*_H_) and MeCN/D_2_O (*k*_D_). *k*_H_/*k*_D_ was found to be 2.7 ± 0.1, consistent with a H-atom abstraction mechanism (Figures S18 and S19).

### Reactivity of OsO_2_/2Sc­(OTf)_3_ with Hydrocarbons

**OsO_2_/2Sc­(OTf)_3_** readily oxidizes hydrocarbons with C–H bond dissociation energy (BDE) < 80 kcal/mol, such as 10-methyl-9,10-dihydroacridine (AcrH_2_, *k*_2_ = (43.4 ± 1.6) M^–1^·s^–1^) (Figure 6a,b), xanthene (XAN, *k*_2_ = (2.8 ± 0.1) × 10^–2^ M^–1^·s^–1^) (Figure S20a), 9,10-dihydroanthracene (DHA, *k*_2_ = (1.2 ± 0.1) × 10^–3^ M^–1^·s^–1^) (Figure S21), with the formation of [Os^II^(MeCN)_2_(dpphen)­(CN)_2_ (Sc^III^)_2_(CF_3_SO_3_)_6_]. GC and GC/MS revealed that AcrH_2_ and XAN were oxidized to the corresponding ketone products in 87% and 85% yields, respectively; while DHA was converted to anthracene in 84% yield (yields based on **OsO_2_**).

**Figure 6:**
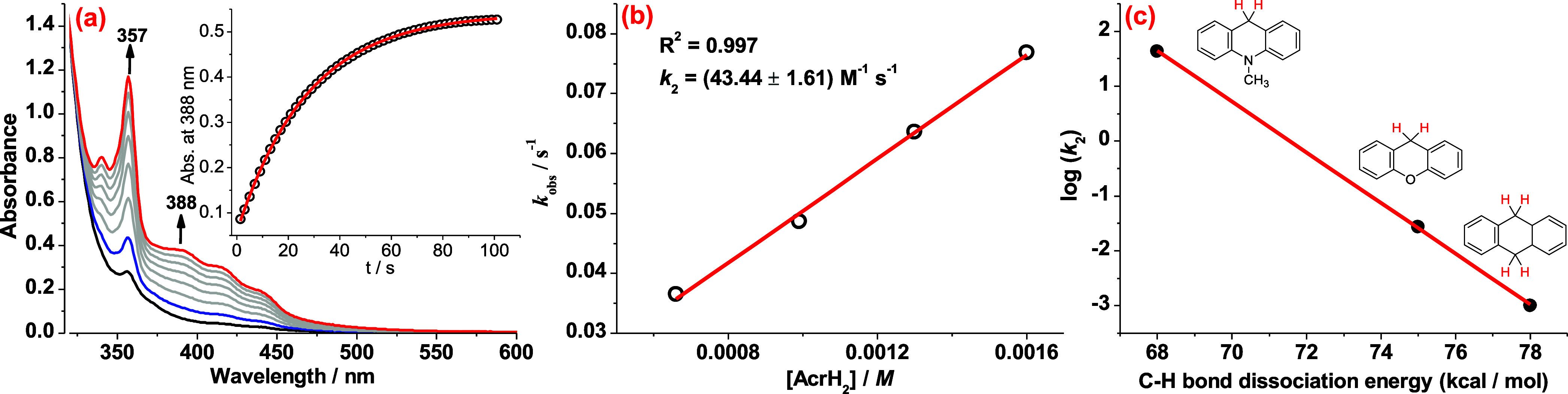
(a) UV–vis spectral changes for the reaction of **OsO_2_** (3.29 × 10^–5^ M) with 10-methyl-9,10-dihydroacridine (3.29 × 10^–4^ M) in the presence of Sc­(OTf)_3_ (3.29 × 10^–4^ M). The inset shows the time course monitored at 388 nm. (b) Plot of *k*_obs_ versus [ 10-methyl-9,10-dihydroacridine]. (c) Plot of log­(*k*_2_) versus substrate C–H BDE.

The plot of log*k*_2_ versus C–H BDE shows a good linear correlation, supporting a rate-limiting hydrogen-atom abstraction (HAT) step (Figure 6c).

The KIE for the reaction of **OsO_2_/2Sc­(OTf)_3_** with XAN (*k*_H_/*k*_D_) was determined to be 8.7 ± 0.1, further supporting a HAT mechanism (Figure S20b).

### Reactivity of OsO_2_/2Sc­(OTf)_3_ in the Excited State

Che and co-workers reported that when **OsO_2_** is excited with UV/vis light, the excited state (OsO_2_*) can undergo stoichiometric and aerobic oxidation of various alkanes, including *n*-hexane and cyclohexane.^30^ In the oxidation of cyclohexane under argon, 4% cyclohexanol and 35% cyclohexanone were produced (based on **OsO_2_**) after 12 h, but 227% of cyclohexanol and 225% of cyclohexanone were found under 1 atm. air, giving a TON of 6.7. TON is based on **OsO_2_** functioning as a two-electron oxidant. The wavelengths of the light used were not specified, however, and in our hands, we obtained a TON of 4.6 after irradiation with light λ > 400 nm for 10 h under air.

We then investigated the oxidation of cyclohexane by OsO_2_*/2Sc­(OTf)_3_. Upon irradiation by visible light (λ > 400 nm) under argon, 7% cyclohexanol and 50% cyclohexanone were produced using 500 equiv of cyclohexane. However, when the experiment was carried out in the presence of air, no increase in yield was observed, which is not unexpected, since OsO_2_*/2Sc­(OTf)_3_ has a much higher reduction potential than OsO_2_*, so air should not be able to reoxidize the reduced Os product. The oxidation, however, can be made catalytic by using H_2_O_2_ as the terminal oxidant. In the presence of 0.8 mM **OsO_2_** and 100 equiv of H_2_O_2_ (25% aqueous), 12.4 mM of cyclohexanol and 21.2 mM of cyclohexanone were produced after irradiation for 10 h, giving a TON of 68 (based on OsO_2_*/2Sc­(OTf)_**3**_ functioning as a two-electron oxidant), and 68% yield based on H_2_O_2_ (Table S3). In contrast, OsO_2_* without Sc­(OTf)_3_ gave a TON of only 7.8.

Apart from cyclohexane, OsO_2_*/2Sc­(OTf)_3_ (*E*_pc_ = 3.50 V vs. NHE) also readily oxidizes benzene (*E* = ∼2.50 V). Stoichiometric oxidation of benzene by OsO_2_*/2Sc­(OTf)_3_ gave phenol exclusively in 66% yield (based on **OsO_2_** used). On the other hand, OsO_2_* alone does not oxidize benzene (Table S4), evidently due to its lower reduction potential (*E* = ∼2.21 V vs NHE). Various other OsO_2_*/2LA are also able to oxidize benzene (e.g., LA = BF_3_, *E*_pc_ = 3.10 V; Lu­(OTf)_3_, *E*_pc_ = 3.06 V; TFAA, *E*_pc_ = 2.91 V; Zn­(OTf)_2_, *E*_1/2_ = 2.83 V) (Table 1 and S5). The oxidation of benzene to phenol by OsO_2_*/2Sc­(OTf)_3_ can again be made catalytic by using H_2_O_2_. Analysis by GC/FID indicated the presence of phenol as the only product, with a yield of 71% based on H_2_O_2_ (100 equiv. used) and a TON of 71 based on **OsO_2_/2Sc­(OTf)**_**3**_ (Table S4). The KIE for the photocatalytic oxidation of benzene was determined to be 1.1 ± 0.1 by the competitive oxidation of C_6_H_6_ and C_6_D_6_ by OsO_2_*/2Sc­(OTf)_3_ (Figure S22), which is consistent with an electron-transfer mechanism.

Competitive catalytic oxidation of an equimolar mixture of cyclohexane and benzene with OsO_2_*/2Sc­(OTf)_3_ and H_2_O_2_ was carried out. The predominant product was phenol (95.1%), while the products from cyclohexane oxidation were only 4.3% (Table S6). Oxidation of benzene most likely occurs by initial one-electron transfer, while that of cyclohexane should occur by HAT. The much lower reactivity toward cyclohexane suggests that the barrier for HAT is higher than that for electron transfer.

In the reaction of OsO_2_*/2Sc­(OTf)_3_ with benzene in MeCN, a green Os^IV^ complex, [Os^IV^(O)­(dpphen)­(MeCN)­(CN)_2_(Sc^III^)_2_(CF_3_SO_3_)_6_] (**Os^IV^O/2Sc­(OTf)_3_**), could be isolated (Figures S23–S25). **Os^IV^O/2Sc­(OTf)_3_** is paramagnetic with μ_eff_ = 1.8 μ_B_ (Evans’ method), together with the broad signals in its ^1^H NMR spectrum, are consistent with a triplet d^4^ Os­(IV) species. On prolonged reaction of OsO_2_*/2Sc­(OTf)_3_ with benzene, **Os^IV^O/2Sc­(OTf)_3_** was gradually reduced to yield the Os^II^ complex, [Os^II^(MeCN)_2_(dpphen)­(CN)_2_(Sc^III^)_2_(CF_3_SO_3_)_6_] (**Os^II^(MeCN)_2_/2Sc­(OTf)_3_**) (Figures S26 and S27).

When **Os^IV^O/2Sc­(OTf)_3_** was treated with H_2_O_2_, it was rapidly oxidized back to **OsO_2_/2Sc­(OTf)_3_**, as monitored by UV/vis spectroscopy and ESI/MS (Figure S28). On the other hand, oxidation of **Os^II^(MeCN)_2_/2Sc­(OTf)_3_** by H_2_O_2_ occurs much more slowly, which is anticipated, since Os^II^ is a good π-donor which is readily stabilized by the strong π-acceptor ligands dpphen and CN^–^ (Figure S29a). The CV of the Os^II^ species shows an oxidation potential at *E*_1/2_ = 1.56 V (vs. Ag/AgCl) (Figure S29b), while the oxidation potential for the Os^IV^ species occurs at *E*_1/2_ = 0.81 V (vs. Ag/AgCl). These results indicate that catalytic oxidation of benzene by H_2_O_2_ stops when [H_2_O_2_] is low and the inactive **Os^II^(MeCN)_2_/2Sc­(OTf)_3_** is gradually produced. On the other hand, as long as [H_2_O_2_] is high, **Os^IV^O/2Sc­(OTf)**_**3**_ that is initially formed will be rapidly oxidized back to **OsO_2_/2Sc­(OTf)_3_**, and catalysis will be sustained. Indeed, the use of a higher concentration of H_2_O_2_ ([**OsO_2_**] = 0.01 mM; [H_2_O_2_] = 0.08 M) resulted in phenol with a higher TON of 2620 after 24 h, with <0.2% benzoquinone detected. Further oxidation of phenol was not observed, most probably due its binding to Sc­(OTf)_3_, which would increase its oxidation potential. The TON was further increased to 8660 after 48 h by adding another portion of H_2_O_2_ (0.08 M), together with the addition of 3 Å molecular sieves to adsorb the water present in 25% H_2_O_2_ (Table S7). By using an even larger amount of catalyst/substrate/oxidant ([**OsO_2_**] = 1 mM, [Sc­(OTf)_3_] = 50 mM, benzene 5 mL, [H_2_O_2_] = 9 M in trifluoroethanol (8 mL)), 1.8 g (19.13 mmol) phenol could be isolated after irradiation for 48 h.

The catalytic oxidation of various benzene derivatives by OsO_2_*/2Sc­(OTf)_3_ and H_2_O_2_ has also been investigated (Table 3). Notably for alkylbenzenes, ring oxidation occurs predominantly, with <0.6% of alkyl chain oxidation, again highlighting the preference of OsO_2_*/2Sc­(OTf)_3_ for oxidation via an electron transfer mechanism to HAT. For the oxidation of cyanobenzene and nitrobenzene, the meta- and *ortho*-hydroxylated products were generated exclusively, in contrast to a previous report.^58^ We suggest that this regioselectivity is attributed to prebinding of **OsO_2_/2Sc­(OTf)_3_** with the –CN and –NO_2_ of the substrates to generate **OsO_2_/2Sc­(OTf)_3_/NCPh** and **OsO_2_/2Sc­(OTf)_3_/O_2_NPh**, respectively. This coordination would place the *ortho*–C–H bond of nitrobenzene and *meta*–C–H bond of cyanobenzene in the proximity of the Os=O moiety, resulting in the observed selective hydroxylation. A similar selective *ortho*-hydroxylation of nitrobenzene induced by intermolecular interaction has also been reported.^59^ Surprisingly, the highest product yield/TON occurs with the parent benzene, while lower TONs occur with benzenes with both electron-donating and withdrawing groups. Figure 7 shows the time trace of TON for benzene, ethylbenzene, methoxybenzene, and nitrobenzene; the initial rates for these substrates follow the order of methoxybenzene > ethylbenzene > benzene > nitrobenzene, as expected from their redox potentials/ease of oxidation. However, the order is reversed for their final TONs. We then carried out competitive experiments using an equimolar mixture of benzene and substituted benzenes (Tables S8–S10). Oxidation of both ethylbenzene (93%) and methoxybenzene (99%) occurred predominantly over benzene, as expected from an electron transfer mechanism. However, the total yields were much lower than the oxidation of benzene alone, 54% and 40% for ethylbenzene and methoxybenzene, respectively (Table S8 and S9). We attempted to understand such apparently anomalous results by employing UV/vis spectroscopy. The UV/vis spectrum after catalytic oxidation of benzene (after removal of solvent and redissolving in CH_3_CN, Figure S30) is similar to that of **OsO_2_/2Sc­(OTf)_3_**, but that of methoxybenzene shows a broad absorption around 400 nm, indicative of **Os^II^(MeCN)_2_/2Sc­(OTf)_3_**. This indicates that the low yields/TON for alkylbenzenes are due to further oxidation of the more easily oxidized alkylbenzenes by **Os^IV^O** to produce the inactive **Os^II^(MeCN)_2_/2Sc­(OTf)_3_** species.

**Table 3:**
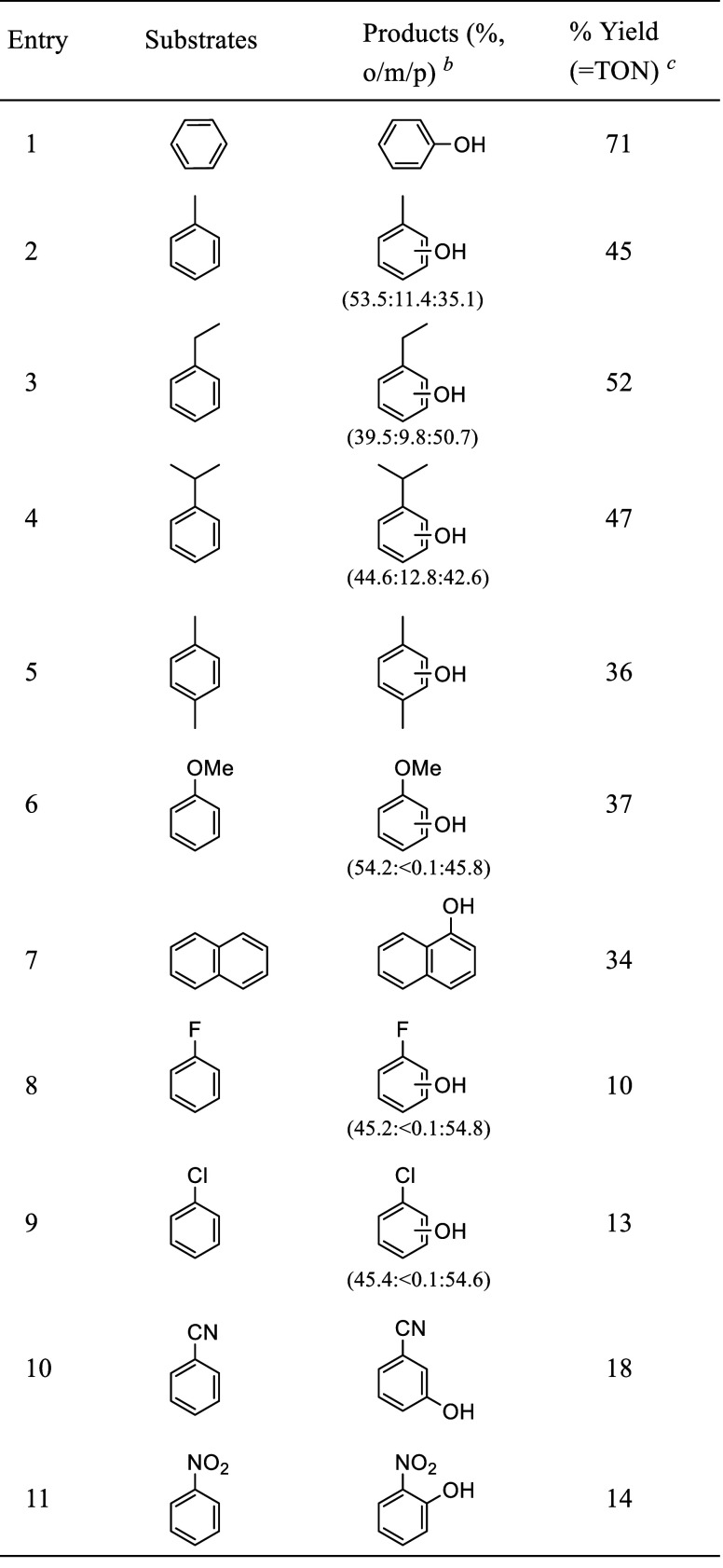
Photocatalytic Oxidation of Benzene and Derivatives by **OsO_2_** and H_2_O_2_ (100 equiv) in the Presence of 10 equiv of Sc­(OTf)_3_ ^a^

a Reaction conditions: **OsO_2_** (0.8 mM), Sc­(OTf)_3_ (8 mM), substrate (500 μL), H_2_O_2_ (80 mM) (slow addition within 4 h). All reactions were carried out in CF_3_CH_2_OH (1 mL) under argon, irradiation time at λ > 400 nm = 10 h.

b Product distribution from oxidation at ortho, meta, and para positions of benzene.

c Yield based on H_2_O_2_.

**Figure 7:**
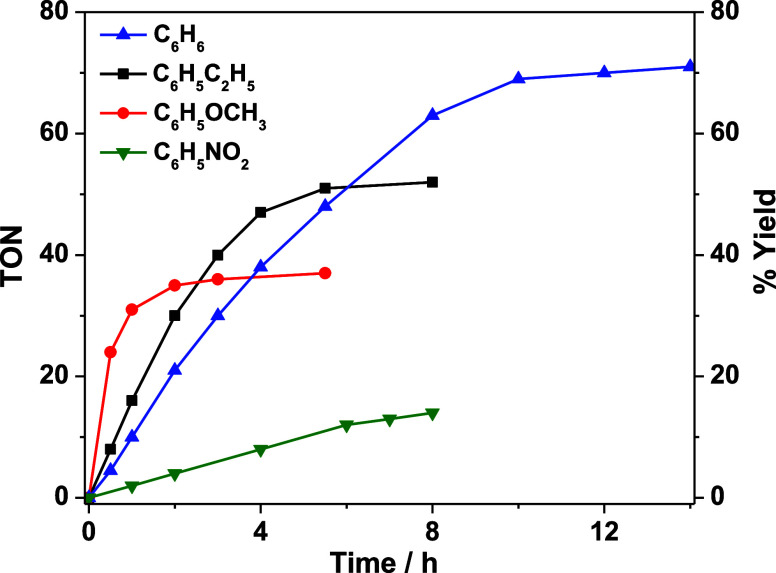
Time trace plots of photocatalytic oxidation of benzene and its derivatives. Reaction conditions: **OsO_2_** (0.8 mM), Sc­(OTf)_3_ (8 mM), substrates (500 μL), H_2_O_2_ (80 mM). All reactions were carried out in CF_3_CH_2_OH under argon. Yield based on H_2_O_2_ consumed.

### Proposed Mechanism for Photocatalytic Oxidation of Benzene

Based on the experimental results, the catalytic oxidation of benzene by OsO_2_*/2Sc­(OTf)_3_ is proposed to occur by initial electron transfer from Os^VI^O_2_*/2Sc­(OTf)_3_ to C_6_H_6_ to give **Os^V^O_2_/2Sc­(OTf)_3_** and C_6_H_6_^+•^ (Figure 8); this is followed by rapid proton transfer to generate **Os^V^O­(OH)/2Sc­(OTf)_3_** and C_6_H_5_•. O-rebound then occurs to give **Os^IV^(O)/2Sc­(OTf)_3_** and C_6_H_5_OH. An alternative pathway would involve nucleophilic attack of C_6_H_6_^+•^ by H_2_O (contained in aqueous H_2_O_2_) followed by oxidative deprotonation to produce **Os^IV^(O)/2Sc­(OTf)_3_** and C_6_H_5_OH. Rapid oxidation of the Os^IV^ species by H_2_O_2_ regenerates **OsO_2_/2Sc­(OTf)_3_**.

**Figure 8:**
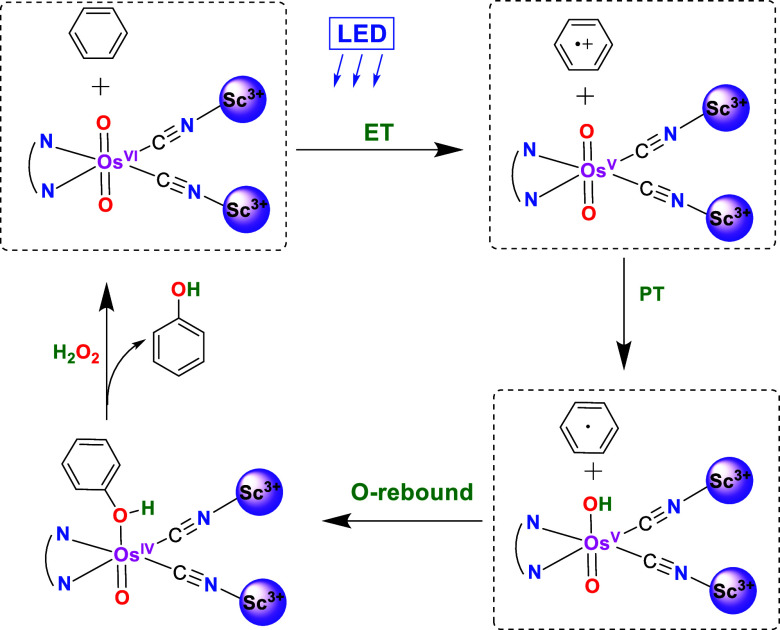
Proposed photoreaction mechanism of **OsO_2_/2Sc­(OTf)_3_** with benzene.

### DFT Calculations

DFT calculations were carried out to provide more insights into the activation of **OsO_2_** by LA.

#### Emissions of OsO_2_*/LAs

We have identified the LMCT state [*p*_π_(O^2–^) → *d*_π*_(Os^VI^)], where *d*_π*_ = *d*_*xz*_, *d*_*yz*_, as proposed by Che and co-workers.^29^ Emission originating from this proposed LMCT [*p*_π_(O^2–^) → *d*_π*_(Os^VI^)] state is calculated to peak at 648 nm for OsO_2_*, 723 nm for OsO_2_*/2BF_3_, and 733–740 nm for OsO_2_*/2Sc­(OTf)_3_. For OsO_2_*/2Sc­(OTf)_3_, multiple conformers are possible due to different coordination modes of the two scandium triflate units (Tables S11–S14). As shown in Figure 4, the measured emission maximum of OsO_2_* at ∼650 nm agrees well with the calculated value (648 nm), supporting assignment to the LMCT [*p*_π_(O^2–^) → *d*_π*_(Os^VI^)] state. However, this assignment does not account for the observed emissions in OsO_2_*/2BF_3_ and OsO_2_*/2Sc­(OTf)_3_, where the LMCT [*p*_π_(O^2–^) → *d*_π*_(Os^VI^)] emission is predicted to be substantially red-shifted (to >720 nm). We therefore examined higher-energy triplet states and found that the LMCT [π­(phen) → *d*_*yz*_(Os^VI^)] state best matches the measured emission at 608 nm for OsO_2_*/2BF_3_ (Figures S32 and S33). By analogy, we propose that the ∼650 nm emission in OsO_2_*/2Sc­(OTf)_3_ also arises from the LMCT [π­(phen) → *d*_*yz*_(Os^VI^)], although the calculated-experimental agreement is less satisfactory than for OsO_2_*/2BF_3_. Our calculations also indicate that BF_3_ or Sc­(OTf)_3_ coordination to **OsO_2_** reduces the energy gap (Δ) between the LMCT [*p*_π_(O^2–^) → *d*_π*_(Os^VI^)] and LMCT [π­(phen) → *d*_*yz*_(Os^VI^)] states.

#### Oxidation of DHA by Ground State OsO_2_ and OsO_2_/2Sc­(OTf)_3_

Figure 9 shows the potential energy surface (PES) for the oxidation mechanism of DHA by **OsO_2_** at the singlet ground state (**S_0_**). **OsO_2_** first combines with DHA to form an intermediate **INT1**, which is energetically more stable than the separated reactants. Subsequently, a hydrogen atom transfer (HAT) occurs from C9 of DHA to Os=O via transition state **TS1**, with a barrier height (Δ*G*_298_^‡^) of 18.5 kcal mol^–1^ to give intermediate **INT2**. The spin density (0.98) on the 10-hydroanthracene is consistent with a HAT process. The 10-hydroanthracene group in **INT2** then undergoes a rotation, positioning the H atom at C10 closer to the Os–OH group to give **INT3**. This is followed by another HAT process from C10 to Os–OH via **TS2**, which has a relatively low barrier height of 8.1 kcal mol^–1^ (relative to **OsO_2_** + DHA), to yield anthracene, H_2_O, and **OsO** species (**INT5**). The DFT calculations indicate that the initial HAT process is the rate-determining step, with a barrier height of 18.5 kcal mol^–1^ (relative to **OsO_2_** + DHA). The oxidation of DHA by **OsO_2_/2Sc­(OTf)_3_** follows a similar mechanism but with a significantly lower barrier of 11.4 kcal mol^–1^ (Figure S34).

**Figure 9:**
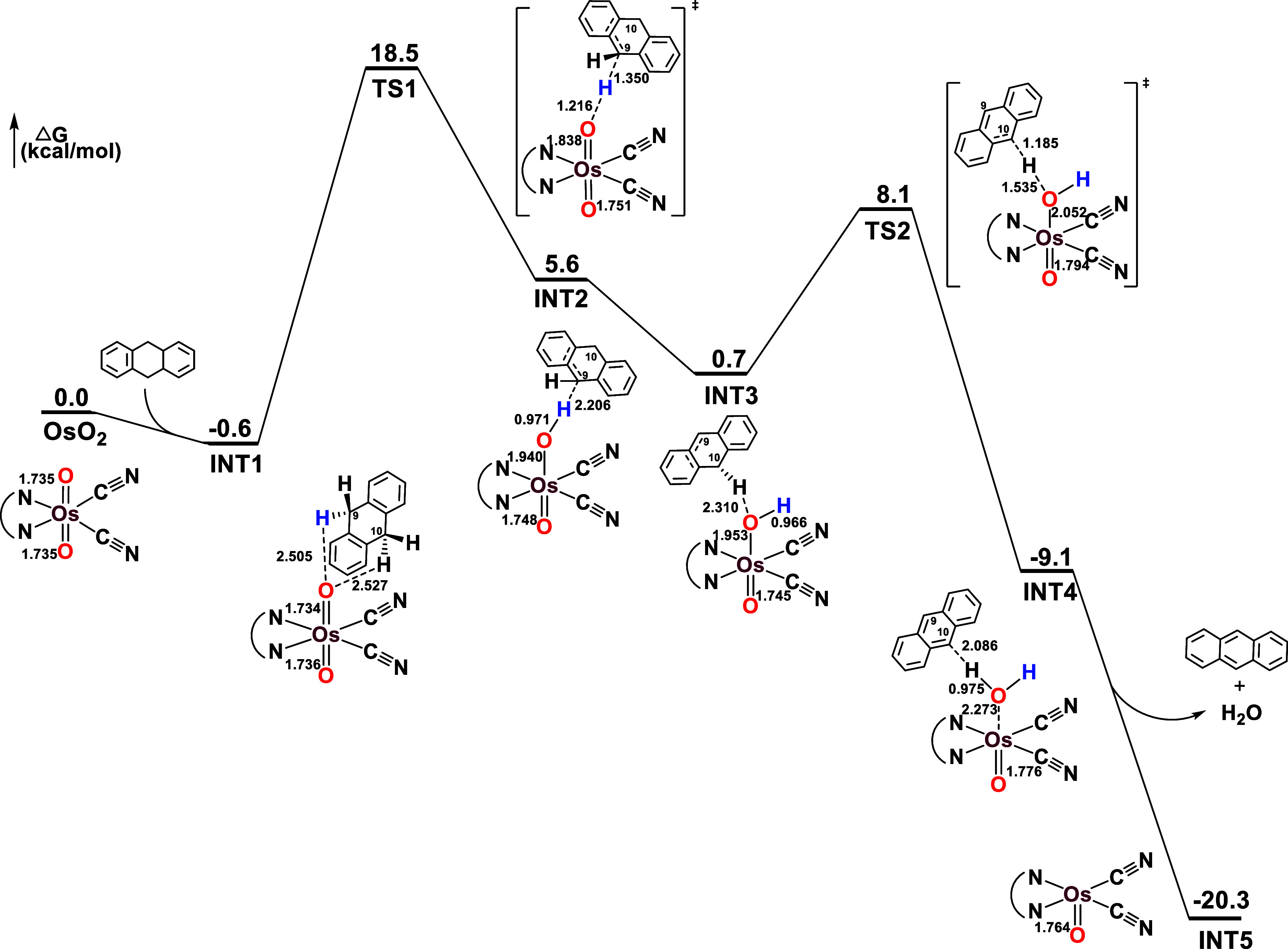
PES profile for the oxidation of DHA by **OsO_2_** (S_0_).

#### Oxidation of Cyclohexane by Excited State OsO_2_* and OsO_2_*/2Sc­(OTf)_3_

Upon light excitation, the LMCT [*p*_π_(O^2–^) → *d*_π*_(Os^VI^)] triplet state (T_1_) of **OsO_2_** is populated and is responsible for the λ_max_ = 650 nm observed in the emission spectra. Both Os=O bonds (1.804 Å) are predicted to be equivalent in ^**3**^ **OsO_2_** (T_1_), and the spin density is distributed with approximately 0.75 on the Os center and 0.57 on each oxygen atom, accounting for a total of two unpaired electrons. C_6_H_12_ and **^3^OsO_2_** first bind weakly together to form an intermediate, **^3^INT6**. Subsequently, HAT occurs from C_6_H_12_ to Os=O via transition state **^3^TS3**, generating the Os–OH species **^3^INT7**. This rate-determining step has a Δ*G*_298_^‡^ of 7.3 kcal mol^–1^, suggesting that the photoexcited **OsO_2_**, via LMCT [*p*_π_(O^2–^) → *d*_π*_(Os^VI^)] T_1_ state, can readily oxidize C_6_H_12_ under ambient conditions. The carbon atom that lost the H atom in the cyclohexyl radical carries a spin density of 0.94, supporting a HAT mechanism (Table S16). Next, an O-rebound takes place via **^3^TS4** with a small Δ*G*_298_^‡^ of only 5.7 kcal mol^–1^ (relative to **^3^INT7**), yielding **^3^INT8**, which then generates the cyclohexanol and OsO oxo species (**^3^INT5**) (Figure 10).

**Figure 10:**
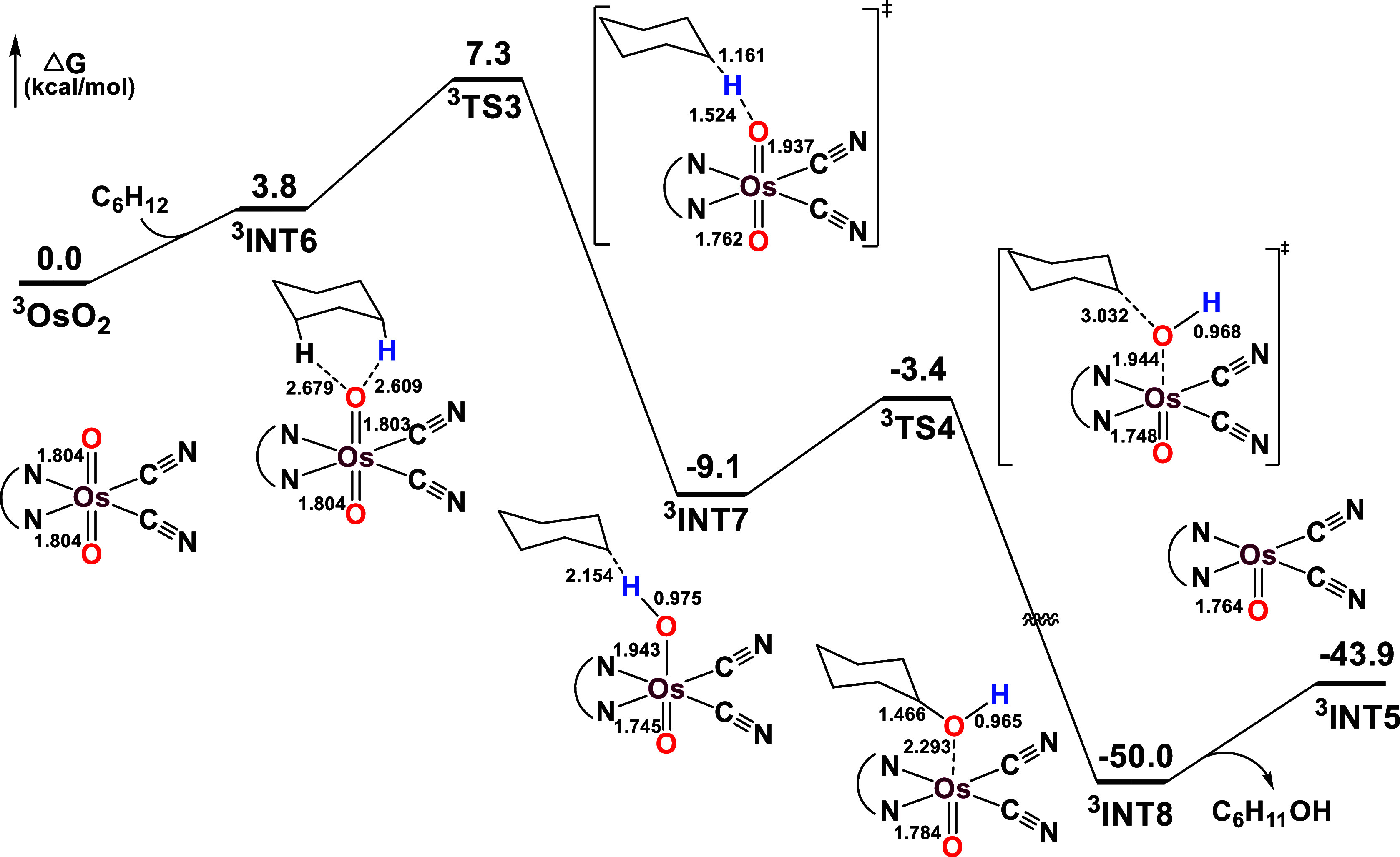
PES profile for the oxidation of cyclohexane by **^3^OsO_2_** (T_1_).

The optimized structure of **^3^OsO_2_/2Sc­(OTf)_3_** (T_1_) features nonequivalent Os=O bonds (1.924 and 1.778 Å). The spin density is unevenly distributed along the O=Os=O unit, with 0.38 localized on the Os center, 0.95 on the oxygen atom of the longer Os=O bond, and 0.56 on the oxygen atom of the shorter Os=O bond. The enhanced oxyl-like character at the oxygen of the longer Os=O bond is therefore expected to be the reactive site for HAT. Consistent with the oxidation mechanism of C_6_H_12_ by **OsO_2_**, C_6_H_12_ and **^3^OsO_2_/2Sc­(OTf)_3_** initially form a weakly bound complex (**^3^INT6/2Sc­(OTf)_3_**). This is followed by a rate-determining HAT from cyclohexane to the Os=O moiety via transition state **^3^TS3/2Sc­(OTf)_3_**, and subsequently a barrierless O-rebound step leading to the formation of **^3^INT8/2Sc­(OTf)_3_** (Table S16). The energy barrier of the HAT step is 5.3 kcal mol^–1^, moderately lower than that for the oxidation by **^3^OsO**_**2**_ alone (7.3 kcal mol^–1^) (Figure 11).

**Figure 11:**
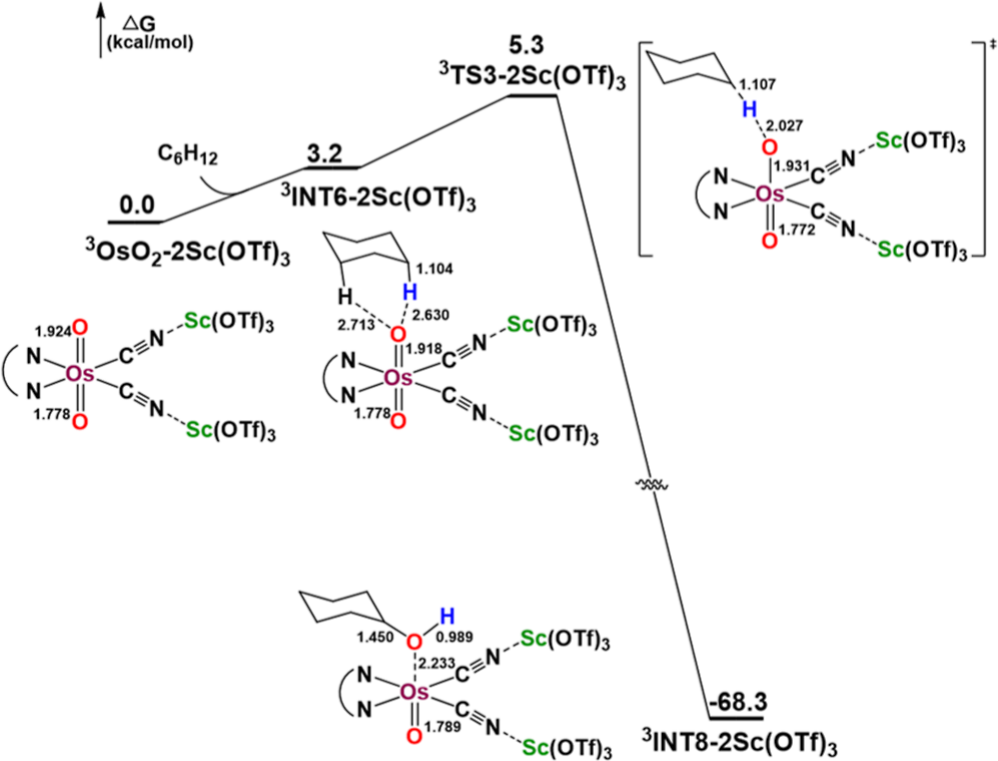
PES profile for the oxidation of cyclohexane by the **^3^OsO_2_/2Sc­(OTf)_3_** (T_1_).

## Conclusions

In conclusion, we have investigated for the first time the effects of Lewis acids on both the ground and excited states of a luminescent Os­(VI) dioxo dicyano complex with a long-lived excited state. We found that both the ground and excited states of **OsO_2_** are greatly activated by various Lewis acids via strong binding to the ancillary cyanide rather than to the oxo ligands. This enables us to isolate various **OsO_2_/LA** adducts, which to our knowledge are the first examples of isolated oxidizing M=O/LA adducts. Strong binding of LAs result in large positive shifts in the reduction potentials and oxidizing power of **OsO_2_**. **OsO_2_/2Sc­(OTf)_3_** is able to oxidize a variety of organic substrates. More significantly, OsO_2_*/2Sc­(OTf)_3_ has an *E*_pc_ of 3.50 V vs. NHE, which is probably the strongest metal oxo species generated in solution, and is higher than that of F_2_ (2.87 V). As such, it is able to oxidize benzene readily at ambient conditions to give phenol. Moreover, the oxidation can be made catalytic by using the green oxidant H_2_O_2_, with a TON up to 8660, and the gram-scale production of phenol. We have demonstrated that it is possible to generate high-oxidizing and yet stable metal oxo species through a combination of light excitation and Lewis acid activation.

## Experimental Section

### Chemicals

**OsO_2_** was synthesized from a literature procedure.^30^ All chemicals were of reagent grade unless otherwise specified. Lewis acids were stored in a drybox before use. MeCN and 2,2,2-trifluoroethanol (Aldrich, HPLC) were distilled over calcium hydride and stored over 3 Å molecular sieves. Tetrabutylammonium hexafluorophosphate ([^*n*^Bu_4_N]­PF_6_), used in electrochemistry experiments, was recrystallized from absolute ethanol three times and dried under vacuum. Organic substrates were purified according to standard purification procedures. Hydrogen peroxide (25% in H_2_O) was used as received, and the concentration was determined by KMnO_4_ redox titration.

### Instrumentation

IR spectra were obtained as KBr discs using a Nicolet 360 FTIR spectrophotometer. Elemental analysis was performed using an Elementar Vario EL Analyzer. Electrospray ionization mass spectrometry (ESI-MS) was performed using a Waters Xevo G2-XS QTof mass spectrometer. ^1^H NMR spectra were recorded on a Bruker AV400 (400 MHz) FT-NMR spectrometer. Chemical shifts (δ, ppm) are reported relative to tetramethylsilane (Me_4_Si). UV/vis spectra were recorded on a Hewlett–Packard 8453 or a Hewlett–Packard 8452A diode-array spectrophotometer. Steady-state emission spectra were measured on an Edinburgh FLS1000 photoluminescence spectrometer. The solutions were rigorously degassed on a high-vacuum line with not less than four successive freeze–pump–thaw cycles. Luminescence lifetimes were measured by using the time-correlated single-photon-counting (TCSPC) technique on Edinburgh FLS1000 in a fast MCS mode with an EPL-375 ps pulsed diode laser excitation. Luminescent quantum yields for the complexes in the solution were determined by the relative method using a 0.1 M H_2_SO_4_ solution of quinine sulfate as a standard. Gas chromatographic analyses were performed on Zhejiang Fuli GC 9790 II equipped with a DB-5MS column (30 m × 0.25 mm i.d.) or a DB-FFAP column (30 m × 0.25 mm i.d.) and analysis on the FL97Plus GC workstation. GC/MS measurements were carried out on an Agilent 7890A gas chromatograph interfaced to a Agilent 5975C mass selective detector.

### X-Ray Structure Determination

A crystal with a suitable size mounted on a nylon loop circle was used for X-ray diffraction analysis. X-ray diffraction data were collected using multiscan mode at 173 K on a Bruker D8 QUEST diffractometer using monochromatized Cu Kα radiation (λ = 1.5418 Å). The data were processed, and an absorption correction was done by the multiscan method using APEX 4. Selected bond angles and bond lengths are given in Table S2. The structure was solved and refined using full-matrix least-squares based on the program SHELXT-2014 incorporated with Olex2. The positions of the other non-hydrogen atoms were located after refinement by full-matrix least-squares using the SHELXL-2016. All non-hydrogen atoms were refined anisotropically. Hydrogen atoms were generated by the SHELXL-2016. The positions of hydrogen atoms were calculated based on the riding mode with thermal parameters equal to 1.2 times that of the associated C atoms and participated in the calculation of final R indices.

### Photocatalytic Experiments

All photocatalysis experiments were carried out under an argon atmosphere unless otherwise specified. In a typical experiment, H_2_O_2_ (80 mM) was slowly added to a pale-yellow degassed solution of the **OsO_2_** catalyst (0.8 mM) and LA (8 mM) in CF_3_CH_2_OH (1 mL) containing a substrate (500 μL) at 23 °C. The mixture solution was stirred and irradiated at >400 nm light for 10 h. 1,4-dichlorobenzene was then added as an internal standard, and the organic products were identified and quantified by GC/MS and GC/FID at various time intervals. The kinetic isotope effect (KIE) was investigated by using an equimolar mixture of benzene and d^6^-benzene, and the value was calculated by taking the ratio of the corresponding peak areas of nondeuterated and deuterated products from GC measurements.

### Synthesis

#### [Os^VI^(O)_2_(dpphen)­(CN–B­(C_6_F_5_)_3_)_2_] (OsO_2_/2B­(C_6_F_5_)_3_)

To a solution of **OsO_2_** (12 mg, 0.02 mmol) in CH_2_Cl_2_ (2 mL) was added B­(C_6_F_5_)_3_ (25.6 mg, 0.05 mmol), the resulting yellow solution was stirred for 15 min at 23 °C. Slow diffusion of *n*-pentane into the solution for 2 days afforded brown plate crystals of **OsO_2_/2B­(C_6_F_5_)_3_** suitable for X-ray diffraction. Yield: 15 mg, 46%. Anal. Calcd For C_62_H_16_B_2_F_30_N_4_O_2_Os: C, 45.67; H, 0.99; N, 3.44. Found: C, 45.32; H, 1.05; N, 3.49. UV–vis (in CH_2_Cl_2_) λ_max_[nm] (ε/M^–1^ cm^–1^): 290 (25100), 338sh (8210), 367sh (3010). Selected IR (KBr disc, cm^–1^): *v*(C≡N) 2260, *v*(C–F) 1105, *v*(B–C) 978, *v*(Os=O) 856. ^1^H NMR (400 MHz, CDCl_3_): δ 9.86 (d, *J* = 5.5 Hz, 2H), 8.40 (s, 2H), 8.17 (d, *J* = 5.7 Hz, 2H), 7.65–7.75 (dd, *J* = 32.5, 6.0 Hz, 10H).

#### [Os^VI^(O)_2_(dpphen)­(CN)]­(μ-CN)­[Zn­(Cl)_2_(MeCN)] (OsO_2_/Zn)

To a solution of **OsO_2_** (12 mg, 0.02 mmol) in MeCN (5 mL) was added anhydrous ZnCl_2_ (6.82 mg, 0.05 mmol), followed by sonication for 5 min. An orange precipitate was immediately formed, which was filtered, and slow evaporation of the filtrate afforded yellow needle crystals of the dinuclear complex [Os^VI^(O)_2_(dpphen)­(CN)]­(μ-CN)­[Zn­(Cl)_2_(MeCN)] (**OsO_2_/Zn**) suitable for X-ray diffraction analysis. Yield: (5 mg, 31.6%). Anal. Calcd For C_28_H_19_Cl_2_N_5_O_2_OsZn: C, 42.90; H, 2.44; N, 8.93. Found: C, 42.59; H, 2.35; N, 8.59. UV–vis (in CH_2_Cl_2_) λ_max_[nm] (ε/M^–1^ cm^–1^): 290 (23950), 330sh (6800), 367sh (1870). Selected IR (KBr disc, cm^–1^): *v*(C≡N) 2216, *v*(Os=O) 849. ^1^H NMR (400 MHz, CDCl_3_, shown below): δ 10.20–10.16 (m, 2H), 8.25 (s, 2H), 8.11 (dd, *J* = 5.3, 2.7 Hz, 2H), 7.70–7.59 (m, 10H).

#### [Os^VI^(O)_2_(dpphen)­(CN)­(CN···TFA)] (OsO_2_/TFA)

To a solution of **OsO_2_** (12 mg, 0.02 mmol) in CH_2_Cl_2_ (2 mL) was added TFA (1.53 μL, 0.02 mmol); the resulting yellow solution was stirred for 15 min. Slow diffusion of *n*-pentane into the solution for 2 days afforded brown block crystals of **OsO_2_/TFA** suitable for X-ray diffraction. Yield: 9 mg, 65%. Anal. Calcd For C_28_H_17_F_3_N_4_O_4_Os: C, 46.66; H, 2.38; N, 7.77. Found: C, 46.23; H, 2.25; N, 7.56. UV–vis (in CH_2_Cl_2_) λ_max_[nm] (ε/M^–1^ cm^–1^): 290 (24510), 330sh (7980), 367sh (2080). Selected IR (KBr disc, cm^–1^): *v*(C≡N) 2179, 2158, *v*(Os=O) 853. ^1^H NMR (400 MHz, CDCl_3_): δ 9.86 (d, *J* = 5.5 Hz, 2H), 8.40 (s, 2H), 8.18 (d, *J* = 6.0 Hz, 2H), 7.76–7.64 (m, 10H).

#### [Os^VI^(O)_2_(dpphen) (CN···TFA)_2_] (OsO_2_/2TFA)

The synthetic route is similar to that of OsO_2_/TFA except that 2 equiv. TFA (3.1 μL, 0.04 mmol) was added instead. Yield: 9 mg, 55%. Anal. Calcd For C_30_H_18_F_6_N_4_O_6_Os: C, 43.17; H, 2.17; N, 6.71. Found: C, 43.53; H, 2.11; N, 6.69. UV–vis (in CH_2_Cl_2_) λ_max_[nm] (ε/M^–1^ cm^–1^): 290 (24490), 330sh (8010), 367sh (2490). Selected IR (KBr disc, cm^–1^): *v*(C≡N) 2179, 2164, *v*(Os=O) 851. ^1^H NMR (400 MHz, CDCl_3_): δ 9.87 (d, *J* = 5.5 Hz, 2H), 8.40 (s, 2H), 8.17 (d, *J* = 6.0 Hz, 2H), 7.75–7.64 (m, 10H).

#### [Os^II^(MeCN)_2_(dpphen)­(CN)_2_(Sc^III^)_2_(CF_3_SO_3_)_6_] (Os^II^(MeCN)_2_/2Sc­(OTf)_3_)

To a solution of **OsO_2_** (20 mg, 33 μmol) and ferrocene (61 mg, 330 μmol) in MeCN (10 mL) was added 10 equiv of Sc­(OTf)_3_ (162 mg, 330 μmol) at room temperature. Upon adding Sc­(OTf)_3_, the solution was immediately turned from pale yellow to green, and was further stirred for 6 h gave a brown solution. The solution was evaporated under reduced pressure, and the residue was washed with diethyl ether and a minimum amount of MeCN to remove excess Sc­(OTf)_3_ and Fc/Fc^+^. The residue was then redissolved in CH_2_Cl_2_ and filtered to give a brownish-yellow solution. **Os^II^(MeCN)_2_/2Sc­(OTf)_3_** was isolated as an orange-yellow solid by slow diffusion of *n*-pentane to the CH_2_Cl_2_ solution. Yield: 17 mg, 34.8%. Anal. Calcd For C_36_H_22_F_18_N_6_O_18_OsS_6_Sc_2_: C, 26.35; H, 1.35; N, 5.12. Found: C, 26.31; H, 1.38; N, 5.15. UV–vis (in MeCN) λ_max_[nm] (ε/M^–1^ cm^–1^): 276 (24030), 380 (5140), 440sh (2010). Selected IR (KBr disc, cm^–1^): *v*(C≡N) 2080. ESI-MS (+ve mode) in MeCN: *m*/*z* 659.1 ([Os^II^(MeCN)_2_(dpphen)­(CN)_2_ + H])^+^. ^1^H NMR (400 MHz, CDCl_3_): δ 9.37 (d, 2H), 8.10 (s, 2H), 7.90 (d, 2H), 7.82–7.55 (m, 10H), 2.84 (s, 6H, CH_3_CN).

#### {[Os^II^(MeCN)_2_(dpphen)­(CN–B­(C_6_F_5_)_3_)_2_} (Os^II^(MeCN)_2_/2B­(C_6_F_5_)_3_)

The synthesis of **Os^II^(MeCN)_2_/2B­(C_6_F_5_)_3_** is similar to that of Os^II^(MeCN)_2_/2Sc­(OTf)_3,_ except that 5 equiv. B­(C_6_F_5_)_3_ (84 mg, 165 μmol) was used instead of Sc­(OTf)_3_. The brown residue obtained by evaporation of the solution was recrystallized by vapor diffusion of ether to its MeCN solution for 2 days to afford brown prism crystals of **Os^II^(MeCN)_2_/2B­(C_6_F_5_)_3_**. Yield: 34 mg, 69.7%. Anal. Calcd For C_66_H_22_B_2_F_30_N_6_Os: C, 47.17; H, 1.32; N, 5.00. Found: C, 47.23; H, 1.21; N, 4.94. UV–vis (in MeCN): λ_max_ [nm] (ε/M^–1^ cm^–1^): 275 (35960), 380 (8390), 442sh (3820). Selected IR (KBr disc, cm^–1^): *v*(C≡N) 2146, 2180. ^1^H NMR (400 MHz, CDCl_3_): δ 9.41 (d, 2H), 8.10 (s, 2H), 7.85 (d, 2H), 7.67–7.59 (m, 10H), 2.85 (s, 6H, CH_3_CN).

#### [Os^IV^(O)­(MeCN)­(dpphen)­(CN)_2_(Sc^III^)_2_(CF_3_SO_3_)_6_] (Os^IV^O/2Sc­(OTf)_3_)

Five Pyrex tubes (15 × 2 cm) each containing **OsO_2_** (5 mg, 8.2 μmol) and 10 equiv. Sc­(OTf)_3_ (41 mg, 82 μmol) in the presence of benzene (500 μL) in MeCN (10 mL) was prepared. Each tube was sealed with a rubber septum, degassed with Ar for 30 min, and then irradiated with LED light (λ > 400 nm) for 3 h, whereby the light-yellow solution turned dark green. The solutions were combined, and the solvent was removed under reduced pressure. The solid residue was then washed with diethyl ether and a minimum amount of MeCN to afford a dark green solid. Brown microcrystalline solid of **Os^IV^O/2Sc­(OTf)_3_** was then obtained by slow diffusion of diethyl ether into a CH_2_Cl_2_ solution. Yield: 34 mg, 51.3%. Anal. Calcd For C_34_H_19_F_18_N_5_O_19_OsS_6_Sc_2_: C, 25.27; H, 1.19; N, 4.33 Found: C, 25.24; H, 1.24; N, 4.28. UV–vis (in MeCN) λ_max_ [nm] (ε/M^–1^ cm^–1^): 284 (32730), 330sh (7740), 430sh (1390). Selected IR (KBr disc, cm^–1^): *v*(C≡N) 2003 and 2069 cm^–1^; *v*(Os=O) 855 cm^–1^. ESI-MS (-ve mode) in 2,2,2-trifluoroethanol: *m*/*z* 1273.8 ([Os^IV^(O)­(MeCN)­(dpphen)­(CN)_2_(Sc^III^)­(CF_3_SO_3_)_4_]^−^).

## Supplementary Material



## Notes

Detailed experimental methods, including characterization data, spectra, and computational studies (PDF)
