# Supplementary material for: Activation of the Ground and Excited State of a Luminescent Osmium(VI) Dioxo Dicyano Complex with Lewis Acids
Source: J Am Chem Soc. 2026 May 4;148(19):20094–106. doi: 10.1021/jacs.6c04322 (PMC13195676; doi:10.1021/jacs.6c04322)
Supplement: Supplementary file 1 [file ja6c04322_si_001.pdf]

Activation of the Ground and Excited State of a Luminescent Osmium(VI) Dioxo Dicyano Complex with Lewis Acids

Li-Xin Wang,[1,†] Fang Xu,[1,†] Ailin Gao,[2,†] Jing Xiang,[1*] Yi Pan,[2] Rui-Yue Qi,[1] Ji-Yan Liu,[1] Tian-Ci Li,[1] Kai-Chung Lau,[2*] and Tai-Chu Lau[2*]

[1] Key Laboratory of Optoelectronic Chemical Materials and Devices (Ministry of Education), School of Optoelectronic Materials and Technology, Jianghan University, Wuhan, China.

[2] Department of Chemistry, City University of Hong Kong, Tat Chee Avenue, Kowloon Tong, Hong Kong, P. R. China

✉*e-mail: xiangjing35991@sohu.com (J. X.), bhtclau@cityu.edu.hk (T.-C. L.)*

Table of Contents

Fig. S1. (a) CV of **OsO_2** (0.8 mM) in the presence of various amounts of $Sc(OTf)_3$. (b) Plot of $Os^{VI/V}$ redox couples *vs.* equivalents of Sc^{3+}. **S1**

Table S1. The $Os^{VI/V}$ redox potentials of **OsO_2** (0.8 mM) in the presence of various equivalents of $Sc(OTf)_3$. **S1**

Fig. S2. CV of (a) **OsO_2**, (b) **$OsO_2/2B(C_6F_5)_3$**, (c) **OsO_2/Zn**, (d) **OsO_2/TFA**, (e) **OsO_2/2TFA** and (f) **OsO_2/2HOTf** in CH_3CN. **S2**

Fig. S3. ESI/MS (-ve mode) of the solution of **OsO_2** with 5 equiv. $Sc(OTf)_3$ in CF_3CH_2OH. **S3**

Fig. S4. ESI/MS (-ve mode) of the solution of **OsO_2** with 5 equiv. $Zn(OTf)_2$ in CF_3CH_2OH. **S3**

Fig. S5. ESI/MS (+ve mode) of the solution of **$Os^{II}(MeCN)_2/2Sc(OTf)_3$** in CF_3CH_2OH. **S4**

Fig. S6. IR spectra of (a) **OsO_2**, (b) **$OsO_2/2B(C_6F_5)_3$**, (c) **OsO_2/Zn**, (d) **OsO_2/TFA**, (e) **OsO_2/2TFA** and (f) **$Os(MeCN)_2/2B(C_6F_5)_3$.** **S5**

Fig. S7. IR spectra of adducts of **OsO_2** with (a) $Sc(OTf)_3$, (b) $Mg(OTf)_2$, (c) $Lu(OTf)_3$, and (d) $Zn(OTf)_2$. **S5**

Fig. S8. UV-vis spectra of **OsO_2**, **$OsO_2/2B(C_6F_5)_3$**, **OsO_2/TFA** and **OsO_2/Zn** in CH_2Cl_2 **S6**

Fig. S9. (a) UV-vis spectra of **OsO_2** with 10 equiv. of LAs or TFA in MeCN. (b) UV/vis spectral change of **OsO_2** with addition of various amounts of (a) $Sc(OTf)_3$ and (b) $B(C_6F_5)_3$ in MeCN. **S6**

Fig. S10. ^1H NMR (400 MHz) of **OsO_2** in $CDCl_3$. **S7**

Fig. S11. ^1H NMR (400 MHz) of **OsO_2/Zn** in $CDCl_3$ **S7**

Fig. S12. ^1H NMR (400 MHz) of **OsO_2/TFA** in $CDCl_3$. **S8**

Fig. S13. ^1H NMR (400 MHz) of **$OsO_2/2B(C_6F_5)_3$** in $CDCl_3$. **S8**

Table S2. Selected bond parameters (Å, °) of **OsO_2, $OsO_2/2B(C_6F_5)_3$**, **OsO_2/Zn**, **$Os(MeCN)_2/2B(C_6F_5)_3$** and **OsO_2/TFA** **S9**

Fig. S14. The emission spectral change of **OsO_2** by addition of various amounts of (a) BF_3 (b) $Sc(OTf)_3$ and (c) TFA in MeCN solution at 298 K. **S9**

Fig. S15. (a) UV-vis spectral changes for the reaction of **OsO_2** (3.29×10^{-5} M) with bromoferrocene (3.29×10^{-4} M) in the presence of $Sc(OTf)_3$ (3.29×10^{-4} M) at 298 K. (b) Plots of the k_{obs} *vs.* the concentration of bromoferrocene. **S10**

Fig. S16. (a) UV-vis spectral changes for the reaction of **OsO_2** (3.29×10^{-5} M) with PPh_3 (3.29×10^{-4} M) in the presence of $Sc(OTf)_3$ (3.29×10^{-4} M) at 298 K. (b) Plot of the k_{obs} against the concentration of PPh_3. **S10**

Fig. S17. ESI-MS for the reaction of **OsO_2** (6.5×10^{-5} M) with 100 eq. PPh_3 in the presence of 10 eq. $Sc(OTf)_3$ (6.5×10^{-4} M) in MeCN at 298 K. **S11**

Fig. S18. (a) UV-vis spectral changes for the reaction of **OsO_2** (3.29×10^{-5} M) with hydroquinone (6.58×10^{-3} M) in the presence of $Sc(OTf)_3$ (3.29×10^{-4} M) at 298 K. (b) Plot of the k_{obs} against the concentration of hydroquinone. **S11**

Fig. S19. (a) UV-vis spectral changes for the reaction of **OsO_2** (3.29×10^{-5} M) with hydroquinone (1.15×10^{-2} M) in the presence of $Sc(OTf)_3$ (3.29×10^{-4} M) in MeCN/H_2O (v/v = 18:1) or (c) MeCN/D_2O (v/v = 18:1) at 298 K. Plots of the k_{obs} against the concentration of (b) hydroquinone or (d) deuterated hydroquinone. **S12**

Fig. S20. (a) UV-vis spectral changes for the reaction of **OsO_2** (3.29×10^{-5} M) with xanthene (9.87×10^{-4} M) in the presence of $Sc(OTf)_3$ (3.29×10^{-4} M). (b) Plots of the initial rate R_x against the concentration of xanthene or d^2-xanthene. **S12**

Fig. S21. (a) UV-vis spectral changes for the reaction of **OsO_2** (3.29×10^{-5} M) with 9,10-dihydroanthracene (3.29×10^{-2} M) in the presence of $Sc(OTf)_3$ (3.29×10^{-4} M). (b) Plots of the k_{obs} against the concentration of 9,10-Dihydroanthracene. **S13**

Estimation of excited state redox potentials of OsO_2 and OsO_2/2LA. **S13**

Table S3. Photocatalytic oxidation of cyclohexane by **OsO_2**/H_2O_2 in the presence of 10 equiv. of $Sc(OTf)_3$. **S13**

Table S4. Photocatalytic oxidation of benzene to phenol by **OsO_2**/H_2O_2 in the presence of different amounts of $Sc(OTf)_3$. **S14**

Table S5. Photocatalytic oxidation of benzene to phenol by **OsO_2**/H_2O_2 in the presence of various LAs. **S14**

Table S6. Competitive oxidation of an equimolar mixture of cyclohexane and benzene by **$OsO_2/2Sc(OTf)_3$**/H_2O_2. **S14**

Table S7. Photocatalytic oxidation of benzene by **$OsO_2/2Sc(OTf)_3$**/H_2O_2. **S15**

Table S8. Competitive oxidation of an equimolar mixture of ethylbenzene and benzene by **$OsO_2/2Sc(OTf)_3$**/H_2O_2. **S15**

Table S9. Competitive oxidation of an equimolar mixture of anisole and benzene by **$OsO_2/2Sc(OTf)_3$**/H_2O_2. **S15**

Table S10. Competitive oxidation of an equimolar mixture of nitrobenzene and benzene by **OsO_2**/$Sc(OTf)_3$/H_2O_2. **S15**

Fig. S22. GC/MS of phenol and d^5-phenol obtained from photocatalytic reaction. **S16**

Fig. S23. UV-vis spectrum of **$Os^{IV}O/2Sc(OTf)_3$** in MeCN. **S16**

Fig. S24. IR spectrum of **$Os^{IV}O/2Sc(OTf)_3$.** **S17**

Fig. S25. ESI-MS of **$Os^{IV}O/2Sc(OTf)_3$.** **S17**

Fig. S26. UV-vis spectrum of **$Os^{II}(MeCN)_2/2B(C_6F_5)_3$** in MeCN. **S18**

Fig. S27. UV-vis spectrum of **$Os^{II}(MeCN)_2/2Sc(OTf)_3$** in MeCN. **S18**

Fig. S28. The UV/vis spectra of **$Os^{VI}O_2/2Sc(OTf)_3$**, **$Os^{IV}O/2Sc(OTf)_3$**, and the addition of H_2O_2 into a CH_3CN solution of **$Os^{IV}O/2Sc(OTf)_3$.** **S19**

Fig. S29. (a) The UV/vis spectral change for the reaction of **$Os^{II}(MeCN)_2/2Sc(OTf)_3$** with H_2O_2. (b) CV of **$Os^{IV}O/2Sc(OTf)_3$** and **$Os^{II}(MeCN)_2/2Sc(OTf)_3$** in MeCN. **S20**

Fig. S30. UV/vis spectra after photocatalytic oxidation of anisole and benzene in MeCN. **S21**

Fig. S31. UV/vis spectral changes for the photoreaction of **$OsO_2/2Sc(OTf)_3$** with nitrobenzene (a) and ethylbenzene (b) in MeCN. **S21**

Table S11. Optimized Os=O and Os-CN bond lengths (in Å) and ∠NC-Os-CN angles (°) calculated at the B3LYP-D3(BJ)/def2-SVP level with PCM (dichloromethane). **S23**

Table S12. The selected orbital energies (*E*, in Hartrees) and energy splitting (in eV) of the optimized S_0 structures calculated at the B3LYP-D3(BJ)/def2-SVP level with PCM (dichloromethane). **S23**

Table S13. The emission energies (λ in nm) for $T_1 \rightarrow S_0$ of **OsO_2**, **OsO_2/BF_3**, **$OsO_2/2Sc(OTf)_3$** calculated at the B3LYP-D3(BJ)/def2-SVP level with PCM (acetonitrile). **S24**
Table S14. Orbital contributions in the $S_0 \rightarrow T_1$ transition, from NTO analysis at the optimized S_0 structures, at the TD-B3LYP-D3(BJ)/def2-SVP level with PCM (acetonitrile) **S24**
Fig. S32. NTO (natural transition orbital) analysis of LMCT $[p_{\pi}(O^{2-}) \rightarrow d_{\pi*}(Os^{VI})]$ transition at the optimized S_0 structure of **$OsO_2/2BF_3$** complex. **S25**
Fig. S33. NTO analysis of LMCT $[\pi(\text{phen}) \rightarrow d_{yz}(Os^{VI})]$ transition at the optimized S0 structure of **$OsO_2/2BF_3$** complex. **S26**
Fig. S34. PES profile for the oxidation of DHA by **$OsO_2/2Sc(OTf)_3$** (S_0). **S26**
Table S15. Comparison of the Gibbs free energy for different coordination modes of two OTf^- ligands in **$OsO_2/2Sc(OTf)_3$** calculated at the B3LYP-D3(BJ)/def2-SVP level with PCM (dichloromethane) **S27**
Table S16. Optimized structures of **OsO_2** and the most stable **$OsO_2/2Sc(OTf)_3$** complex (S_0 and T_1), together with the corresponding TS for the HAT step. **S27**
The Cartesian coordinates of transition states and intermediates. **S28**

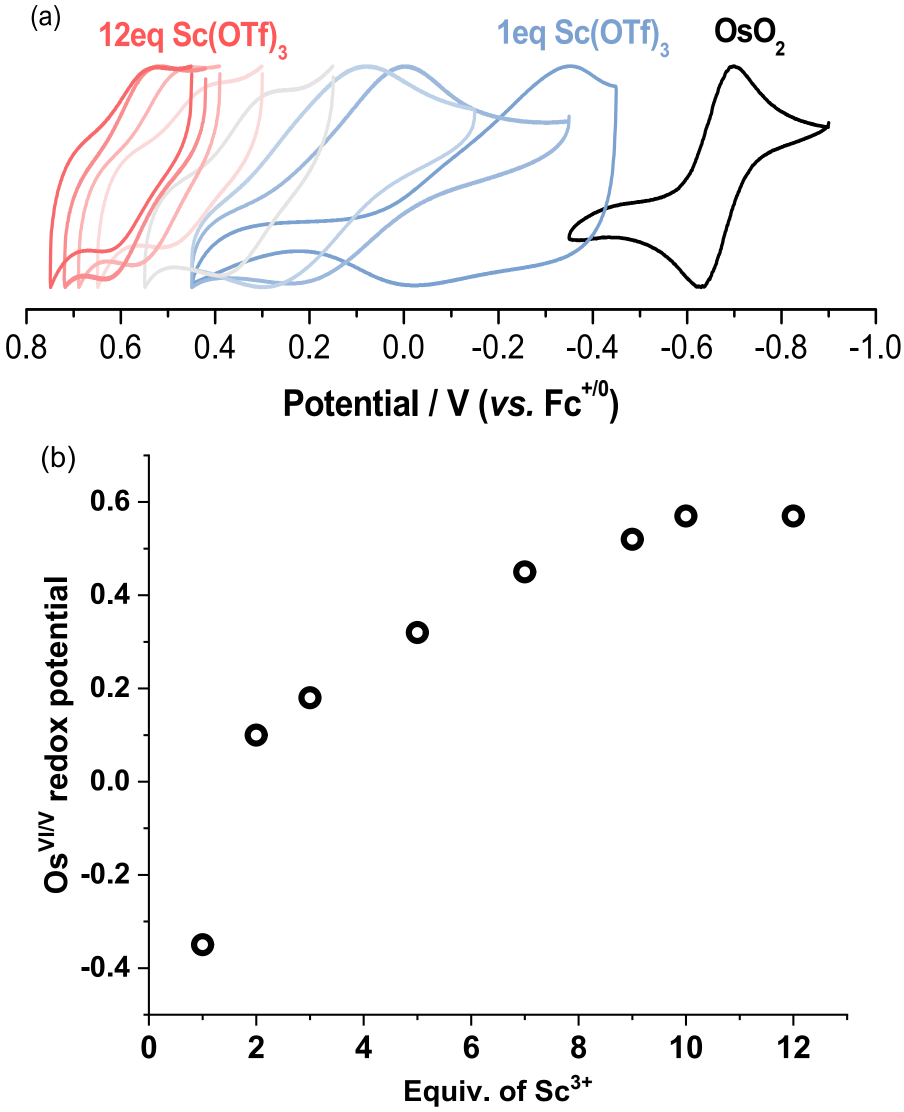

Figure S1. (a) CV of **OsO_2** (0.8 mM) in the presence of various amounts of $Sc(OTf)_3$ in 0.1 *M* [$^nBu_4N$]PF_6 degassed CH_3CN solution at 23 °C. Scan rate = 0.1 V s^{-1}. (b) Plot of $Os^{VI/V}$ redox couples *vs.* equivalents of Sc^{3+}.

Table S1. The $Os^{VI/V}$ redox potentials of **OsO_2** (0.8 mM) in the presence of various equivalents of $Sc(OTf)_3$ in degassed MeCN at 298 K.

Equiv. of $Sc(OTf)_3$	$E_{1/2}$ / V (*vs.* NHE)
nil	-0.04 V
1	0.29 V
2	0.74 V
3	0.82 V
5	0.96 V
7	1.09 V
9	1.16 V
10	1.21 V
12	1.21 V

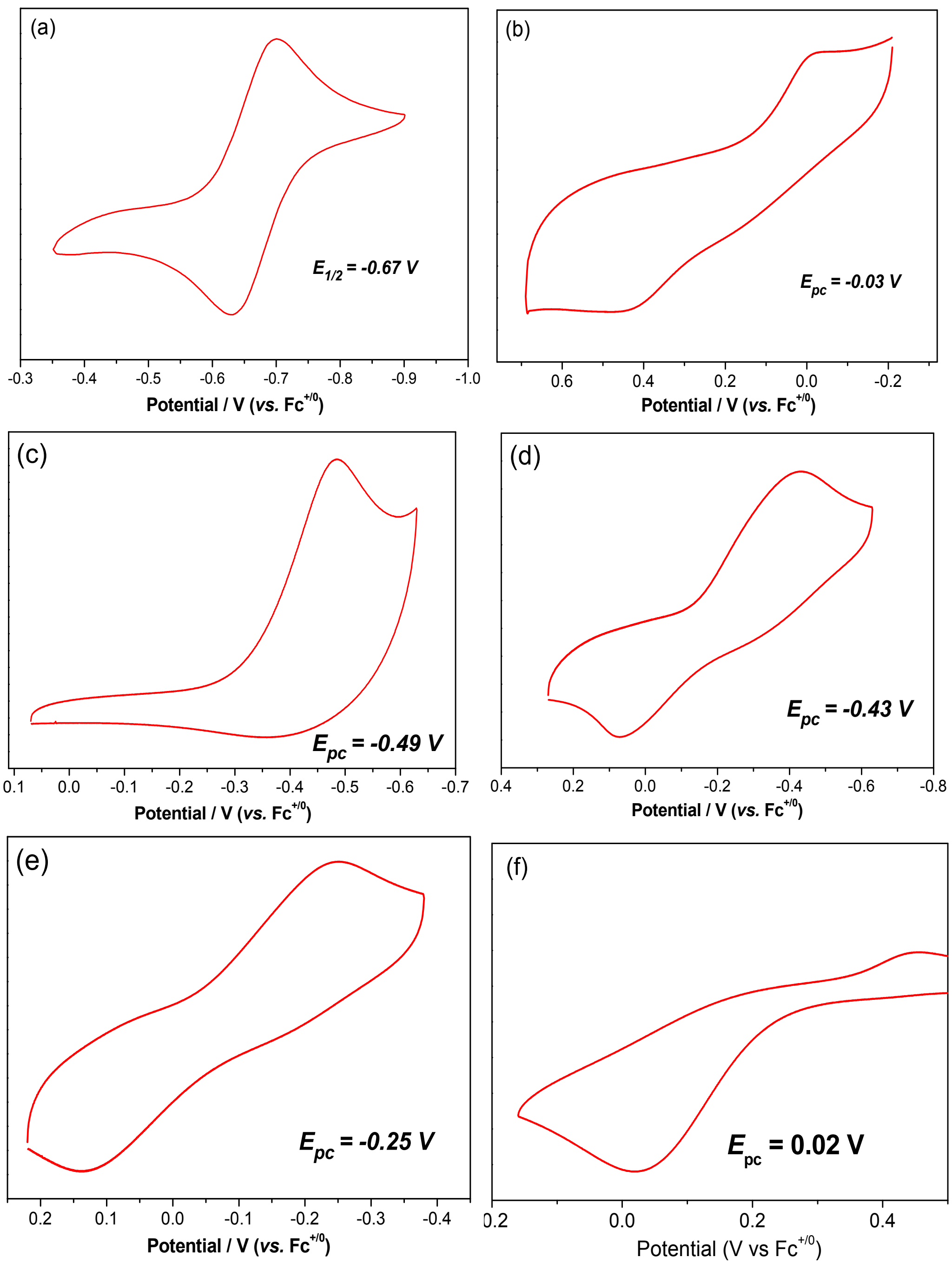

Figure S2. CV of (a) **OsO_2**, (b) **OsO_2/2B(C_6F_5)$_3$**, (c) **OsO_2/Zn**, (d) **OsO_2/TFA**, (e) **OsO_2/2TFA** and (f) **OsO_2/2HOTf** in 0.1 *M* [$^{n}Bu_4N$]PF_6 in degassed CH_3CN at 298 K. Scan rate = 0.1 V s^{-1}.

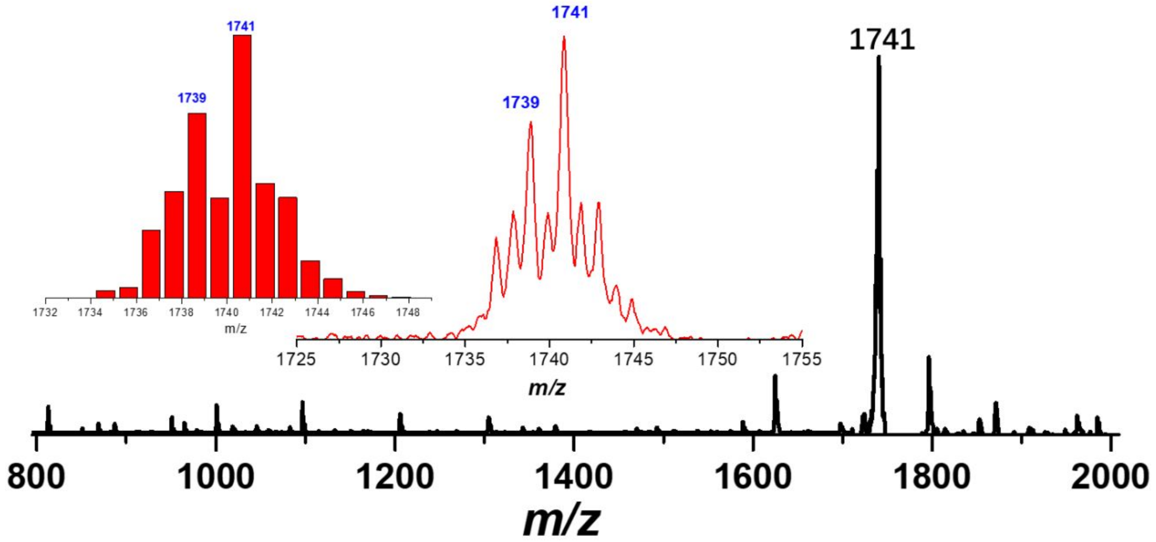

Figure S3. ESI-MS (-ve mode) of the solution of $\mathbf{OsO_2}$ with 5 equiv. of $Sc(OTf)_3$ in CF_3CH_2OH show a peak at *m/z* 1741, assigned to $[Os^{VI}(O)_2(dpphen)\ (CN)_2(Sc^{III})_2(CF_3SO_3)_7]^-$.

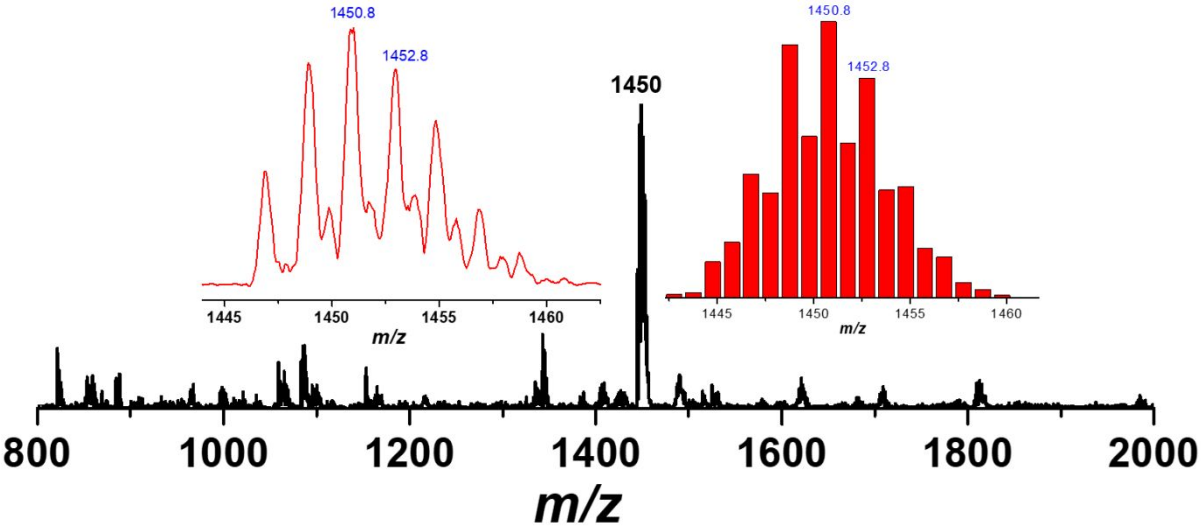

Figure S4. ESI-MS (-ve mode) of the solution of $\mathbf{OsO_2}$ with 5 equiv. of $Zn(OTf)_2$ in CF_3CH_2OH. The peak at *m/z* 1450.8 is assigned to $[Os^{VI}(O)_2(dpphen)(CN)_2(Zn^{II})_2(CF_3SO_3)_4(CF_3CH_2O)(H_2O)]^-$.

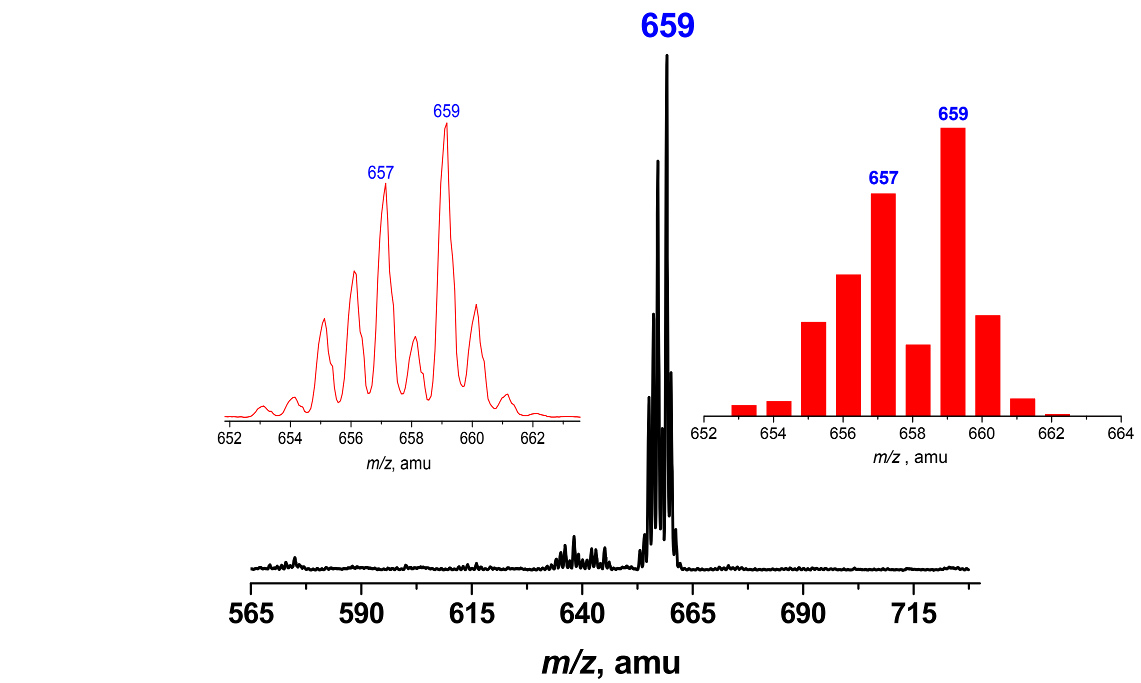

Figure S5. ESI-MS (+ve mode) of the solution of **$Os^{II}(MeCN)_2/2Sc(OTf)_3$** in CF_3CH_2OH shows a predominant peak at *m/z* 659, which is assigned to the species of $[Os^{II}(MeCN)_2(dpphen)(CN)_2 + H]^+$.

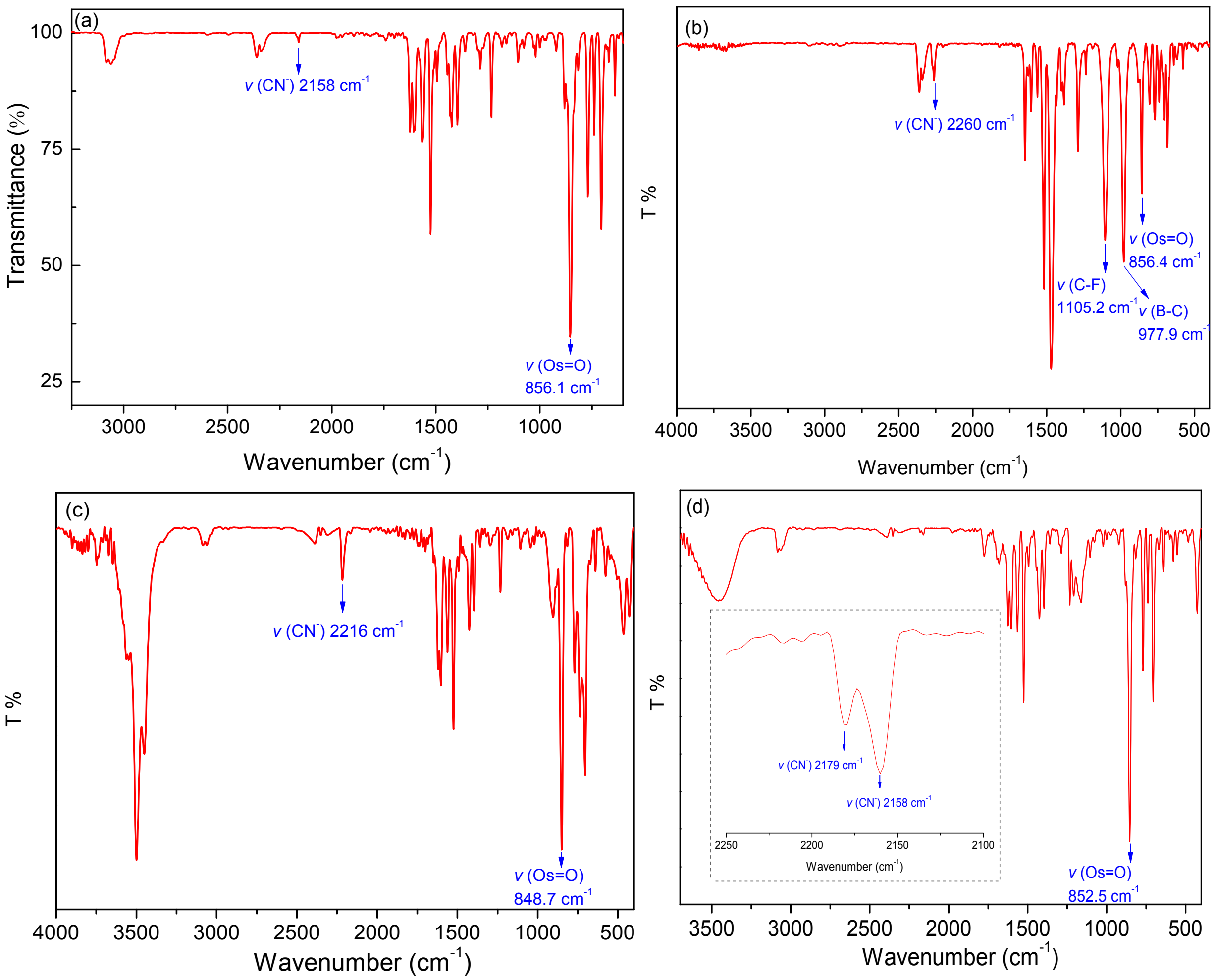

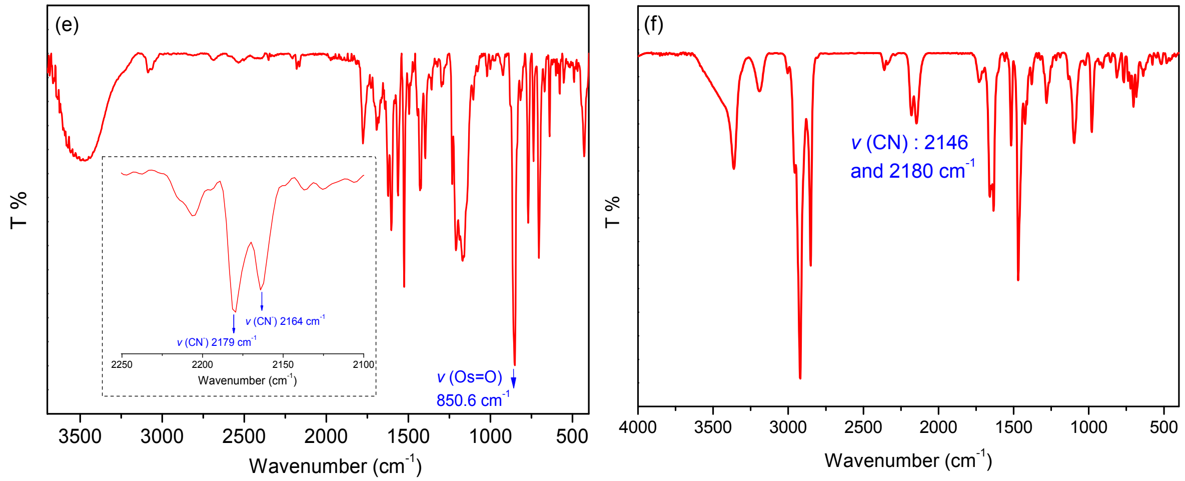

Figure S6. IR spectra of (a) **OsO_2**, (b) **$OsO_2/2B(C_6F_5)_3$**, (c) **OsO_2/Zn**, (d) **OsO_2/TFA**, (e) **$OsO_2/2TFA$** and (f) **$Os^{II}(MeCN)_2/2B(C_6F_5)_3$**.

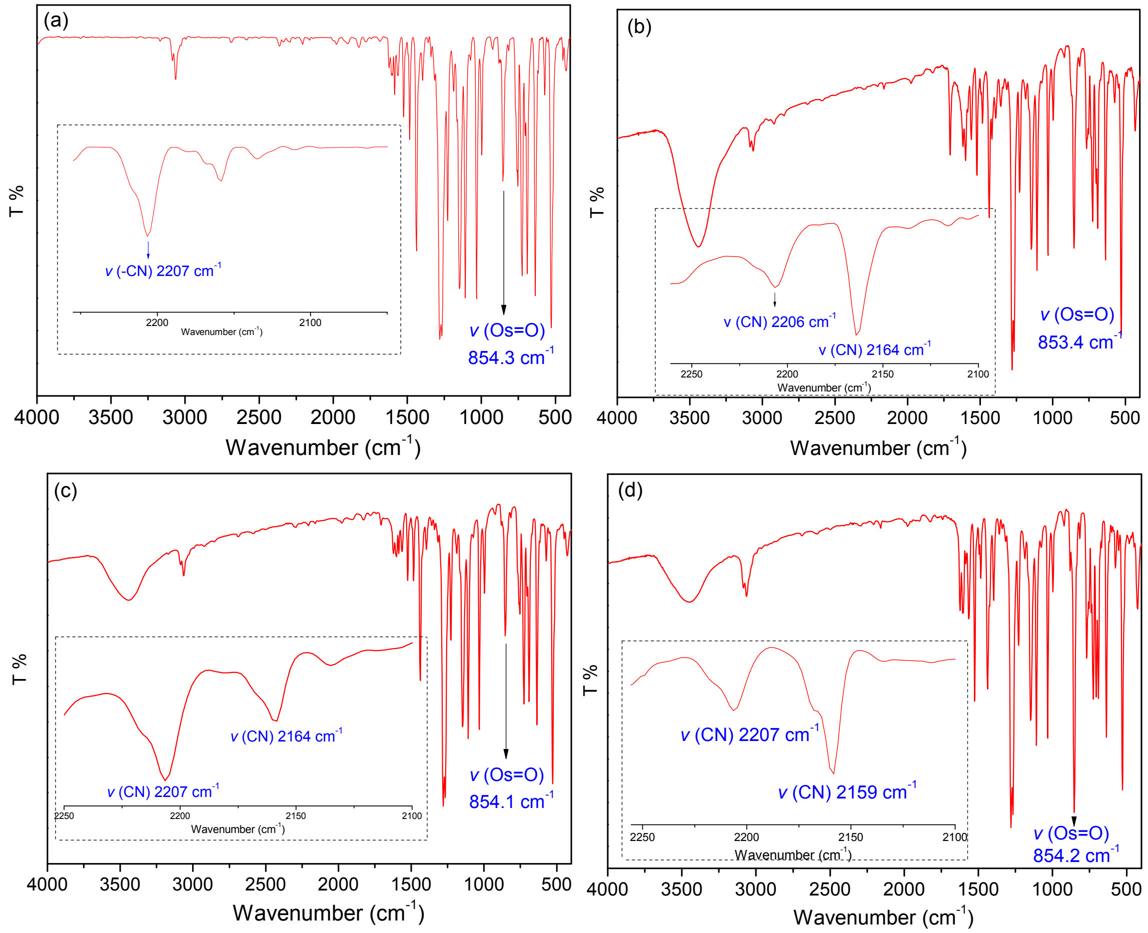

Figure S7. IR spectra of adducts of **OsO_2** with (a) $Sc(OTf)_3$, (b) $Mg(OTf)_2$, (c) $Lu(OTf)_3$, and (d) $Zn(OTf)_2$.

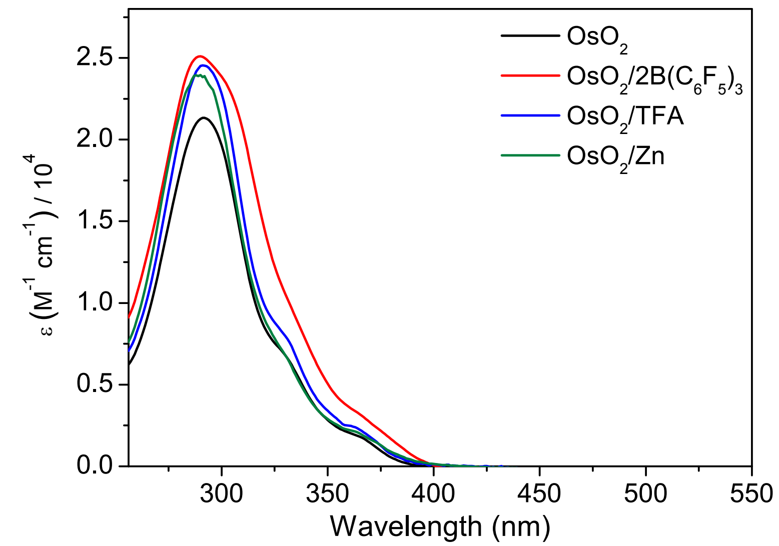

Figure S8. UV-vis spectra of **OsO_2**, **$OsO_2/2B(C_6F_5)_3$**, **OsO_2/TFA** and **OsO_2/Zn** in CH_2Cl_2.

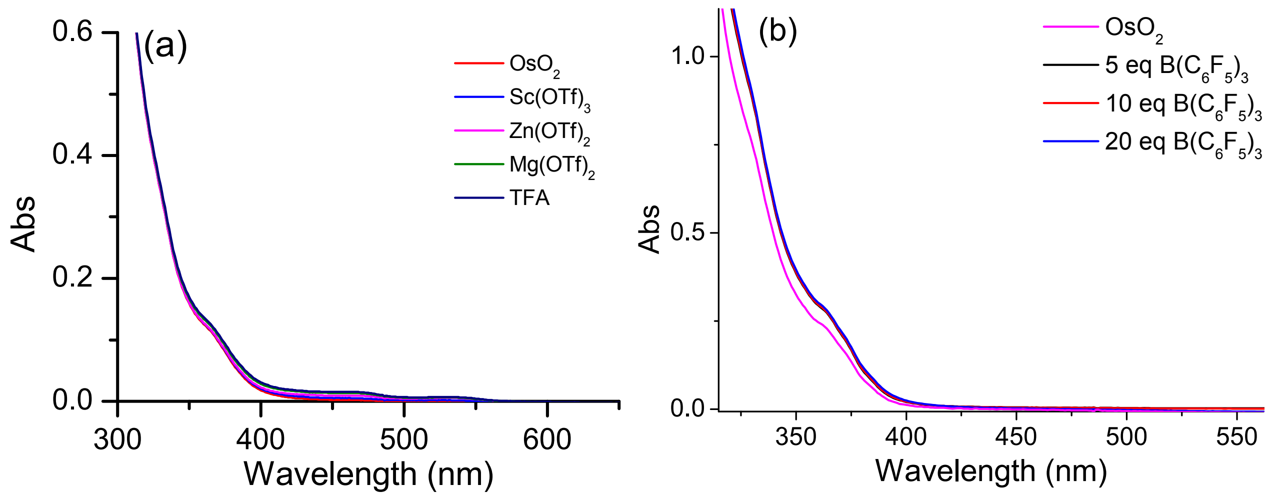

Figure S9. (a) UV-vis spectra of **OsO_2** with 10 equiv. of LAs or TFA in MeCN. (b) UV/vis spectral change of **OsO_2** with addition of various amounts of $B(C_6F_5)_3$ in MeCN at 298 K.

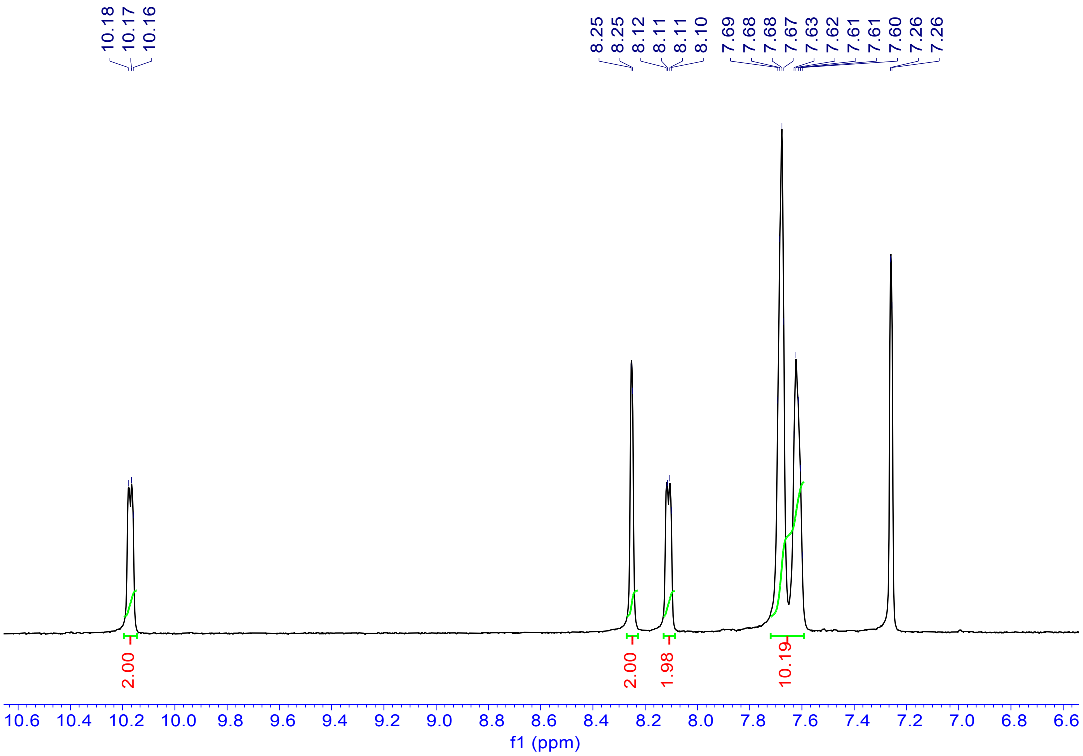

Figure S10. ^{1}H NMR (400 MHz) of **OsO_2** in $CDCl_3$.

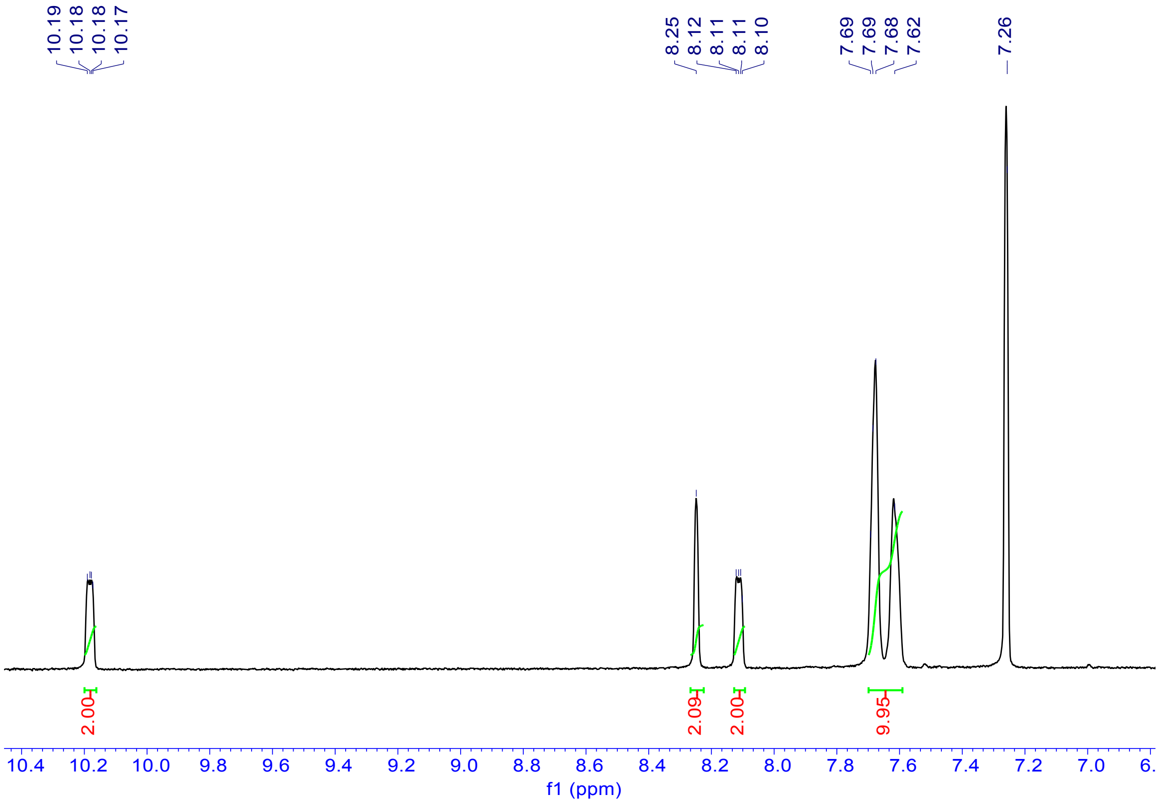

Figure S11. ^{1}H NMR (400 MHz) of **OsO_2/Zn** in $CDCl_3$.

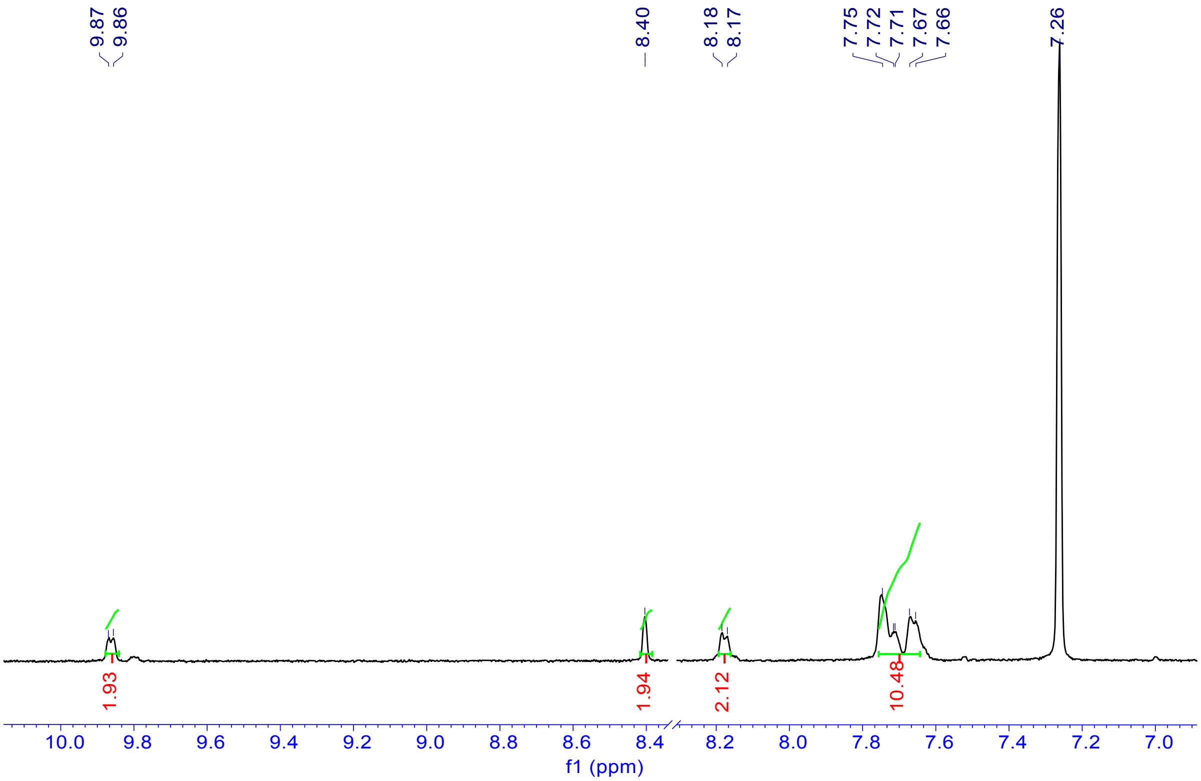

Figure S12. ^{1}H NMR (400 MHz) of **OsO_2/TFA** in $CDCl_3$.

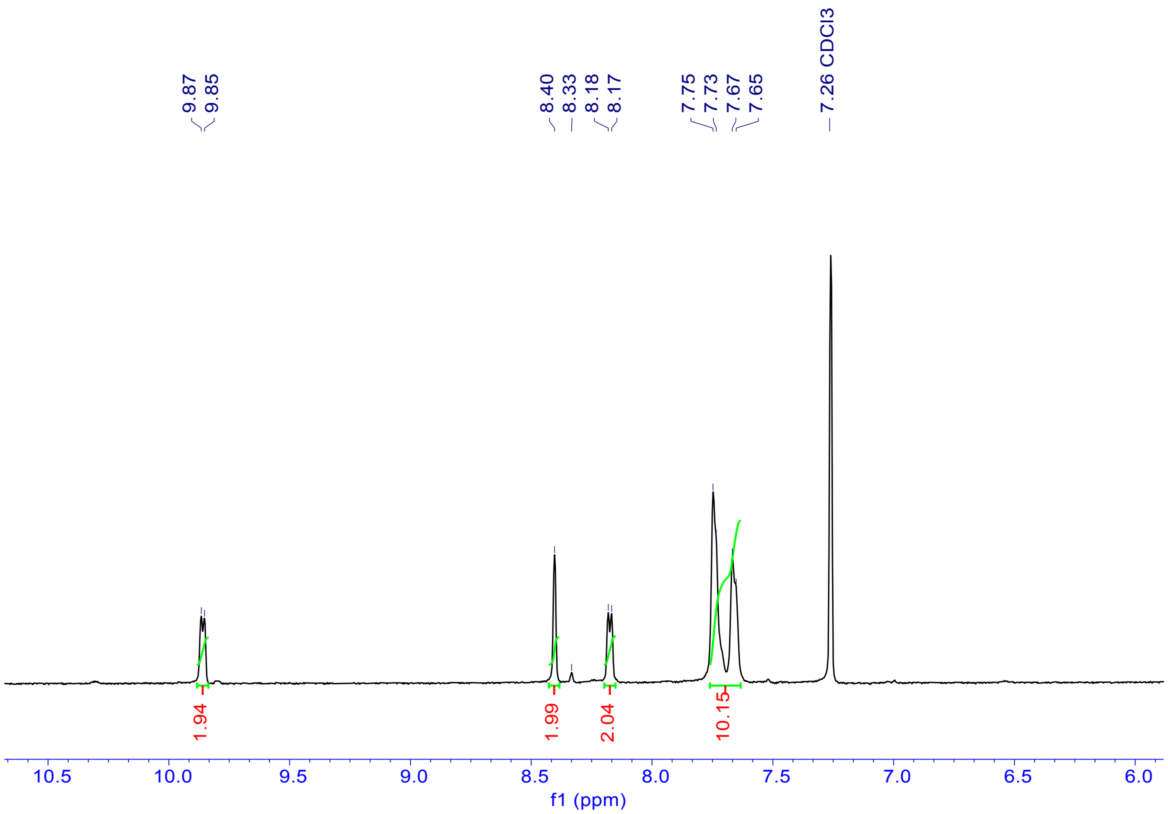

Figure S13. ^{1}H NMR (400 MHz) of **OsO_2/2B(C_6F_5)$_3$** in $CDCl_3$.

Table S2. Selected bond parameters (Å, °) of the complexes.

	$\mathbf{OsO_2}$	$\mathbf{OsO_2/2B(C_6F_5)_3}$	$\mathbf{OsO_2/Zn}$	$\mathbf{OsO_2/TFA}$	$\mathbf{Os^{II}(MeCN)_2/2B(C_6F_5)_3}$
Os1-O1	1.750(3)	1.742(2)	1.750(3)	1.742(2)	/
Os1-O2	1.747(4)	1.767(2)	1.745(3)	1.741(2)	/
Os1-N3	2.134(3)	2.109(2)	2.135(3)	2.141(2)	2.103(2)
Os1-N4	2.158(2)	2.110(3)	2.122(3)	2.151(2)	2.102(2)
Os1-C1	2.044(4)	2.039(3)	2.056(4)	2.056(3)	1.956(3)
Os1-C2	2.040(2)	2.049(3)	2.046(5)	2.057(3)	1.966(3)
C1-N1	1.133(3)	1.144(4)	1.148(5)	1.135(5)	1.151(4)
C2-N2	1.151(3)	1.127(4)	1.145(6)	1.138(4)	1.143(4)
N1-B1	/	1.584(4)	/	/	1.560(4)
N2-B2	/	1.593(4)	/	/	1.546(4)
Os1-C1-N1	176.8(3)	170.2(3)	175.5(4)	176.9(3)	177.2(3)
C1-N1-B1	/	164.5(3)	/	/	167.1(3)
Os1-C2-N2	177.9(2)	175.8(3)	176.9(4)	177.9(3)	176.9(3)
C2-N2-B2	/	178.5(3)	/	/	173.7(3)
N1-Zn1	/	/	2.023(3)	/	/
C1-N1-Zn1	/	/	176.1(3)	/	
d(N2···O3)	/	/	/	2.699	/

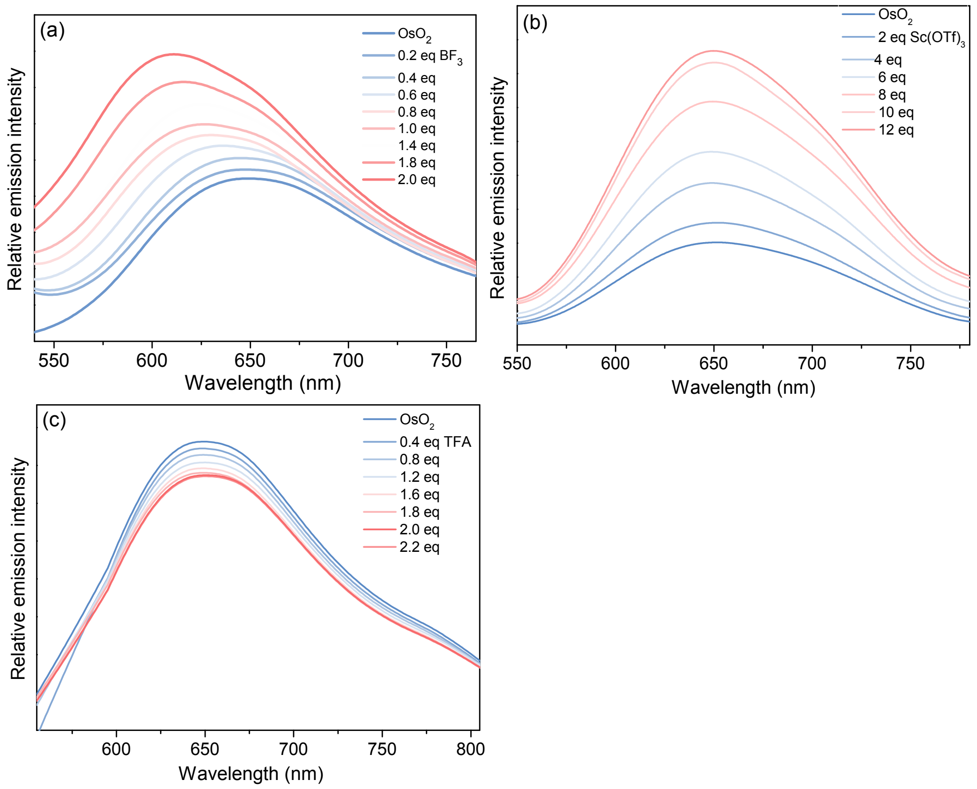

Figure S14. (a) The emission spectral change of $\mathbf{OsO_2}$ by addition of various amounts of BF_3 in MeCN solution at 298 K. (b) The emission spectral change of $\mathbf{OsO_2}$ by addition of various amounts of $Sc(OTf)_3$ in MeCN solution at 298 K. (c) The emission spectral change of $\mathbf{OsO_2}$ by addition of various amounts of

TFA in MeCN solution at 298 K. Excitation with λ_{ex} = 380 nm.

Kinetic studies of the ground state reactivity of $OsO_2/2Sc(OTf)_3$ with substrates:

Reaction of **$OsO_2/2Sc(OTf)_3$** with bromoferrocene: $[Os^{VI}(O)_2(CN)_2(dpphen)(Sc^{III})_2(CF_3SO_3)_6] + 4FcBr \rightarrow [Os^{II}(MeCN)_2(dpphen)(CN)_2(Sc^{III})_2(CF_3SO_3)_6] + 4FcBr^+ + 2H_2O$

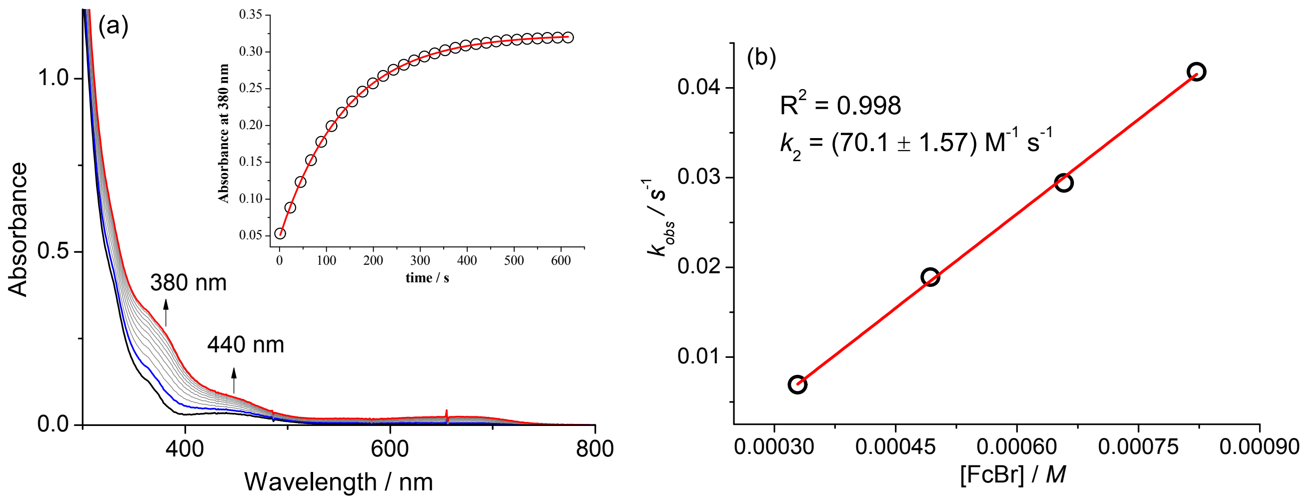

Figure S15. (a) UV-vis spectral changes for the reaction of **OsO_2** (3.29×10^{-5} M) with bromoferrocene (3.29×10^{-4} M) in the presence of $Sc(OTf)_3$ (3.29×10^{-4} M) at 298 K. The inset shows the time course monitored at 380 nm. (b) Plots of the k_{obs} *vs.* the concentration of bromoferrocene to determine the second-order rate constant.

Reaction of **$OsO_2/2Sc(OTf)_3$** with PPh_3: $[Os^{VI}(O)_2(dpphen)(CN)_2(Sc^{III})_2(CF_3SO_3)_6] + 2PPh_3 \rightarrow [Os^{II}(CH_3CN)_2(dpphen)(CN)_2(Sc^{III})_2(CF_3SO_3)_6] + 2OPPh_3$

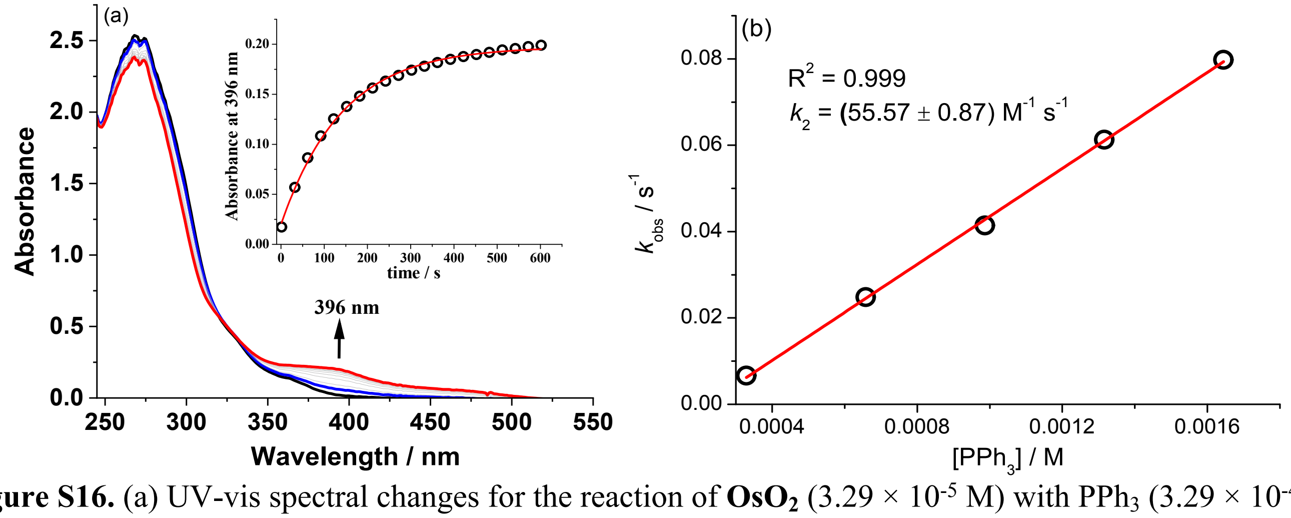

Figure S16. (a) UV-vis spectral changes for the reaction of **OsO_2** (3.29×10^{-5} M) with PPh_3 (3.29×10^{-4} M) in the presence of $Sc(OTf)_3$ (3.29×10^{-4} M) at 298 K. The inset shows the time course monitored at 396 nm. (b) Plot of the k_{obs} against the concentration of PPh_3 to determine the second-order rate constant.

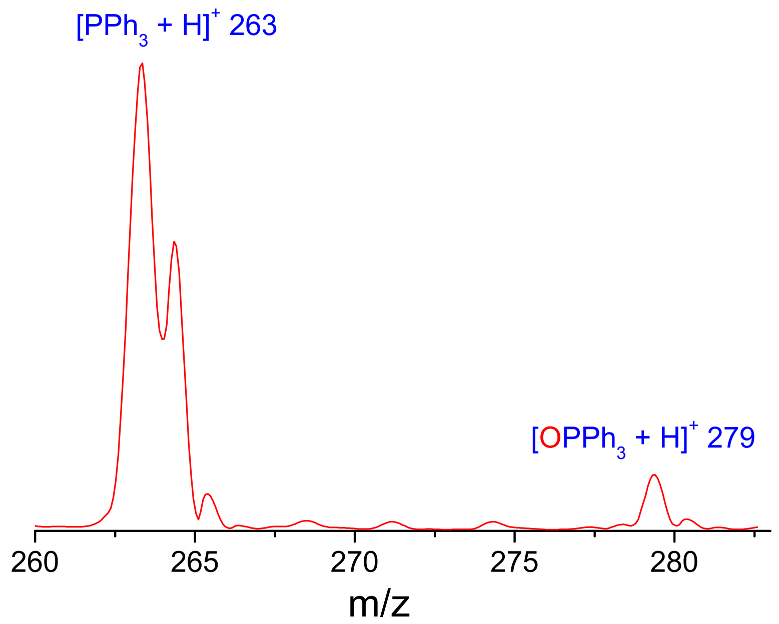

Figure S17. ESI-MS (+ve mode) for the reaction of **OsO_2** (6.5×10^{-5} M) with 100 eq. PPh_3 in the presence of 10 equiv. of $Sc(OTf)_3$ (6.5×10^{-4} M) in MeCN at 298 K.

Reaction of **OsO_2/2$Sc(OTf)_3$** with hydroquinone: $[Os^{VI}(O)_2(dpphen)(CN)_2(Sc^{III})_2(CF_3SO_3)_6] + H_2Q \rightarrow [Os^{II}(MeCN)_2(dpphen)(CN)_2(Sc^{III})_2(CF_3SO_3)_6] + 2Q + 2H_2O$

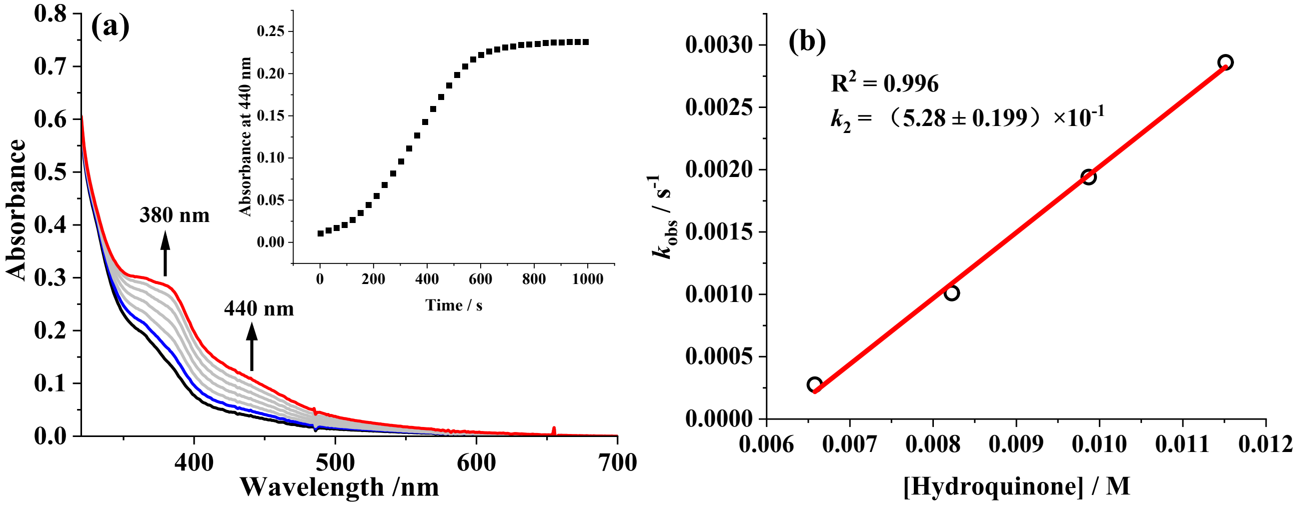

Figure S18. (a) UV-vis spectral changes of the reaction of **OsO_2** (3.29×10^{-5} M) with hydroquinone (6.58×10^{-3} M) in the presence of $Sc(OTf)_3$ (3.29×10^{-4} M) at 298 K. The inset shows the time course monitored at 440 nm. (b) Plot of the k_{obs} against the concentration of hydroquinone to determine the second-order rate constant.

KIE experiment of hydroquinone.

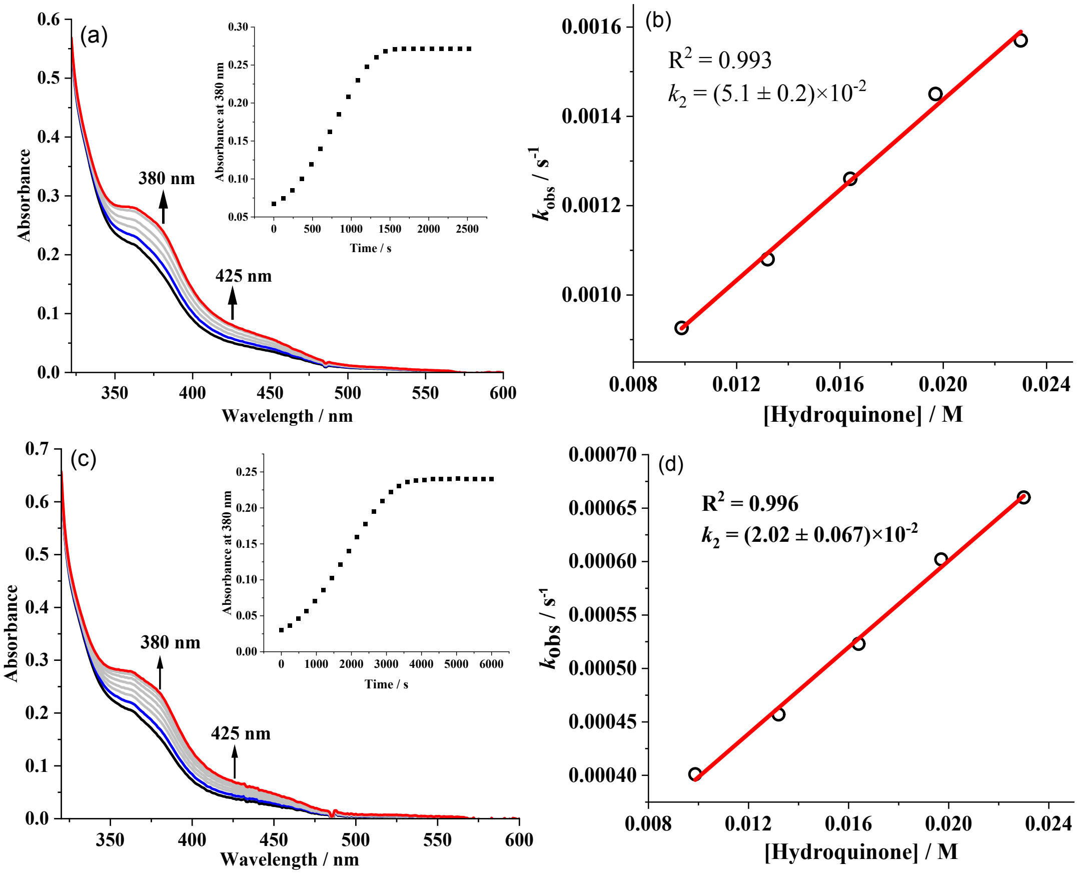

Figure S19. (a) UV-vis spectral changes for the reaction of $\mathbf{OsO_2}$ (3.29×10^{-5} M) with hydroquinone (1.15×10^{-2} M) in the presence of $Sc(OTf)_3$ (3.29×10^{-4} M) in $MeCN/H_2O$ (v/v = 18:1) or (c) $MeCN/D_2O$ (v/v = 18:1) at 298 K. The inset shows the time course monitored at 380 nm. Plots of the k_{obs} against the concentration of (b) hydroquinone or (d) deuterated hydroquinone to determine the second-order rate constant. KIE = 2.7 ± 0.1.

Reaction of $\mathbf{OsO_2/2Sc(OTf)_3}$ with xanthene:

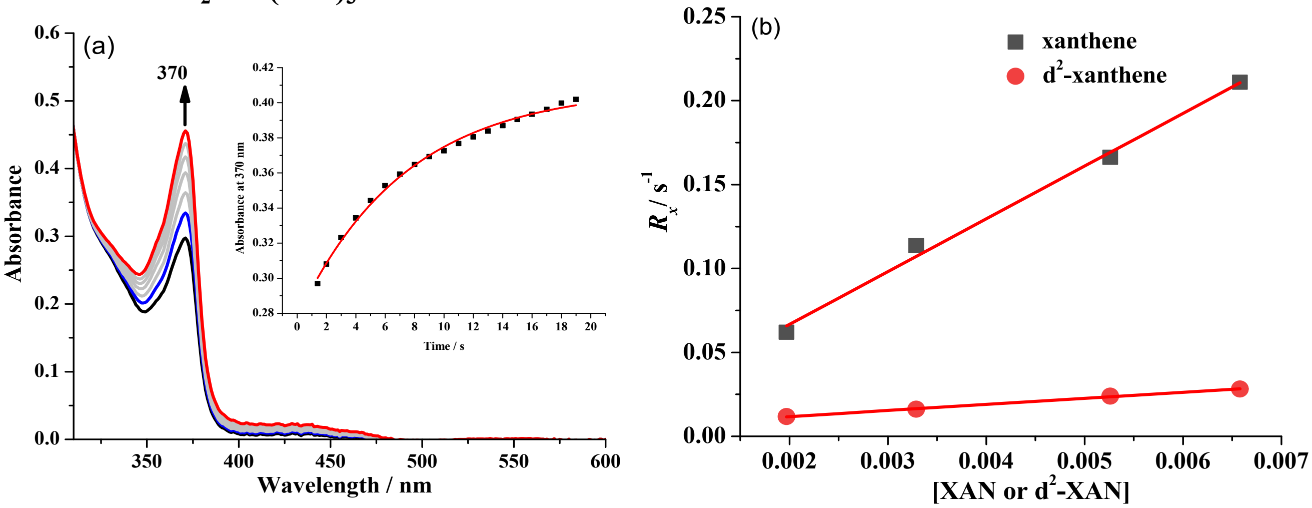

Figure S20. (a) UV-vis spectral changes for the reaction of $\mathbf{OsO_2}$ (3.29×10^{-5} M) with xanthene (9.87×10^{-4} M) in the presence of $Sc(OTf)_3$ (3.29×10^{-4} M). The inset shows the time course monitored at 370 nm. (b) Plots of the initial rate R_x against the concentration of xanthene or d^2-xanthene, and the observed KIE is k(H) / k(D) = 8.7 ± 0.1.

Reaction of $\mathbf{OsO_2/Sc(OTf)_3}$ with 9,10-dihydroanthracene:

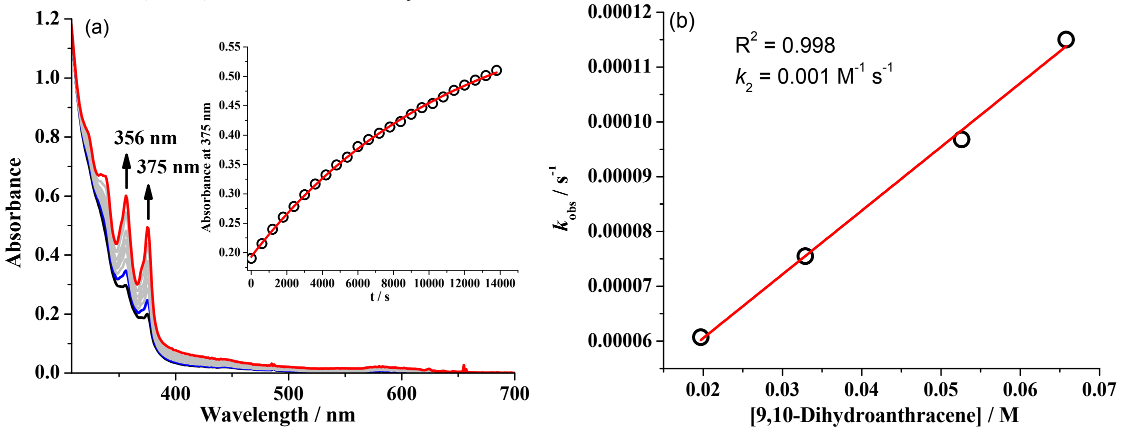

Figure S21. (a) UV-vis spectral changes for the reaction of $\mathbf{OsO_2}$ (3.29×10^{-5} M) with 9,10-dihydroanthracene (3.29×10^{-2} M) in the presence of $Sc(OTf)_3$ (3.29×10^{-4} M). The inset shows the time course monitored at 375 nm. (b) Plots of the k_{obs} against the concentration of 9,10-dihydroanthracene to determine the second-order rate constant.

Estimation of excited state redox potentials of $\mathbf{OsO_2}$ and $\mathbf{OsO_2/2LA}$.

Based on emission and CV data of these complexes, the excited-state reduction potentials were estimated from equation (1), where $E^0(Os^{VI\text{-}V})$ is the redox potential for the reactions (2) and $E_{0\text{–}0}$ refers to the 0-0 emission energy. (ref. 11)

$$E^0(Os^{VI*/V}) = E^0\ (Os^{VI/V}) + E_{0\text{–}0}\ (1)$$

$$Os^{VI}O_2/2LA + e^- \rightarrow Os^{V}O_2/2LA\ (2)$$

From the emission spectra at 298K in MeCN (Figure 4), the $E_{0\text{–}0}$ value for $\mathbf{OsO_2}$ and $\mathbf{OsO_2/2(ScOTf)_3}$ are calculated to be 2.25 and 2.29 eV, respectively, and their reduction potential (equation 2) is -0.04 and 1.21 V vs. NHE, respectively, from electrochemical data. Using equation 1, the excited redox potential of $\mathbf{OsO_2}$ and $\mathbf{OsO_2/2LA}$ were estimated to be 2.21 and 3.50 V vs. NHE, respectively. However, it should be noted that this is simply an estimate and not a quantitative determination.

Table S3. Photocatalytic oxidation of cyclohexane by $\mathbf{OsO_2}/H_2O_2$ in the presence of 10 equiv. of $Sc(OTf)_3$.

Entry	substrate	oxidant	LA	Products	TON
1	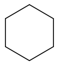	air	/	Cy-OH (1.1mM) /Cy=O (1.3mM)	4.6
2	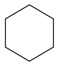	air	$Sc(OTf)_3$	Cy-OH/Cy=O	trace
3	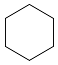	H_2O_2	/	Cy-OH (1.7mM) /Cy=O (2.3mM)	7.8
4	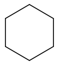	H_2O_2	$Sc(OTf)_3$	Cy-OH (12.4mM) /Cy=O (21.2mM)	68

Reaction conditions: $\mathbf{OsO_2}$ (0.8 mM), cyclohexane (500 μL), $Sc(OTf)_3$ (10 equiv.), H_2O_2 (80 mM). TON based on $\mathbf{OsO_2^*/2Sc(OTf)_3}$ functioning as a two-electron oxidant.

Table S4. Photocatalytic oxidation of benzene to phenol by **OsO_2**/H_2O_2 in the presence of different amounts of $Sc(OTf)_3$. [a]

Entry	Equiv. of $Sc(OTf)_3$	Yield (%) [b]
1	0	0
2	2	33
3	5	58
4	8	63
5	10	71
6	15	61
7	20	42
8	30	21

[a] Reaction conditions: **OsO_2** (0.8 mM), benzene (500 μL), H_2O_2 (80 mM) was slowly added within 4 h. All reactions were carried out in CF_3CH_2OH (1mL) under argon, irradiation time = 10 h with $\lambda > 400$ nm. [b] Yield based on H_2O_2.

Table S5. Photocatalytic oxidation of benzene to phenol by **OsO_2**/H_2O_2 in the presence of various LAs. [a]

Entry	LAs	Yield (%) [b]
1	/	/
2	$Sc(OTf)_3$	71
3	$Lu(OTf)_3$	41
4	$Zn(OTf)_2$	37
5	BF_3	56
6	TFAA	7

[a] Reaction conditions: **OsO_2** (0.8 mM), benzene (500 μL), H_2O_2 (80 mM) (slow addition within 4 h), LAs (8 mM). All reactions were carried out in CF_3CH_2OH (1 mL) under argon, irradiation time = 10 h with $\lambda > 400$ nm.

Table S6. Competitive oxidation of an equimolar mixture of cyclohexane and benzene by **OsO_2/2$Sc(OTf)_3$**/H_2O_2 [a]

Products Selectivity				Total yield based on H_2O_2
Oxidation of benzene		Oxidation of cyclohexane		
Phenol derivatives	benzoquinone derivatives	Cy-OH	Cy=O	
95.1%	0.6%	2.1%	2.2%	65%

[a] Reaction conditions: **OsO_2** (0.8 mM), benzene (300 mM), cyclohexane (300 mM), H_2O_2 (80 mM), $Sc(OTf)_3$ (8 mM). All reactions were carried out in CF_3CH_2OH under argon. Reaction time = 10 h.

Table S7. Photocatalytic oxidation of benzene by **OsO_2/2Sc(OTf)$_3$**/H_2O_2. [a]

Entry	LA	phenol	quinone	Yield [b]	TON	Time
1	Sc(OTf)$_3$	99.6%	<0.2 %	32.7%	2620	24 h
2[c]	Sc(OTf)$_3$	99%	<1 %	51.3%	8660	72 h

[a] Reaction conditions: **OsO_2** (0.01 mM), Sc(OTf)$_3$ (0.1 mM), benzene (500 μL), H_2O_2 (0.08 M, 8000 eq) (slow addition within 8 h). Reaction was carried out in CF_3CH_2OH under argon upon irradiation at λ > 400 nm. [b] Yield was based on the amount of H_2O_2, assuming the system acts as a two-electron oxidant. [c] 3 Å molecular sieves were added at the time of 24 h and 48 h. An additional portion of H_2O_2 (0.08 M) was slowly added within 8 h after a 24 h reaction.

Table S8. Competitive oxidation of an equimolar mixture of ethylbenzene and benzene by **OsO_2/2Sc(OTf)$_3$**/H_2O_2 [a]

Products				Total yield based on H_2O_2
Oxidation of ethylbenzene		Oxidation of benzene		
Phenol derivatives	benzoquinone derivatives	phenol	benzoquinone	
92.4%	0.7%	6.9%	0%	54%

[a] Reaction conditions: **OsO_2** (0.8 mM), benzene (300 mM), ethylbenzene (300 mM), H_2O_2 (80 mM), Sc(OTf)$_3$ (8 mM). All reactions were carried out in CF_3CH_2OH under argon. Reaction time = 10 h.

Table S9. Competitive oxidation of an equimolar mixture of anisole and benzene by **OsO_2/2Sc(OTf)$_3$**/H_2O_2 [a]

Products				Total yield based on H_2O_2
Oxidation of anisole		Oxidation of benzene		
Phenol derivatives	benzoquinones derivatives	phenol	benzoquinone	
98.2%	0.8%	1%	0%	40%

[a] Reaction conditions: **OsO_2** (0.8 mM), benzene (300 mM), anisole (300 mM), H_2O_2 (80 mM), Sc(OTf)$_3$ (8 mM). All reactions were carried out in CF_3CH_2OH under argon. Reaction time = 10 h.

Table S10. Competitive oxidation of an equimolar mixture of nitrobenzene and benzene by $\mathbf{OsO_2/2Sc(OTf)_3}/H_2O_2$. [a]

Products				
Oxidation of nitrobenzene		Oxidation of benzene		Total yield based on H_2O_2
Phenol derivatives	Benzoquinone derivatives	phenol	benzoquinone	
0.5%	0%	99.4%	0.1%	68%

[a] Reaction conditions: $\mathbf{OsO_2}$ (0.8 mM), benzene (300 mM), nitrobenzene (300 mM), H_2O_2 (80 mM), $Sc(OTf)_3$ (8 mM). All reactions were carried out in CF_3CH_2OH under argon. Reaction time = 10 h.

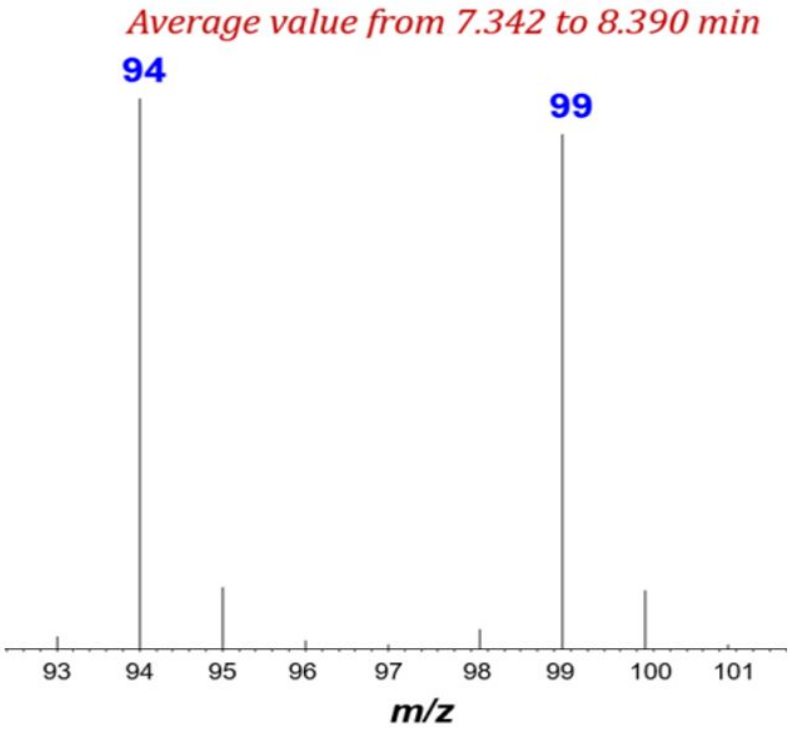

Figure S22. GC/MS of phenol and d^5-phenol obtained from the photocatalytic reaction.

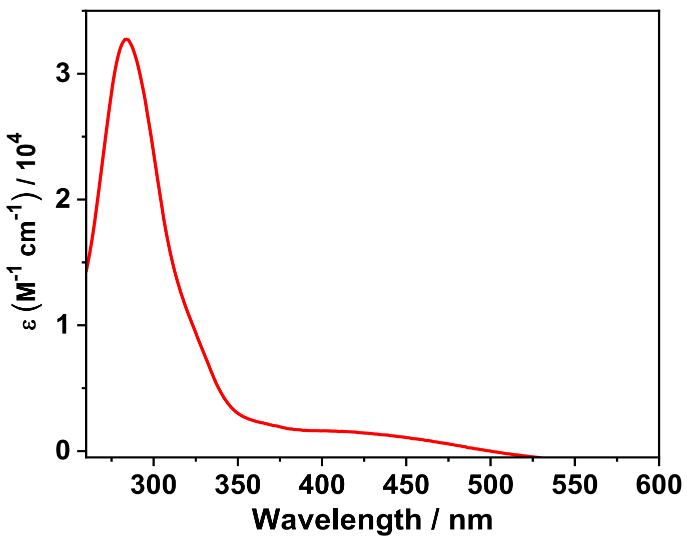

Figure S23. UV-vis spectrum of **$Os^{IV}O/2Sc(OTf)_3$** in MeCN.

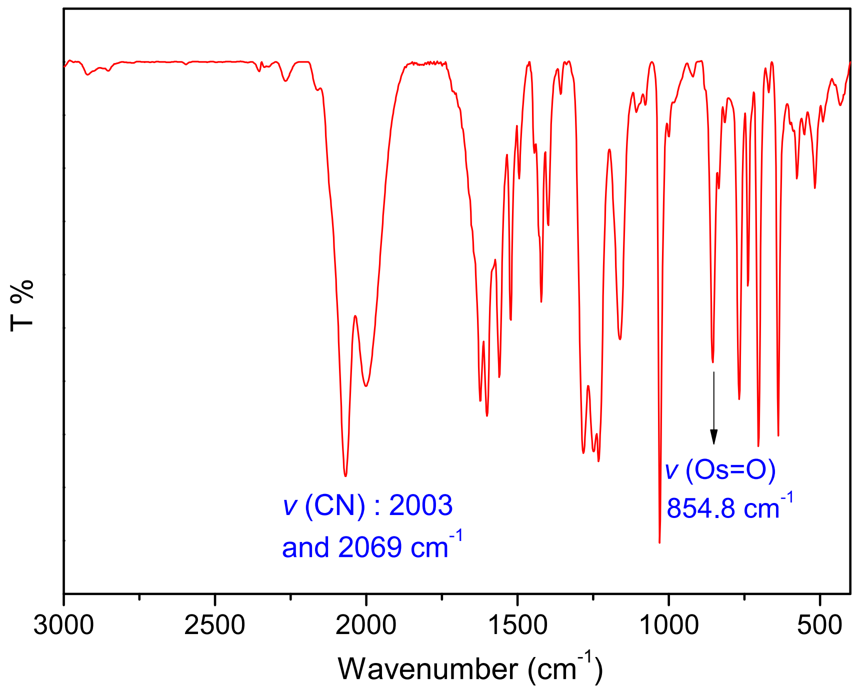

Figure S24. IR spectrum of **$Os^{IV}O/2Sc(OTf)_3$**.

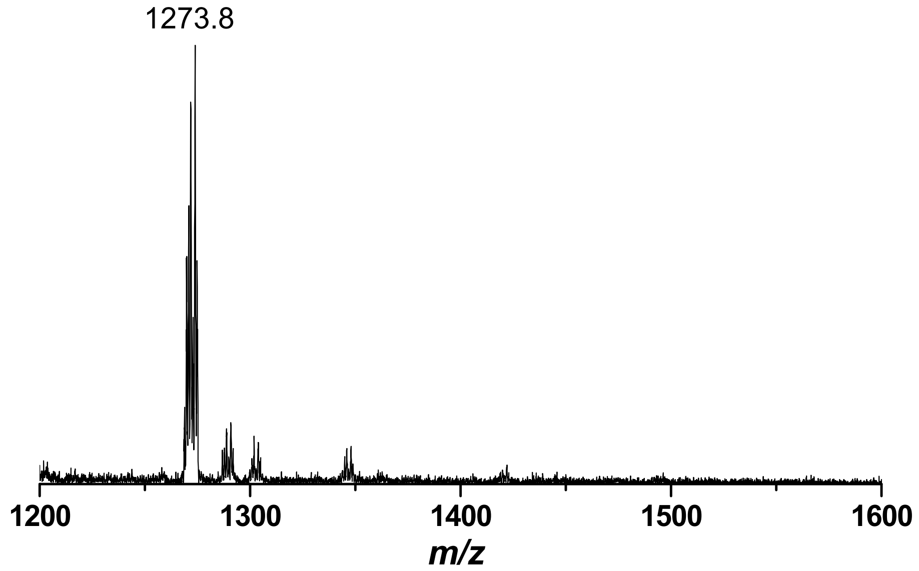

Figure S25. ESI-MS of **$Os^{IV}O/2Sc(OTf)_3$** in CF_3CH_2OH. *m/z* 1273.8, assigned to $[Os^{IV}(O)(MeCN)(dpphen)(CN)_2(Sc^{III})(CF_3SO_3)_4]^-$.

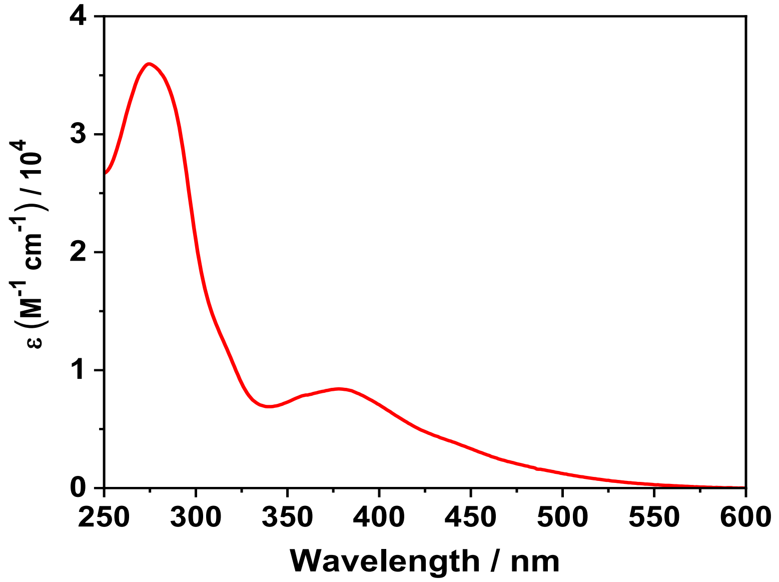

Figure S26. UV-vis spectrum of **$Os^{II}(MeCN)_2/2B(C_6F_5)_3$** in MeCN.

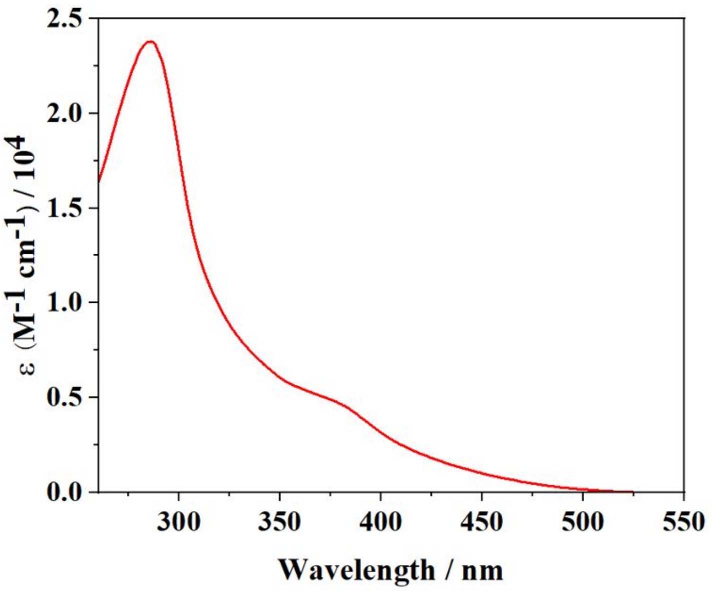

Figure S27. UV-vis spectrum of **$Os^{II}(MeCN)_2/2Sc(OTf)_3$** in MeCN.

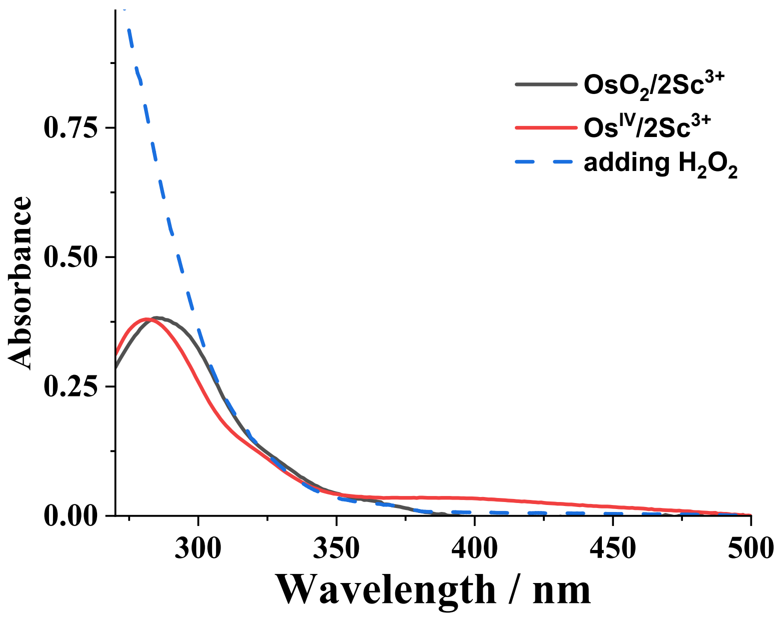

Figure S28. The UV/vis spectra of **$OsO_2/2Sc(OTf)_3$**, **$Os^{IV}O/2Sc(OTf)_3$**, and the addition of H_2O_2 into a CH_3CN solution of **$Os^{IV}O/2Sc(OTf)_3$**, leading to a rapid change in the UV/vis spectrum.

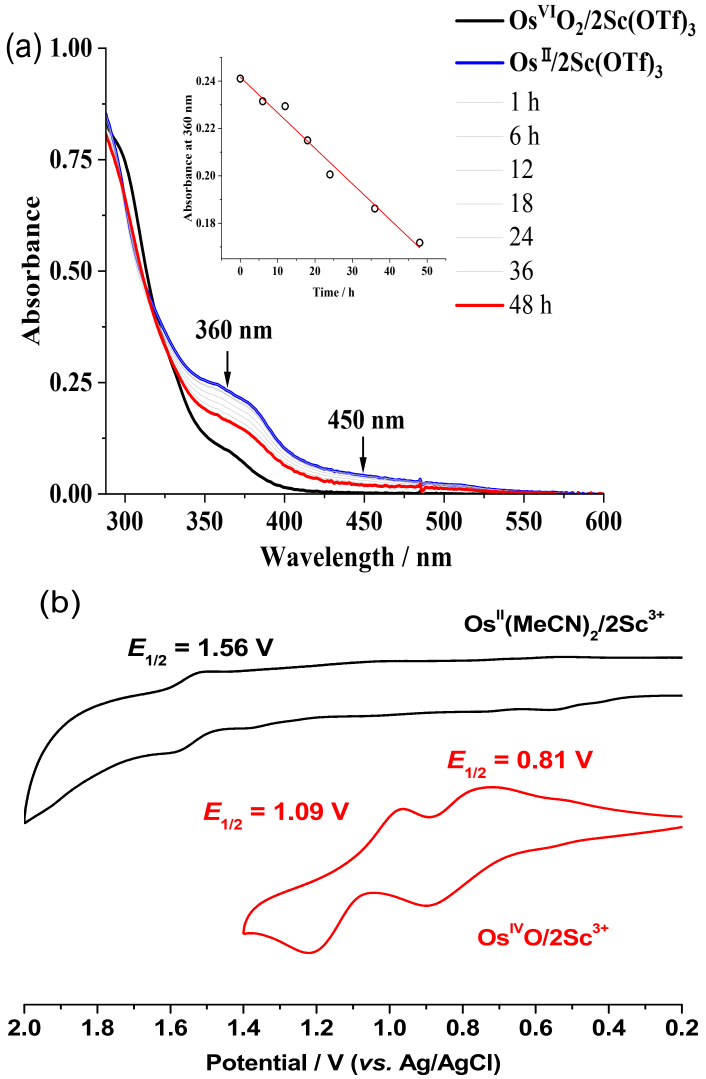

Figure S29. (a) The UV/vis spectral change for the reaction of **$Os^{II}(MeCN)_2/2Sc(OTf)_3$** ($2.38 \times 10^{-5}$ M) with H_2O_2 (2.38×10^{-4} M) at different time intervals at room temperature (inset shows the absorbance change at 360 nm at different time intervals). (b) CV of **$Os^{II}(MeCN)_2/2Sc(OTf)_3$** and **$Os^{IV}O/2Sc(OTf)_3$** in 0. 1 *M* [^{n}Bu$_4$N]PF$_6$ MeCN solution. (scan rate = 100 mV/s)

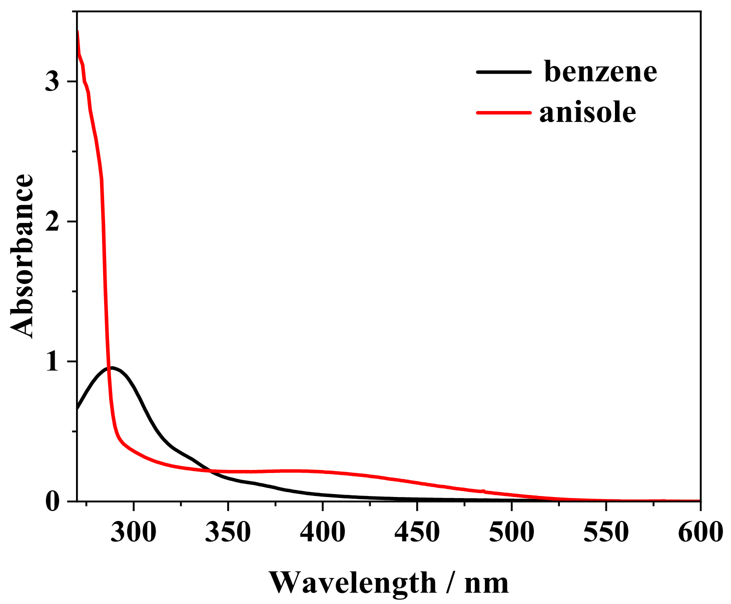

Figure S30. UV/vis spectra after photocatalytic oxidation of anisole and benzene in MeCN (after removal of solvent and redissolved in MeCN).

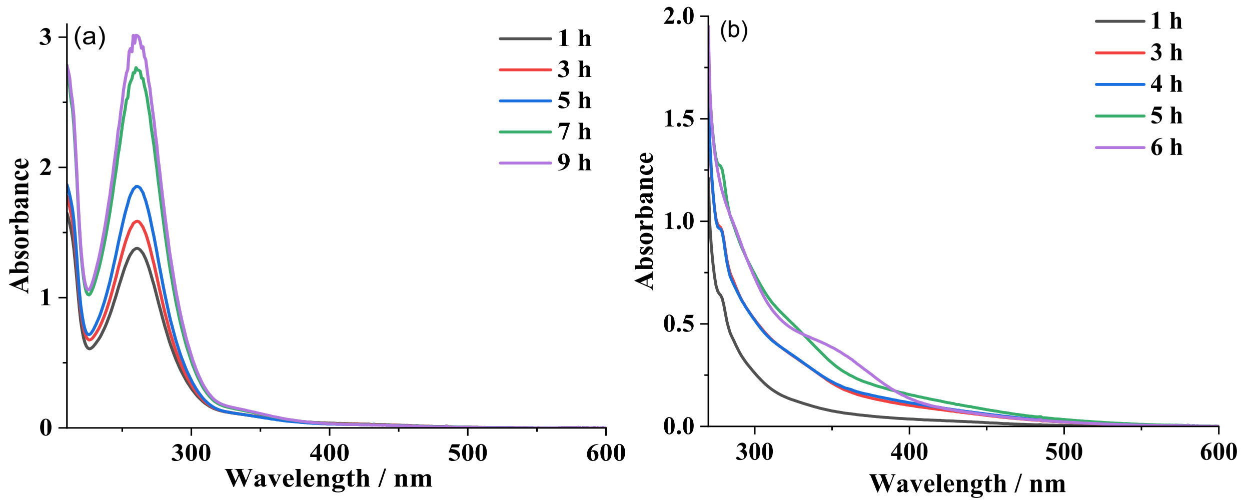

Figure S31. UV/vis spectral changes for the photoreaction of $\mathbf{OsO_2/2Sc(OTf)_3}$ with nitrobenzene (a) and ethylbenzene (b) in MeCN.

Theoretical method.

The structures of molecular species related to **OsO_2** were optimized at the B3LYP-D3(BJ)/def2-TZVP level.[1-4] For larger systems involving **OsO_2/2Sc(OTf)$_3$**, a composite approach was adopted: geometry optimizations were conducted at the B3LYP-D3(BJ)/def2-SVP level, followed by single-point energy calculations at the B3LYP-D3(BJ)/def2-TZVP level (denoted as B3LYP-D3(BJ)/def2-TZVP//B3LYP-D3(BJ)/def2-SVP). The polarizable continuum model (PCM) was used to account for the solvent effect in dichloromethane.[5,6] All calculations were executed using the Gaussian 16 Rev. C.01 program.[7] To model emission properties in acetonitrile, selected intermediates were re-optimized at the B3LYP-D3(BJ)/def2-SVP (PCM, acetonitrile) level. Subsequently, time-dependent (TD)-DFT calculations at the same level were performed to obtain the vertical energies.[8,9] The natural transition orbital (NTO) analysis was employed to investigate the $S_0 \rightarrow T_1$ transition. [10]

Reference

1. A. D. Becke. J. Chem. Phys. 1993, **98**, 5648-5652.

2. S. Grimme; S. Ehrlich; L. Goerigk. J. Comput. Chem. 2011, **32**, 1456-1465.

3. D. Andrae; U. Häußermann; M. Dolg; H. Stoll; H. Preuß. Theor. Chim. Acta 1990, **77**, 123-141.

4. F. Weigend; R. Ahlrichs. Phys. Chem. Chem. Phys. 2005, **7**, 3297-3305.

5. S. Miertuš; E. Scrocco; J. Tomasi. Chem. Phy. 1981, **55**, 117-129.

6. S. Miertuš̃; J. Tomasi. Chem. Phy. 1982, **65**, 239-245.

7. Gaussian 16 Rev. C.01, M. J. Frisch; G. W. Trucks; H. B. Schlegel; G. E. Scuseria; M. A. Robb; J. R. Cheeseman; G. Scalmani; V. Barone; G. A. Petersson; H. Nakatsuji; X. Li; M. Caricato; A. V. Marenich; J. Bloino; B. G. Janesko; R. Gomperts; B. Mennucci; H. P. Hratchian; J. V. Ortiz; A. F. Izmaylov; J. L. Sonnenberg; Williams; F. Ding; F. Lipparini; F. Egidi; J. Goings; B. Peng; A. Petrone; T. Henderson; D. Ranasinghe; V. G. Zakrzewski; J. Gao; N. Rega; G. Zheng; W. Liang; M. Hada; M. Ehara; K. Toyota; R. Fukuda; J. Hasegawa; M. Ishida; T. Nakajima; Y. Honda; O. Kitao; H. Nakai; T. Vreven; K. Throssell; J. A. Montgomery Jr.; J. E. Peralta; F. Ogliaro; M. J. Bearpark; J. J. Heyd; E. N. Brothers; K. N. Kudin; V. N. Staroverov; T. A. Keith; R. Kobayashi; J. Normand; K. Raghavachari; A. P. Rendell; J. C. Burant; S. S. Iyengar; J. Tomasi; M. Cossi; J. M. Millam; M. Klene; C. Adamo; R. Cammi; J. W. Ochterski; R. L. Martin; K. Morokuma; O. Farkas; J. B. Foresman; D. J. Fox, Gausssian, Inc., Wallingford, CT, 2016.

8. C. Adamo; D. Jacquemin. Chem. Soc. Rev. 2013, **42**, 845-856.

9. A. D. Laurent; C Adamo; D. Jacquemin. Phys. Chem. Chem. Phys. 2014, **16**, 14334-14356.

10. R. L. Martin. J. Chem. Phys. 2003, **118**, 4775-4777.

11. Che, C.-M., Lam, M. H.-W. & Mak, T. C. W. J. Chem. Soc. Chem. Commun. 1989, **20**, 1529-1531.

Table S11. Optimized Os=O and Os-CN bond lengths (in Å) and ∠NC-Os-CN angles (°) calculated at the B3LYP-D3(BJ)/def2-SVP level with PCM (dichloromethane).

	Os=O		Os-CN		∠NC-Os-CN	
	S_0	T_1	S_0	T_1	S_0	T_1
**OsO_2**	1.739/1.739	1.808/1.808	2.044/2.044	2.040/2.026	89.9	84.9
**$OsO_2/2BF_3$**	1.740/1.740	1.903/1.786	2.062/2.062	2.071/2.021	93.9	95.1
**$OsO_2/2Sc(OTf)_3$** [a]	1.741/1.741	1.907/1.783	2.069/2.069	2.074/2.019	90.5	91.5
**$OsO_2/2Sc(OTf)_3$** [b]	1.742/1.740	1.926/1.773	2.075/2.051	2.075/2.017	83.7	84.4
**$OsO_2/2Sc(OTf)_3$** [c]	1.743/1.737	1.948/1.765	2.057/2.055	2.044/2.015	82.2	81.4
**$OsO_2/2Sc(OTf)_3$** [d]	1.741/1.739	1.938/1.768	2.061/2.056	2.065/2.010	84.3	83.5

[a] Both Sc metals are four-coordinate.

[b] One Sc is four-coordinate, and the other one is five-coordinate, with one oxygen atom from the OTf^- ligands coordinating to the adjacent Sc^{3+} ion.

[c] Both Sc metals are five-coordinate.

[d] One Sc is five-coordinate, and the other is six-coordinate, see the structure in Table S16.

Table S12. The selected orbital energies (E, in Hartrees) and energy splitting (in eV) of the optimized S_0 structures calculated at the B3LYP-D3(BJ)/def2-SVP level with PCM (dichloromethane).

	$E[p_y(O^{2-})]$	$E[p_x(O^{2-})]$	$E(p_x)–E(p_y)$	$E[d_{yz}(Os^{VI})]$	$E[d_{xz}(Os^{VI})]$	$E(d_{xz})–E(d_{yz})$
**OsO_2**	-0.31230	-0.31147	0.023	-0.12575	-0.12590	-0.004
**$OsO_2/2BF_3$**	-0.34258	-0.33867	0.106	-0.15692	-0.15455	0.064
**$OsO_2/2Sc(OTf)_3$***	-0.33845	-0.33326	0.141	-0.15466	-0.15003	0.126

* The most stable **$OsO_2/2Sc(OTf)_3$** complex, see Table S15.

Two non-equivalent Os=O bonds are observed in the optimized **$OsO_2/2Sc(OTf)_3$** (T_1) structure, in contrast to the equivalent Os=O bonds found in the corresponding S_0 structure. This structural distortion likely originates from symmetry breaking of the originally degenerate $d_{\pi*}$ orbitals upon coordination of Lewis acidic moieties, such as BF_3 or $Sc(OTf)_3$, to the terminal nitrogen atoms of the –CN ligands. Orbital energy analysis reveals a progressive lifting of this degeneracy that correlates strongly with the change in the ∠NC–Os–CN angle between the two CN ligands. In bare **OsO_2**, the d_{yz} and d_{xz} orbitals are nearly degenerate, with an energy splitting of only 0.004 eV. This splitting increases to 0.064 eV in **$OsO_2/2BF_3$** and further to 0.126 eV in **$OsO_2/2Sc(OTf)_3$**, where the most stable structure features one five-coordinate and one six-coordinate Sc center. This trend indicates that coordination of Lewis-acidic appendages, particularly $Sc(OTf)_3$, effectively perturbs the degeneracy of the metal-centered $d_{\pi*}$ orbitals in OsO_2. In the most stable **$OsO_2/2Sc(OTf)_3$** (S_0) structure, the ∠NC–Os–CN angle between the two σ(Os–CN) bonds is reduced to 84.3°, compared with ~90° in the pseudo-octahedral OsO_2 complex. The closer spatial proximity of the two σ(Os–CN) bonding interactions causes orbital repulsion, resulting in destabilization (energy increase) of the d_{xz}(Os) orbital and concomitant stabilization (energy lowering) of the d_{yz}(Os) orbital. This perturbation breaks the degeneracy of the d_{xz}(Os) and d_{yz}(Os) orbitals, leading to an observable energy splitting. Upon $S_0 \rightarrow T_1$ excitation, corresponding to an LMCT transition [$p_\pi(O^{2-}) \rightarrow d_{\pi*}(Os^{VI})$], the **$OsO_2/2Sc(OTf)_3$** ($T_1$) state exhibits pronounced Os=O bond asymmetry, with bond lengths of 1.938 Å and 1.768 Å. The elongated Os=O bond is consequently more reactive and is identified as the preferred site for HAT in the oxidation of DHA and cyclohexane. The coordination of LA to -CN makes the Os(VI) center more electron-deficient to enhance the ET reactivity. Upon binding of $Sc(OTf)_3$ to the -CN group, the spin density at the Os(VI) center in **OsO_2** (T_1) decreases from 0.75 to 0.40, while the spin density on the longer oxo ligand increases from 0.57 to 0.96. This redistribution of spin density indicates enhanced polarization of the Os=O bond, making the Os(VI) center more electron-poor and the oxo ligand

more electron-rich. As a result, the Os(VI) center exhibits an increased propensity to undergo reduction, leading to a shift of the reduction potential toward more positive values. In addition, the DFT calculations indicated that binding of $Sc(OTf)_3$ to **OsO_2** (S_0) lowers the LUMO level (i.e., $d_{\pi*}(Os^{VI})$) from ~-0.12 to ~-0.15 Hartrees, enhancing the electron acceptability of the Os(VI) center.

Table S13. The emission energies (λ in nm) for $T_1 \rightarrow S_0$ of **OsO_2**, **OsO_2/BF_3**, **OsO_2/2Sc(OTf)$_3$** calculated at the B3LYP-D3(BJ)/def2-SVP level with PCM (acetonitrile).

	λ_{max} of the T_1 state arising from LMCT $[p_{\pi}(O^{2-}) \rightarrow d_{\pi*}(Os^{VI})]$	λ_{max} from the triplet state arising from LMCT $[\pi(phen) \rightarrow d_{yz}(Os^{VI})]$ [e]	λ_{max}
**OsO_2**	648	/	650
**OsO_2/2BF_3**	723	614	608
**OsO_2/2Sc(OTf)$_3$** [a]	733	612	650
**OsO_2/2Sc(OTf)$_3$** [b]	744	622	
**OsO_2/2Sc(OTf)$_3$** [c]	730	592	
**OsO_2/2Sc(OTf)$_3$** [d]	737	605	

[a] Both Sc metals are four-coordinate.

[b] One Sc is four-coordinate, and the other one is five-coordinate, with one oxygen atom from the OTf^- ligands coordinating to the adjacent Sc^{3+} ion.

[c] Both Sc metals are five-coordinate.

[d] One Sc is five-coordinate, and the other is six-coordinate, see the structure in Table S16.

[e] The LMCT $[\pi(phen) \rightarrow d_{xz}(Os^{VI})]$ is higher than LMCT $[\pi(phen) \rightarrow d_{yz}(Os^{VI})]$ by 0.08 eV.

Table S14. Orbital contributions in the $S_0 \rightarrow T_1$ transition, from NTO analysis at the optimized S_0 structures, at the TD-B3LYP-D3(BJ)/def2-SVP level with PCM (acetonitrile).

	$p_y(O^{2-}) \rightarrow d_{yz}(Os^{VI})$	$p_x(O^{2-}) \rightarrow d_{xz}(Os^{VI})$
**OsO_2**	53.0%	46.3%
**OsO_2/2BF_3**	55.9%	43.4%
**OsO_2/2Sc(OTf)$_3$**	60.0%	39.3%

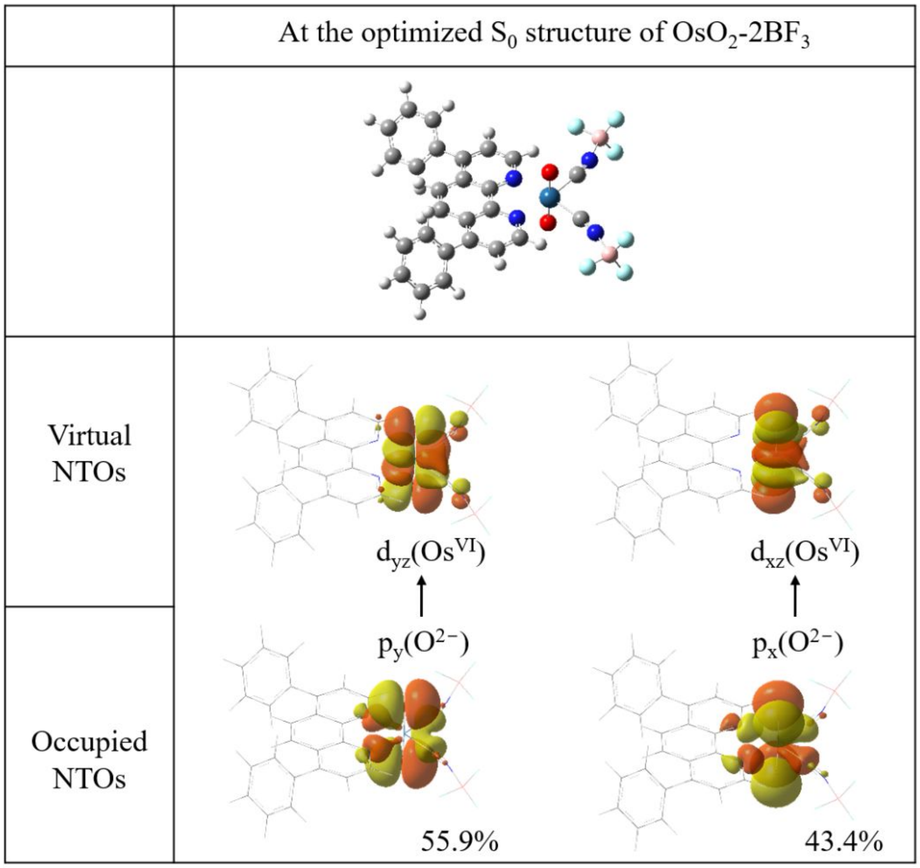

Figure S32. NTO (natural transition orbital) analysis of LMCT [$p_{\pi}(O^{2-}) \rightarrow d_{\pi *}(Os^{VI})$] transition at the optimized S_0 structure of **OsO_2/2BF_3** complex.

	At the optimized S_0 structure of OsO_2-$2BF_3$
	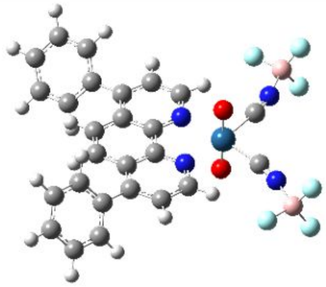
Virtual NTOs	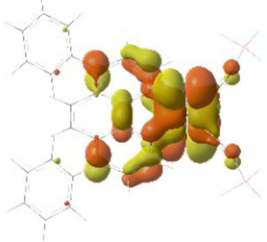 $d_{yz}(Os^{VI})$ ↑
Occupied NTOs	π(Phen) 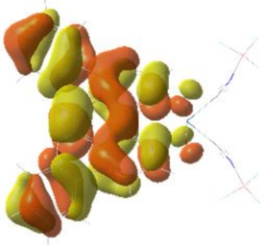 97.8%

Figure S33. NTO analysis of LMCT [π(phen)→$d_{yz}(Os^{VI})$] transition at the optimized S_0 structure of **OsO_2/$2BF_3$** complex.

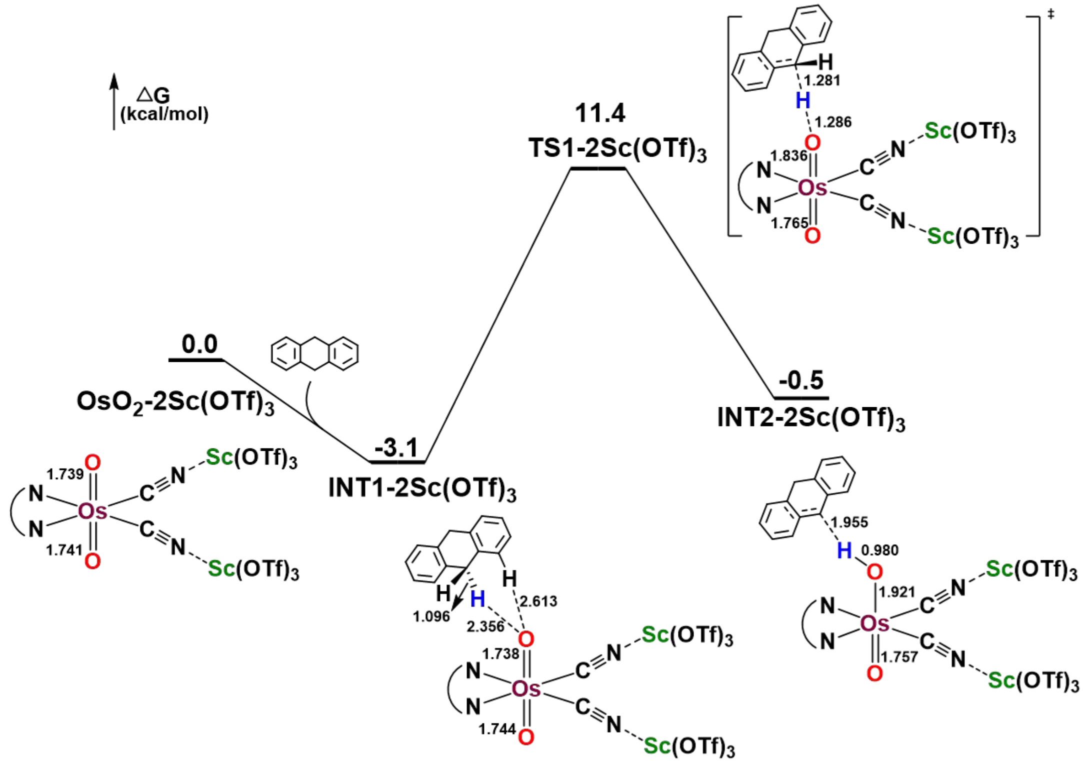

Figure S34. PES profile for the oxidation of DHA by **$OsO_2/2Sc(OTf)_3$** (S_0). The second HAT pathway is omitted, as it is expected to follow a mechanism similar to that shown in Figure 9.

Table S15. Comparison of the Gibbs free energy for different coordination modes of two OTf^- ligands in **$OsO_2/2Sc(OTf)_3$** calculated at the B3LYP-D3(BJ)/def2-SVP level with PCM (dichloromethane).

	$\Delta G_{298}^{\ddagger}$ (kcal mol^{-1}, singlet)	$\Delta G_{298}^{\ddagger}$ (kcal mol^{-1}, triplet)
**$OsO_2/2Sc(OTf)_3$** [a]	0.0	0.0
**$OsO_2/2Sc(OTf)_3$** [b]	-18.3	-17.2
**$OsO_2/2Sc(OTf)_3$** [c]	-37.6	-37.9
**$OsO_2/2Sc(OTf)_3$** [d]	-45.2	-45.1
**$OsO_2/2Sc(OTf)_3$** [e]	-22.3	Not available

[a] Both Sc metals are four-coordinate.
[b] One Sc is four-coordinate, and the other one is five-coordinate, with one oxygen atom from the OTf^- ligands coordinating to the adjacent Sc^{3+} ion.
[c] Both Sc metals are five-coordinate.
[d] One Sc is five-coordinate, and the other is six-coordinate, see the structure in Table S16.
[e] Both Sc metals are six-coordinate.

Table S16. Optimized structures of $\mathbf{OsO_2}$ and the most stable $\mathbf{OsO_2/2Sc(OTf)_3}$ complex (S_0 and T_1), together with the corresponding TS for the HAT step, calculated at the B3LYP-D3(BJ)/def2-TZVP (for $\mathbf{OsO_2}$ systems) and B3LYP-D3(BJ)/def2-TZVP//B3LYP-D3(BJ)/def2-SVP (for $\mathbf{OsO_2/2Sc(OTf)_3}$ systems) respectively with PCM (dichloromethane).

	**Structure**	**HAT**<br>**HOMO energy (a.u.)**	**TS**<br>$\mathbf{\Delta G_{298}^{\ddagger}}$ **(kcal/mol)**
$\mathbf{OsO_2}$<br>$\mathbf{(S_0)}$	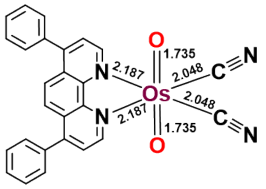	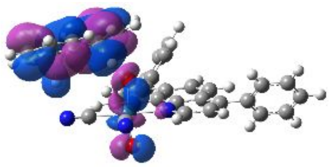<br>**-0.20665**	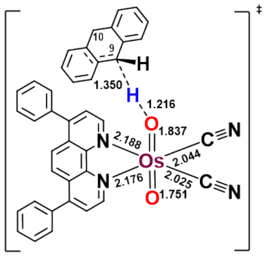<br>**18.5**
$\mathbf{OsO_2}$**-**<br>$\mathbf{2Sc(OTf)_3}$<br>$\mathbf{(S_0)}$	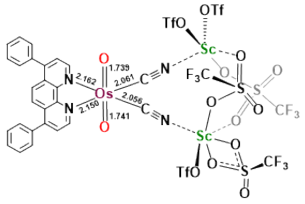	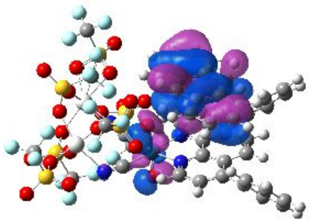<br>**-0.22800**	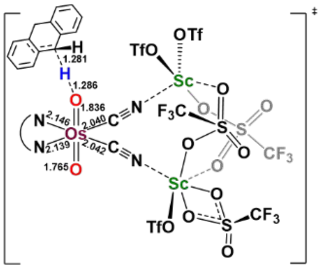<br>**11.4**
$\mathbf{^3OsO_2}$<br>$\mathbf{(T_1)}$	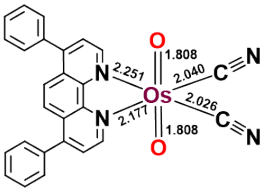	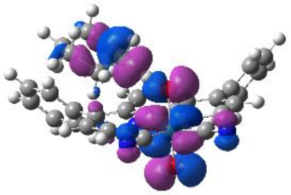<br>**-0.22374**	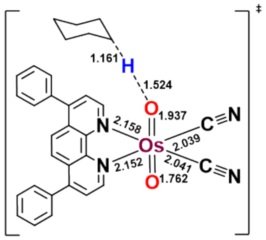<br>**7.3**
$\mathbf{^3OsO_2}$**-**<br>$\mathbf{2Sc(OTf)_3}$<br>$\mathbf{(T_1)}$	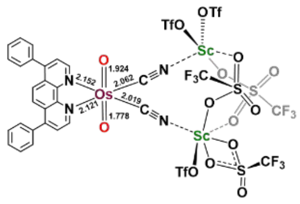	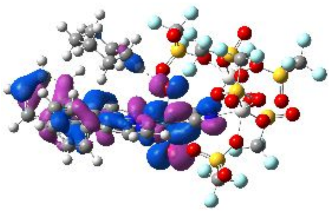<br>**-0.25553**	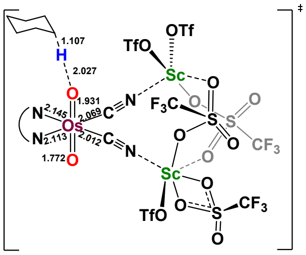

			**5.3**

The Cartesian coordinates of transition states and intermediates.

OsO_2

Os	-2.66779900	0.00073600	0.00000400
C	-4.12120500	-1.44162700	0.04998100
N	-4.89879500	-2.29553200	0.08123100
C	0.16459600	-3.44772900	0.07447900
C	1.40957100	-2.84926800	0.00406000
C	1.46590900	-1.42666600	-0.00442700
C	0.24880300	-0.71726500	0.00890500
C	-0.99342300	-2.67480600	0.08829000
C	2.68306900	-0.67920800	-0.00061800
C	0.24922500	0.71739300	-0.00889800
C	1.46682800	1.42593200	0.00429500
C	2.68348500	0.67768500	0.00022800
C	1.41146000	2.84860100	-0.00402300
C	0.16688300	3.44788800	-0.07417300
C	-0.99168800	2.67573400	-0.08793000
H	3.62032700	-1.21315600	0.00875600
H	0.07472000	-4.52384700	0.08383900
H	-1.97225400	-3.13114000	0.12014100
H	3.62107600	1.21105100	-0.00935800
H	0.07766400	4.52406300	-0.08336600
H	-1.97018900	3.13280800	-0.11959100
N	-0.95107000	-1.35336300	0.04248900
N	-0.95024700	1.35430000	-0.04229100
O	-2.51793400	-0.05714000	-1.72789900
O	-2.51814100	0.05861000	1.72792500
C	-4.12073400	1.44368000	-0.05017200
N	-4.89815500	2.29773800	-0.08147700
C	2.62131200	-3.69095100	-0.05889700
C	2.81863500	-4.69518600	0.89248500
C	3.55665500	-3.53254800	-1.08562400
C	3.93871900	-5.51278400	0.82833900
H	2.10186500	-4.82144300	1.69329800
C	4.66771800	-4.36134700	-1.15527000
H	3.39988600	-2.77804200	-1.84467300
C	4.86479200	-5.34877100	-0.19581600
H	4.08728400	-6.27824500	1.57833800
H	5.37786700	-4.23793600	-1.96210300
H	5.73475600	-5.98987600	-0.24838400
C	2.62376100	3.68948000	0.05888000
C	3.55929600	3.53010000	1.08528200
C	2.82148100	4.69392400	-0.89220300
C	4.67088500	4.35819900	1.15495300
H	3.40229900	2.77540200	1.84409100
C	3.94207700	5.51081200	-0.82804000
H	2.10460300	4.82090300	-1.69280400
C	4.86830900	5.34586700	0.19582700
H	5.38116400	4.23403700	1.96155600
H	4.09092700	6.27644100	-1.57781200
H	5.73867000	5.98643000	0.24841100

$OsO_2/2Sc(OTf)_3$

Os	2.01350900	0.65890800	0.23059700
C	0.60472600	2.07288300	0.74342300
N	-0.30138800	2.72724400	1.06156000
C	5.12933500	3.80912100	0.63847900
C	6.32093300	3.11235300	0.44752300
C	6.23611900	1.72095500	0.11632200
C	4.95060200	1.13122300	0.06502900
C	3.89715100	3.15115400	0.56789200
C	7.36733200	0.89284600	-0.18788900
C	4.80121700	-0.26337600	-0.25129000
C	5.93367000	-1.05651800	-0.55019700
C	7.22370700	-0.43038000	-0.49929800
C	5.71945500	-2.44058100	-0.85712800
C	4.41617800	-2.92695600	-0.78626800
C	3.34687300	-2.07620300	-0.48679700
H	8.36126100	1.33613000	-0.17457900
H	5.14421000	4.87008800	0.88707500
H	2.96202400	3.68439300	0.74373500
H	8.10728800	-1.03064200	-0.70807500
H	4.19879200	-3.97335800	-0.99977700
H	2.32850800	-2.46581900	-0.45144700
N	3.81607700	1.84949200	0.30800000
N	3.53868400	-0.78199300	-0.24312300
O	2.27603200	0.28928900	1.91178500
O	1.91333600	1.05397300	-1.45993600
C	0.39790600	-0.60638700	0.10205000
N	-0.53099700	-1.29296100	0.03098500
C	7.61277600	3.81926300	0.59203000
C	7.81877100	5.04126900	-0.07275400
C	8.62480700	3.31645800	1.43042700
C	9.02016000	5.73414200	0.08117800
H	7.04113200	5.43706100	-0.72932400
C	9.81875300	4.02019100	1.59245900
H	8.46370300	2.38838300	1.98196100
C	10.02194800	5.22605500	0.91399900
H	9.17408900	6.67484700	-0.45170600
H	10.59160600	3.62699600	2.25631300
H	10.95975100	5.77203100	1.03789500
C	6.82296800	-3.35085200	-1.23508000
C	7.71598200	-3.01886800	-2.27047900
C	6.95607300	-4.59233700	-0.58812100
C	8.72475400	-3.90837500	-2.64216400
H	7.60032000	-2.07585000	-2.80778500
C	7.97525400	-5.47253100	-0.95371600
H	6.26968400	-4.85735700	0.21876100
C	8.86125200	-5.13301400	-1.98088400
H	9.40370100	-3.64550600	-3.45614200
H	8.07729100	-6.42762100	-0.43412800
H	9.65552400	-5.82492300	-2.26957400
Sc	-2.27764700	-2.60469200	0.27512800
O	-1.00542700	-3.82559600	1.23016200
O	-3.32576400	-1.19908300	-0.87858200

O	-3.53228500	-4.20531500	-0.42580100
S	-2.74918200	-4.50039100	-1.70192600
S	-3.38914100	0.17797300	-1.47170500
S	0.32268300	-4.59220000	1.05955900
O	-4.70659400	0.61502000	-1.88275400
O	-2.62191700	1.13559400	-0.58951400
O	0.16017800	-6.00725700	1.36487700
O	1.04030300	-4.19577000	-0.15424600
O	-2.36029800	-5.87394000	-1.94547300
O	-1.66738200	-3.44205500	-1.68834300
C	1.30364500	-3.86726100	2.47914100
C	-3.91106400	-4.02160800	-3.09659500
C	-2.29129500	0.06560800	-2.99766000
F	2.38422500	-4.61462900	2.67836100
F	1.68918100	-2.62559400	2.17995900
F	0.57424300	-3.84117600	3.58722700
F	-4.57242500	-2.91824700	-2.78804800
F	-4.76500500	-5.01487800	-3.28955300
F	-3.19256300	-3.82536900	-4.19207600
F	-1.02092100	0.04241000	-2.63936300
F	-2.59792900	-1.05372200	-3.63912300
F	-2.53191400	1.10482800	-3.77314900
Sc	-2.50462100	2.49958700	0.90475700
O	-2.12433900	0.85472300	2.18899100
O	-2.45678900	4.01114800	-0.39429400
O	-4.21488100	2.96056200	1.75797500
S	-5.43915900	3.92178900	1.71090500
S	-1.69364400	4.31356100	-1.70890300
S	-2.68418500	-0.34446000	2.89833900
O	-1.04265300	5.61667400	-1.65527900
O	-0.94786900	3.14678600	-2.18180800
O	-3.87739800	-0.13258400	3.69221600
O	-2.75395500	-1.52731200	1.96358600
O	-5.04336600	5.27082800	1.32407000
O	-6.29908100	3.70493200	2.86631800
C	-1.27870800	-0.81736800	4.03834400
C	-6.33535600	3.19169200	0.24019200
C	-3.15062100	4.49867600	-2.86232900
F	-1.55054800	-1.98814200	4.59008600
F	-0.15668600	-0.89814200	3.33802900
F	-1.15877400	0.11034900	4.97408700
F	-6.58324800	1.90381900	0.45069700
F	-5.58428300	3.32208100	-0.84874800
F	-7.48103400	3.83967200	0.06485000
F	-3.82973000	3.35704800	-2.92000700
F	-3.95293400	5.46615200	-2.43583200
F	-2.70203000	4.80161800	-4.07696300

INT1

Os	-0.59886200	-2.07150500	-1.43813300
C	-0.53012400	-4.11930800	-1.47293400
N	-0.43900200	-5.27098800	-1.47001000
C	3.48769700	-2.38051700	0.32700800
C	3.88593800	-1.06274800	0.45923900

C	2.95788800	-0.04575000	0.09711000
C	1.70703500	-0.44367000	-0.41472900
C	2.23415300	-2.69243000	-0.19269900
C	3.21410500	1.35170500	0.23918500
C	0.73516800	0.54100400	-0.79526500
C	1.01611900	1.91307900	-0.63977500
C	2.29412500	2.28249600	-0.11939700
C	0.01961000	2.84907200	-1.04031300
C	-1.14388900	2.35323300	-1.59910100
C	-1.34982700	0.98282100	-1.71100700
H	4.16334100	1.66787900	0.64238800
H	4.15889000	-3.18436800	0.59068900
H	1.92154800	-3.71798700	-0.32624500
H	2.53239300	3.32949800	-0.02032500
H	-1.93322200	3.02239500	-1.90202500
H	-2.26890200	0.58686500	-2.11708400
N	1.37958200	-1.75380300	-0.56452500
N	-0.44317200	0.10448100	-1.31275900
O	0.18587300	-1.91970500	-2.97901500
O	-1.18239400	-2.02179800	0.19434500
C	-2.48001000	-2.12924000	-2.24449900
N	-3.54499600	-2.09690700	-2.69128800
C	5.23985600	-0.75594300	0.96342600
C	5.67710100	-1.31842700	2.16535600
C	6.11369200	0.05466200	0.23318600
C	6.95716900	-1.05997100	2.63663700
H	5.00535200	-1.94367400	2.73877900
C	7.39772700	0.29902200	0.69990700
H	5.79629400	0.47185000	-0.71306600
C	7.82040500	-0.25196700	1.90498300
H	7.27990800	-1.49074900	3.57514900
H	8.06959800	0.91736000	0.11960300
H	8.81968200	-0.05527100	2.27019900
C	0.17618200	4.30680900	-0.87551600
C	0.55846400	4.86407200	0.34833700
C	-0.13051900	5.15927000	-1.93963400
C	0.63016400	6.24061100	0.50285600
H	0.76246900	4.22063400	1.19216800
C	-0.04523200	6.53624400	-1.78713600
H	-0.42144300	4.73930000	-2.89338200
C	0.33278200	7.08079500	-0.56497300
H	0.91120700	6.65757600	1.46080700
H	-0.27509800	7.18342100	-2.62308900
H	0.39251200	8.15428000	-0.44392200
H	-0.98342200	-0.02936300	1.69973600
C	-4.34041600	-2.60293900	3.84260300
C	-4.58811600	-1.95798100	2.63602400
C	-3.72875300	-0.96761000	2.16950300
C	-2.60883000	-0.61476900	2.92839000
C	-2.37001400	-1.25651600	4.13989900
C	-3.22752000	-2.25054300	4.59798100
C	-2.43217900	1.53229300	1.66810800
C	-3.54522900	1.17642200	0.89854700
C	-4.24001000	2.16338900	0.20473100

H	-5.10385600	1.88703600	-0.38807700
C	-3.84175200	3.49434200	0.27103200
C	-2.74369200	3.84842400	1.04551100
C	-2.04764100	2.86704800	1.74112400
H	-5.01610700	-3.37244300	4.19339200
H	-5.45796500	-2.22656700	2.04835600
H	-1.50539700	-0.97481200	4.72957900
H	-3.03086000	-2.74285300	5.54180500
H	-4.39052800	4.25111400	-0.27467200
H	-2.42549000	4.88079800	1.10259800
H	-1.19336500	3.13920700	2.34902100
C	-1.68191600	0.44836800	2.39771600
H	-1.08000400	0.87301200	3.20164200
C	-3.96507700	-0.27036000	0.85400400
H	-5.01047200	-0.35466000	0.55640200
H	-3.38607900	-0.78946100	0.08257100

INT1-2Sc(OTf)$_3$

Os	2.04285100	-0.62639700	-0.75232700
C	1.09348000	-2.44136000	-1.06698400
N	0.36147400	-3.34327200	-1.13840700
C	5.93003100	-2.75852700	-0.50365500
C	6.85921500	-1.72169700	-0.46121200
C	6.36515400	-0.37965700	-0.41249600
C	4.96515500	-0.19396400	-0.46289600
C	4.55837800	-2.48957600	-0.57279900
C	7.19068600	0.78427100	-0.27143600
C	4.40174700	1.12300000	-0.36667700
C	5.23496200	2.25210300	-0.19656300
C	6.65312000	2.03640100	-0.17400400
C	4.60515600	3.53562400	-0.09665600
C	3.22421800	3.59509500	-0.26698100
C	2.46748000	2.43315400	-0.43023700
H	8.27057800	0.65971200	-0.22403000
H	6.26227000	-3.79636600	-0.51751300
H	3.82086500	-3.29148900	-0.61474800
H	7.31387400	2.89595500	-0.08158700
H	2.69575200	4.54374200	-0.21550400
H	1.38485800	2.49415200	-0.53561800
N	4.09927500	-1.24176900	-0.58755400
N	3.04498900	1.23314300	-0.45785500
O	2.23746600	-0.42511000	-2.47417500
O	1.89940500	-0.85704200	0.96411300
C	0.13192000	0.12420400	-0.72785500
N	-0.95200900	0.50288300	-0.58582900
C	8.30574500	-2.03106300	-0.42477900
C	8.79597900	-2.95348000	0.51634400
C	9.20004900	-1.44274300	-1.33723600
C	10.15605600	-3.26458600	0.55725600
H	8.10991500	-3.41253300	1.23097200
C	10.55660100	-1.76787200	-1.30239500
H	8.82673400	-0.75007600	-2.09380200
C	11.03873400	-2.67333000	-0.35167400
H	10.52698800	-3.97164200	1.30227600

H	11.23955600	-1.31448800	-2.02400500
H	12.10214500	-2.92075200	-0.32207200
C	5.35115000	4.77679500	0.19890700
C	6.29599800	4.82416900	1.24091400
C	5.06108100	5.95770400	-0.50817100
C	6.93229300	6.02220600	1.56629800
H	6.50874400	3.92699700	1.82223900
C	5.70979300	7.15111500	-0.19031300
H	4.33526300	5.93503600	-1.32338400
C	6.64447700	7.18774100	0.84933000
H	7.65095500	6.04593200	2.38819600
H	5.48282100	8.05713600	-0.75617200
H	7.14601200	8.12435500	1.10255400
Sc	-3.00430600	1.23349300	-0.45282500
O	-2.39819400	2.62016700	-1.75730500
O	-3.41855000	-0.28474300	0.91743100
O	-4.30925200	2.71088500	0.46657800
S	-3.29448300	3.22402100	1.47704300
S	-3.33945900	-1.35607300	1.96713100
S	-1.31729700	3.65602200	-2.12270200
O	-4.48710400	-1.45696100	2.84345500
O	-2.90355300	-2.65710600	1.34232300
O	-1.91345500	4.86432500	-2.67656200
O	-0.29108900	3.77006300	-1.08444800
O	-3.03116000	4.64889100	1.51152500
O	-2.11850200	2.29018300	1.27930900
C	-0.49486700	2.78376000	-3.56829300
C	-4.02019500	2.76915300	3.14179700
C	-1.85081400	-0.86731400	3.00592900
F	0.14407600	3.68538500	-4.30422100
F	0.38529600	1.88701800	-3.12390900
F	-1.40263800	2.16682400	-4.31601600
F	-4.49767800	1.53359600	3.09955200
F	-4.99675900	3.61591600	3.42482500
F	-3.06785100	2.85539300	4.05749400
F	-0.81369700	-0.65702800	2.20508100
F	-2.12465900	0.24264800	3.67257300
F	-1.59078700	-1.84740200	3.84898300
Sc	-1.48456100	-3.50343000	0.07717600
O	-2.22757900	-2.19990800	-1.35959500
O	-0.23769800	-3.57050900	1.63084400
O	-2.20316800	-5.23963300	-0.47506600
S	-1.71597700	-6.72150700	-0.57064000
S	1.12589300	-3.68893600	2.34837900
S	-3.16442400	-1.56501700	-2.34933200
O	2.24395500	-3.81362600	1.41274600
O	1.21309000	-2.75523500	3.46649300
O	-4.32407500	-2.34686100	-2.72452400
O	-3.45262500	-0.13669200	-1.95500000
O	-0.26411500	-6.80452400	-0.46088900
O	-2.42234200	-7.42171500	-1.63307400
C	-2.06791200	-1.35616300	-3.85297600
C	-2.41574900	-7.36789200	1.04326200
C	0.91822500	-5.37790600	3.11884100

F	-0.95780300	-0.71987100	-3.50532600
F	-1.76844400	-2.55523500	-4.32412000
F	-2.72018400	-0.65594300	-4.76462200
F	-3.73866800	-7.43263700	0.96551100
F	-2.07643400	-6.56371800	2.04851800
F	-1.92366900	-8.57982800	1.26729800
F	-0.15615900	-5.39085600	3.90010800
F	0.78845600	-6.29886100	2.17271400
F	1.99641000	-5.64614500	3.84957700
C	0.67955300	3.80672500	2.16723200
C	0.91888700	2.48043600	2.54203300
C	2.14887600	2.10515100	3.09764600
C	3.16287100	3.07036400	3.24696900
C	2.91872500	4.39614700	2.87089900
C	1.67852400	4.76801300	2.34317300
C	2.42210400	0.69952300	3.58340000
C	4.48392900	2.63304900	3.83750400
C	4.86636400	1.23233500	3.41509200
C	3.84526200	0.26969100	3.30860400
C	4.17207600	-1.05298400	2.98140700
H	3.37782100	-1.79370000	2.90465900
C	5.50285700	-1.41906300	2.76353300
C	6.51631600	-0.46069200	2.85035000
C	6.19428700	0.86107300	3.17186000
H	2.26321300	0.68193200	4.68069500
H	-0.27916300	4.07596700	1.72443100
H	0.13212400	1.73729900	2.40782600
H	3.71052200	5.14140500	2.98374500
H	1.50019800	5.80516800	2.04866200
H	5.27847800	3.35275700	3.59557600
H	5.74403800	-2.45487000	2.51479200
H	7.55557600	-0.73896400	2.65941200
H	6.98531800	1.61281800	3.24178300
H	4.39423900	2.64527300	4.94227900
H	1.71243500	-0.01858600	3.15751200

INT2

Os	-0.82979000	-1.76372600	-1.20722200
C	-1.44858600	-3.70520600	-1.03279200
N	-1.74999100	-4.81558600	-0.90933300
C	3.07566000	-3.30619300	0.23415100
C	3.83432800	-2.16207400	0.40543300
C	3.21711700	-0.90914600	0.13267900
C	1.88972500	-0.91688900	-0.33743000
C	1.76591900	-3.22793800	-0.23210300
C	3.85403900	0.35503900	0.32875400
C	1.22405900	0.31593100	-0.63634900
C	1.87453800	1.54747100	-0.42991200
C	3.21747100	1.52273900	0.05736400
C	1.16021100	2.73744500	-0.74476200
C	-0.10389500	2.60730400	-1.29104700
C	-0.68178700	1.35365200	-1.45398500
H	4.86453200	0.37411700	0.70599000
H	3.50254500	-4.27883300	0.42985100

H	1.17129000	-4.11681400	-0.38528000
H	3.73602300	2.45767700	0.20109000
H	-0.68187300	3.48227100	-1.54364800
H	-1.68555200	1.24131900	-1.83591700
N	1.19264000	-2.07015700	-0.52424000
N	-0.04159900	0.24323100	-1.12247400
O	-0.34872900	-1.88211200	-2.88381500
O	-1.18374500	-1.46079400	0.67594300
C	-2.72872900	-1.22072500	-1.67896900
N	-3.78818900	-0.83469400	-1.94219700
C	5.23550500	-2.27469900	0.85872900
C	5.53661000	-3.01176100	2.00700100
C	6.27787900	-1.69284800	0.13144600
C	6.85209700	-3.14901300	2.42902700
H	4.73525900	-3.46177100	2.57818800
C	7.59321300	-1.84372100	0.54808300
H	6.06024200	-1.14443900	-0.77513300
C	7.88323000	-2.56643200	1.70047000
H	7.07107400	-3.71160400	3.32688400
H	8.39235200	-1.39959200	-0.03045500
H	8.90870500	-2.67788200	2.02703600
C	1.70068000	4.08634000	-0.49049100
C	2.20700600	4.43486500	0.76524600
C	1.64789600	5.05862400	-1.49313900
C	2.64979000	5.72678400	1.01069200
H	2.22710900	3.70151400	1.55912300
C	2.10533900	6.34626200	-1.24974100
H	1.26340300	4.79773500	-2.47034100
C	2.60505300	6.68441700	0.00315500
H	3.02566700	5.98644700	1.99143000
H	2.07078900	7.08560200	-2.03887100
H	2.95554200	7.68998400	0.19440500
H	-2.07543000	-1.19704000	0.95681000
C	-7.19342500	-0.87464700	1.07257200
C	-6.55813500	0.25735700	0.57113500
C	-5.25983400	0.57542100	0.94091000
C	-4.57795600	-0.25823800	1.86112400
C	-5.23212200	-1.40854200	2.35407300
C	-6.52005700	-1.71360500	1.96320000
C	-2.65425000	1.31667200	1.93655300
C	-3.29895500	2.19165600	1.02698200
C	-2.74065300	3.43705100	0.77321600
H	-3.24342900	4.11100500	0.08926200
C	-1.54697200	3.82876600	1.37155600
C	-0.88401000	2.95364100	2.23302200
C	-1.42799800	1.71613400	2.51101800
H	-8.20525300	-1.10631500	0.76702200
H	-7.07937100	0.89741300	-0.13099100
H	-4.70555400	-2.04945600	3.05063100
H	-7.00814500	-2.59934100	2.34857500
H	-1.12469000	4.80109100	1.15510800
H	0.05153600	3.24818300	2.68917200
H	-0.92615600	1.03843100	3.19039200
C	-3.27055600	0.08725800	2.29384200

H	-2.77678600	-0.54897800	3.01869100
C	-4.54719700	1.74352000	0.31747200
H	-5.23296200	2.58447200	0.19120600
H	-4.26661800	1.43604300	-0.69991900

INT2-2Sc(OTf)$_3$

Os	1.99223900	-0.57226400	-0.95877700
C	1.09036500	-2.36550300	-1.28844300
N	0.37990600	-3.29112600	-1.33533600
C	5.86361400	-2.63070700	-0.35132100
C	6.77113900	-1.58315300	-0.20362700
C	6.25151700	-0.24882900	-0.18006000
C	4.86023700	-0.08324000	-0.36271300
C	4.50239500	-2.38099700	-0.54826700
C	7.03888700	0.93173100	0.03335200
C	4.26836200	1.22461700	-0.31534200
C	5.06913100	2.37651400	-0.12770500
C	6.48171500	2.18029900	0.03235200
C	4.41321700	3.65102800	-0.14441300
C	3.03047700	3.66461500	-0.31660800
C	2.30767400	2.47862400	-0.46474600
H	8.10869000	0.82569600	0.20039800
H	6.21137900	-3.66334200	-0.35954500
H	3.78705500	-3.19254400	-0.67951500
H	7.12363400	3.04886800	0.16242600
H	2.47753100	4.60231300	-0.31275600
H	1.22510200	2.50863900	-0.58708100
N	4.02240900	-1.13926200	-0.58675400
N	2.91648900	1.29415400	-0.47943600
O	2.28049800	-0.26942300	-2.66580400
O	1.66731500	-0.89125100	0.90706300
C	0.07169200	0.09674600	-0.92628500
N	-1.01708300	0.43697600	-0.71880400
C	8.21428000	-1.88041300	-0.07191900
C	8.64295400	-2.87607900	0.82452800
C	9.17099200	-1.22062900	-0.86494300
C	9.99840000	-3.18255600	0.94711400
H	7.91010500	-3.39686400	1.44289500
C	10.52473500	-1.53997700	-0.74997700
H	8.85155400	-0.47803300	-1.59801300
C	10.94293000	-2.51420500	0.16162800
H	10.31781900	-3.94668700	1.65888400
H	11.25534100	-1.02852000	-1.38027700
H	12.00363800	-2.75719700	0.25518900
C	5.13013600	4.94089200	-0.01702600
C	6.06187100	5.18772400	1.00725300
C	4.83286600	5.97685500	-0.92236600
C	6.67828200	6.43433200	1.12223400
H	6.28616400	4.41466300	1.74116000
C	5.46083700	7.21790600	-0.81432500
H	4.11879000	5.79889100	-1.72878400
C	6.38406300	7.45130700	0.20935900
H	7.38798300	6.61170700	1.93296300
H	5.22710500	8.00596300	-1.53331500

H	6.87140600	8.42474600	0.29734300
Sc	-3.00278100	1.23314500	-0.43678700
O	-2.45526400	2.59144300	-1.81376200
O	-3.44890000	-0.23722500	0.97722500
O	-4.12691900	2.82622900	0.56542400
S	-3.02434900	3.22375600	1.53093600
S	-3.36868300	-1.42555300	1.89212100
S	-1.45059500	3.72334200	-2.09883100
O	-4.52173100	-1.62946300	2.74401700
O	-2.92204600	-2.64138400	1.12171800
O	-2.12049300	4.91500800	-2.60324600
O	-0.45657600	3.85595800	-1.03063300
O	-2.64714700	4.62299300	1.59226700
O	-1.93688900	2.20842600	1.25290500
C	-0.53812700	2.99657000	-3.56445200
C	-3.69291400	2.77983500	3.22203200
C	-1.89204500	-1.04726400	2.98496800
F	0.15928100	3.96105800	-4.15505600
F	0.30200400	2.04336300	-3.16056300
F	-1.39789000	2.47865800	-4.43320000
F	-4.29723100	1.60151500	3.17334100
F	-4.55762800	3.70912600	3.59637600
F	-2.68129900	2.73487200	4.07764300
F	-0.85595400	-0.75973200	2.21314600
F	-2.16884700	-0.00896100	3.76003100
F	-1.63934900	-2.10697200	3.73079300
Sc	-1.44387700	-3.42717000	-0.12894700
O	-2.24388100	-2.17538200	-1.59317400
O	-0.23236400	-3.49912700	1.45832700
O	-2.15133300	-5.19442400	-0.58268600
S	-1.71569100	-6.69279100	-0.54309200
S	1.12516100	-3.60066100	2.18286600
S	-3.30224800	-1.50413700	-2.42335900
O	2.24977400	-3.80484300	1.27080900
O	1.23511700	-2.62116000	3.26397800
O	-4.45771700	-2.31038500	-2.75985100
O	-3.62260700	-0.13270800	-1.87945400
O	-0.26563700	-6.81510600	-0.44996700
O	-2.46469600	-7.46853900	-1.52058700
C	-2.36697000	-1.11422500	-3.99786600
C	-2.40700100	-7.15364400	1.13950000
C	0.89273800	-5.23103400	3.06236900
F	-1.27910800	-0.41447300	-3.70810100
F	-2.02629200	-2.25395300	-4.57683100
F	-3.15160600	-0.41260500	-4.79831800
F	-3.72395900	-7.29182800	1.06183500
F	-2.12044600	-6.20485900	2.02945900
F	-1.86185200	-8.29848500	1.53008100
F	-0.17344400	-5.17360400	3.85268300
F	0.74188000	-6.21605500	2.18565300
F	1.97431600	-5.46523500	3.80191000
C	0.82402900	3.52660600	2.63340200
C	1.11851200	2.18399800	2.83193400
C	2.44886600	1.76272100	3.09262200

C	3.48433100	2.73965200	3.14226100
C	3.16146000	4.08618500	2.96197100
C	1.84635700	4.48478300	2.70590800
C	2.77043300	0.37930800	3.26967300
C	4.89763700	2.31941500	3.45836300
C	5.19283500	0.85087900	3.29912200
C	4.12241200	-0.08810400	3.27632500
C	4.42408600	-1.47527300	3.21591700
H	3.60176700	-2.19048400	3.19253400
C	5.74187900	-1.90919700	3.16849900
C	6.79218400	-0.97857600	3.18753700
C	6.50884600	0.38869600	3.25253700
H	1.96469800	-0.34661800	3.39880300
H	-0.19849800	3.83121000	2.41317600
H	0.32381800	1.43911100	2.77239200
H	3.95269200	4.83782200	3.01287700
H	1.61770600	5.54140300	2.55117200
H	5.61383500	2.90770600	2.86477900
H	5.95718400	-2.97871200	3.11354900
H	7.82871600	-1.31768700	3.14667800
H	7.32839200	1.11143600	3.27026100
H	5.11395800	2.60053500	4.50896200
H	2.22547300	-0.50356300	1.61316100

INT3

Os	1.41421600	-2.56902100	-1.01973000
C	3.43747700	-2.63337700	-1.27027400
N	4.58958700	-2.60826100	-1.37770800
C	1.88719100	1.81012700	-1.43348400
C	0.62587200	2.30813400	-1.15201600
C	-0.36439900	1.38346000	-0.71089800
C	-0.02996100	0.01630200	-0.70405800
C	2.15407600	0.44878700	-1.34534300
C	-1.67617900	1.74993900	-0.28017400
C	-1.01933600	-0.96835500	-0.39100600
C	-2.31902900	-0.57949200	-0.01288800
C	-2.60559700	0.81850000	0.04985300
C	-3.26866900	-1.60714200	0.24717100
C	-2.86012400	-2.92088700	0.09228500
C	-1.55316400	-3.22170700	-0.27848200
H	-1.92432600	2.79590400	-0.20409800
H	2.67526600	2.46946700	-1.76273700
H	3.13331900	0.05476700	-1.56971400
H	-3.58787800	1.13592000	0.36063800
H	-3.54770300	-3.73190100	0.28160200
H	-1.21931800	-4.24362000	-0.38409000
N	1.21451200	-0.43030800	-1.02181600
N	-0.65706400	-2.27367100	-0.50919100
O	1.04362600	-2.65985700	-2.72300200
O	1.67240300	-2.44841400	0.91230200
C	1.45585700	-4.60296600	-0.82162200
N	1.43735400	-5.74987900	-0.66792900
C	0.34366900	3.74213100	-1.35240100
C	1.26761200	4.70311600	-0.93152000

C	-0.81014800	4.16654800	-2.02060900
C	1.02847600	6.05338900	-1.13869000
H	2.16812400	4.38758100	-0.42686700
C	-1.04147000	5.51731800	-2.23959400
H	-1.51332300	3.43753000	-2.39816400
C	-0.12909600	6.46553100	-1.79031600
H	1.74710900	6.78415600	-0.79190600
H	-1.93330700	5.82825300	-2.76732300
H	-0.31453900	7.51865300	-1.95528400
C	-4.66077700	-1.32358400	0.65075000
C	-4.94386000	-0.51280800	1.75231800
C	-5.71826700	-1.90716700	-0.05239300
C	-6.25680300	-0.28888900	2.14181500
H	-4.13237000	-0.07698900	2.31583000
C	-7.03161700	-1.67019200	0.33027400
H	-5.50950100	-2.53122000	-0.91138500
C	-7.30426600	-0.86208400	1.42866400
H	-6.46102800	0.33134000	3.00458600
H	-7.84197200	-2.11725800	-0.23012100
H	-8.32798800	-0.68207300	1.72908800
H	2.27946900	-3.08300100	1.31390900
C	4.73756100	0.82010400	0.99633200
C	3.60969500	0.23451800	1.56300800
C	2.53520000	1.00280900	1.98885300
C	2.58096900	2.40956500	1.82757200
C	3.74553700	2.99067300	1.27792200
C	4.80549200	2.20829100	0.86465300
C	0.22806300	2.62520000	2.59358400
C	0.13640600	1.22773600	2.81281000
C	-1.06778300	0.68633000	3.24015600
H	-1.13523100	-0.38198600	3.41018400
C	-2.17972400	1.49116100	3.46606700
C	-2.10315300	2.86594000	3.23649400
C	-0.91856900	3.42386900	2.80149400
H	5.55507700	0.19817400	0.65711600
H	3.55572400	-0.84144900	1.66003000
H	3.79501100	4.06817400	1.17770200
H	5.68485200	2.67076500	0.43542600
H	-3.09970100	1.05452900	3.83032200
H	-2.97014000	3.49248800	3.40137100
H	-0.85038100	4.48922800	2.61838600
C	1.45172000	3.19770300	2.16482200
H	1.50417400	4.27084700	2.03541100
C	1.34993900	0.35416200	2.65007000
H	1.66229400	0.01908200	3.64930900
H	1.09578700	-0.56622200	2.12014000

INT4

Os	-0.81733700	-1.60213600	-0.90474300
C	-1.78399300	-3.26449400	-0.24013100
N	-2.30892900	-4.20872600	0.17959900
C	2.75441200	-3.46020300	0.93249000
C	3.67016900	-2.42244200	0.97928500
C	3.21897900	-1.13289400	0.58852700

C	1.88992100	-1.01352300	0.14001500
C	1.45521000	-3.25477700	0.47468000
C	4.01110100	0.05557500	0.64563000
C	1.37406400	0.25237500	-0.25833300
C	2.15245800	1.41835800	-0.13375300
C	3.50415600	1.26793900	0.30614700
C	1.54968700	2.65921900	-0.47364600
C	0.24984500	2.63677100	-0.95924000
C	-0.44265900	1.44171500	-1.09174800
H	5.03061900	-0.01501400	0.99131700
H	3.04985600	-4.45829200	1.22209700
H	0.74732600	-4.06868400	0.41969700
H	4.13033600	2.14426700	0.36764000
H	-0.24710800	3.55740100	-1.22737500
H	-1.45065000	1.40925500	-1.47687500
N	1.02771800	-2.06510200	0.07188100
N	0.09962800	0.27560400	-0.74643300
O	-0.06861800	-2.27021900	-2.37025500
O	-1.87516800	-0.71060900	0.89871200
C	-2.47382100	-0.99157900	-1.87821800
N	-3.42760500	-0.57475200	-2.39615700
C	5.05065800	-2.67823800	1.43751300
C	5.27142400	-3.35114400	2.64248100
C	6.15273600	-2.29314500	0.66796600
C	6.56309000	-3.61545800	3.07781900
H	4.42837000	-3.65793000	3.24728300
C	7.44307200	-2.56818000	1.09921600
H	5.99743800	-1.80122800	-0.28273600
C	7.65253400	-3.22382100	2.30788700
H	6.71763600	-4.12719400	4.01855400
H	8.28579400	-2.27460800	0.48734200
H	8.65918800	-3.43283600	2.64499400
C	2.24821700	3.95039100	-0.32547600
C	2.84330000	4.31468500	0.88586900
C	2.28106200	4.85047500	-1.39452600
C	3.45965200	5.55034800	1.02304000
H	2.79877800	3.63975900	1.72825800
C	2.91149800	6.07972300	-1.25948200
H	1.82895200	4.57566000	-2.33837900
C	3.50092100	6.43346700	-0.05060900
H	3.90480600	5.82463200	1.97026400
H	2.94213800	6.76131100	-2.09915600
H	3.98752700	7.39398700	0.05531600
H	-2.83317200	-0.83247300	0.87267600
C	-2.09790300	-4.67101900	4.02872500
C	-2.26401700	-3.31810000	4.00420800
C	-1.14082700	-2.44869500	3.90525000
C	0.17521900	-3.02656800	3.84884700
C	0.30345600	-4.44449000	3.87388500
C	-0.79880300	-5.24254200	3.95782200
C	1.15192400	-0.79783400	3.70775400
C	-0.16342300	-0.21611200	3.73315200
C	-0.28544800	1.19934100	3.62804600
H	-1.27602800	1.63642900	3.62951000

C	0.82180400	1.98901900	3.52296800
C	2.12152700	1.41488300	3.52984700
C	2.28064900	0.06468100	3.61874100
H	-2.95982500	-5.32172500	4.09303400
H	-3.25463600	-2.88319700	4.04781900
H	1.29541600	-4.87547100	3.81941900
H	-0.69194300	-6.31915000	3.96903200
H	0.71553200	3.06260600	3.43787800
H	2.98945400	2.05686100	3.46163200
H	3.26895300	-0.37621200	3.61482900
C	1.28225100	-2.18370800	3.77268900
H	2.27282600	-2.61817900	3.75608100
C	-1.27417300	-1.05745200	3.85464200
H	-2.26329800	-0.61880800	3.93043900
H	-1.57595500	-0.95885100	1.79264100

INT5

Os	-2.74831000	0.00006100	0.10159300
C	-4.06757400	1.46698800	-0.28743300
N	-4.67789400	2.41411400	-0.60124200
C	0.05247300	3.45686200	-0.08642900
C	1.31526400	2.86541300	-0.07775700
C	1.37831500	1.43703100	-0.07806900
C	0.15716800	0.71968700	-0.02330100
C	-1.10830400	2.67591300	-0.05020800
C	2.59597700	0.68319800	-0.18764800
C	0.15713000	-0.71970700	-0.02329600
C	1.37824000	-1.43711300	-0.07806400
C	2.59594100	-0.68334700	-0.18764200
C	1.31512100	-2.86548800	-0.07774000
C	0.05230400	-3.45687900	-0.08637900
C	-1.10843600	-2.67587400	-0.05016500
H	3.53517500	1.22261500	-0.30388600
H	-0.04296600	4.54332100	-0.08240000
H	-2.10671400	3.12068900	-0.04686200
H	3.53511100	-1.22281800	-0.30386900
H	-0.04318600	-4.54333300	-0.08233400
H	-2.10686700	-3.12060100	-0.04680800
N	-1.05750900	1.34397100	-0.00554200
N	-1.05757700	-1.34393200	-0.00552600
O	-2.71530400	0.00007500	1.85356000
C	-4.06765000	-1.46680500	-0.28740000
N	-4.67801000	-2.41391300	-0.60118800
C	2.52922900	3.71471800	-0.06987500
C	2.66286600	4.76059800	-0.99956600
C	3.54123300	3.52581000	0.88876000
C	3.78772600	5.58629900	-0.98167700
H	1.88669900	4.91212500	-1.75295900
C	4.66026900	4.35894500	0.91039400
H	3.43421300	2.73967200	1.63890300
C	4.78918400	5.38777300	-0.02725700
H	3.88280300	6.38761700	-1.71782600
H	5.43199800	4.20878800	1.66875700
H	5.66755200	6.03686300	-0.01114700

C	2.52905300	-3.71483900	-0.06987000
C	3.44255566	-3.64730505	-1.13617284
C	2.76647784	-4.63230042	0.96970334
C	4.57343951	-4.46472808	-1.15437636
H	3.27208773	-2.93361068	-1.94523202
C	3.89300096	-5.45516998	0.94412065
H	2.04743533	-4.72031442	1.78665797
C	4.80183647	-5.37036475	-0.11471011
H	5.28017552	-4.39265828	-1.98407426
H	4.05773333	-6.17214659	1.75160733
H	5.68507883	-6.01279178	-0.13141708

TS1

Os	-0.90440300	-1.75042900	-1.06838600
C	-1.41808400	-3.72233000	-0.90657800
N	-1.65610000	-4.84889100	-0.79429600
C	3.12039500	-3.14005200	0.29168000
C	3.84571600	-1.96875700	0.40656700
C	3.17810300	-0.74257500	0.12950500
C	1.83372700	-0.80234000	-0.28884300
C	1.79029200	-3.11211700	-0.12340000
C	3.78581800	0.54269400	0.26942900
C	1.12230800	0.40536700	-0.60105400
C	1.74930400	1.65843500	-0.45286500
C	3.10737500	1.68412000	-0.01018000
C	1.00001800	2.82261100	-0.78359800
C	-0.27269000	2.64891100	-1.29421000
C	-0.82407600	1.37724400	-1.40336900
H	4.80842900	0.59961700	0.60840300
H	3.58657800	-4.09373500	0.49110000
H	1.21814200	-4.02218000	-0.23375400
H	3.60406500	2.63653100	0.08881500
H	-0.87656300	3.50238300	-1.55895800
H	-1.83124000	1.23076600	-1.76356100
N	1.17150200	-1.98142700	-0.41604400
N	-0.15428500	0.29262100	-1.04968000
O	-0.27987700	-1.88346500	-2.69856000
O	-1.18625500	-1.47633200	0.72659000
C	-2.78072200	-1.26492800	-1.65630400
N	-3.81494000	-0.91551000	-2.04188200
C	5.26782800	-2.02475100	0.80391500
C	5.63993500	-2.71467100	1.96042900
C	6.26003400	-1.43614100	0.01467900
C	6.97585200	-2.79919600	2.32931200
H	4.87778200	-3.16998800	2.57915100
C	7.59587100	-1.53376900	0.37880000
H	5.98718700	-0.92363700	-0.89802500
C	7.95674200	-2.20995400	1.53938500
H	7.24996600	-3.32598300	3.23374500
H	8.35557900	-1.08464100	-0.24709600
H	8.99807400	-2.28021300	1.82463700
C	1.51819100	4.19002900	-0.58590500
C	2.04875500	4.58853100	0.64465700
C	1.42147400	5.12821700	-1.61719000

C	2.47300700	5.89555100	0.83729400
H	2.10333100	3.88110400	1.46029200
C	1.85980000	6.43147500	-1.42680900
H	1.01771800	4.82868800	-2.57538400
C	2.38452900	6.81914000	-0.19878000
H	2.86891100	6.19369000	1.79905000
H	1.79098300	7.14412800	-2.23796300
H	2.72068300	7.83655900	-0.04883900
H	-2.03711700	-0.77323500	1.23706300
C	-6.81293700	-1.37609800	1.31491500
C	-6.35628700	-0.20299800	0.72746800
C	-5.05855900	0.24700800	0.94187100
C	-4.20581900	-0.49104500	1.78028200
C	-4.67609800	-1.67486500	2.36902800
C	-5.96570600	-2.11742200	2.13801600
C	-2.51502700	1.35763900	1.72438900
C	-3.35182000	2.11455800	0.88457800
C	-3.04728600	3.45338800	0.65202100
H	-3.70076500	4.04076000	0.01790700
C	-1.92557600	4.04272300	1.21962900
C	-1.08210000	3.28768600	2.03277000
C	-1.37474800	1.95917800	2.27920300
H	-7.82508000	-1.71300500	1.13262800
H	-7.01510200	0.36885800	0.08507800
H	-4.01206100	-2.24209500	3.00979200
H	-6.31593800	-3.03299800	2.59625000
H	-1.70235300	5.08342200	1.02456100
H	-0.20515500	3.74158700	2.47338000
H	-0.72989600	1.36939400	2.91910100
C	-2.84445300	-0.03159600	2.02487600
H	-2.39448600	-0.41322800	2.94031000
C	-4.54717900	1.47212200	0.23901800
H	-5.34838200	2.20378800	0.11861400
H	-4.27162200	1.16184800	-0.77870400

TS1-2Sc(OTf)$_3$

Os	2.00134100	-0.56075200	-0.86560800
C	1.10513900	-2.36196400	-1.20400300
N	0.40151700	-3.29010200	-1.28678100
C	5.88215300	-2.63247500	-0.32665600
C	6.79372700	-1.58731900	-0.19100500
C	6.28060500	-0.25054500	-0.17714800
C	4.88885400	-0.07961200	-0.35291900
C	4.51954300	-2.37792000	-0.50803600
C	7.07681700	0.92680000	0.02115700
C	4.30626500	1.23420100	-0.32579300
C	5.11724300	2.38233000	-0.16058000
C	6.52841200	2.17902800	0.00336800
C	4.47266000	3.66230300	-0.19247200
C	3.09171600	3.68718400	-0.37526100
C	2.35836800	2.50526600	-0.50048600
H	8.14612900	0.81520000	0.18787400
H	6.22609000	-3.66642200	-0.32904200
H	3.79800800	-3.18708700	-0.61933100

H	7.17564600	3.04557600	0.12155300
H	2.54737700	4.62990000	-0.38781600
H	1.27601300	2.54345200	-0.61822300
N	4.04527900	-1.13507600	-0.54992900
N	2.95411500	1.31490500	-0.48130600
O	2.24027100	-0.32002600	-2.59738700
O	1.70111900	-0.86990700	0.91926600
C	0.08127700	0.13277800	-0.85227500
N	-1.00457800	0.49047300	-0.66041000
C	8.23592300	-1.88785900	-0.05541000
C	8.66074400	-2.86761800	0.85998000
C	9.19512800	-1.24543400	-0.85959900
C	10.01527700	-3.17604900	0.98912500
H	7.92558100	-3.37435100	1.48691500
C	10.54773200	-1.56678200	-0.73778100
H	8.87823000	-0.51480800	-1.60574900
C	10.96226900	-2.52543100	0.19199900
H	10.33185900	-3.92796300	1.71505400
H	11.28043800	-1.06924400	-1.37674100
H	12.02216200	-2.76999100	0.29069200
C	5.19936300	4.94638700	-0.06090400
C	6.11290200	5.18955000	0.98069700
C	4.92714800	5.98125900	-0.97521800
C	6.73623000	6.43211800	1.10333900
H	6.31888500	4.41747400	1.72138300
C	5.56259700	7.21775700	-0.85936600
H	4.22661300	5.80583300	-1.79401500
C	6.46757800	7.44783100	0.18133900
H	7.43153200	6.60708200	1.92698800
H	5.34894700	8.00511500	-1.58533700
H	6.96055300	8.41786800	0.27536100
Sc	-3.01485400	1.24665600	-0.41487600
O	-2.45468500	2.67322900	-1.70976700
O	-3.44214500	-0.25607800	0.97141300
O	-4.26173900	2.74216700	0.59416600
S	-3.20484600	3.18629900	1.59041300
S	-3.36706700	-1.42598800	1.90896400
S	-1.42947700	3.79290300	-1.97267200
O	-4.52363500	-1.61699000	2.75899300
O	-2.91540900	-2.65434600	1.16179000
O	-2.08185900	5.00910200	-2.43996100
O	-0.42430100	3.87986000	-0.91043300
O	-2.90013200	4.60195600	1.67336100
O	-2.05743100	2.23395000	1.32831000
C	-0.54252500	3.08655800	-3.46394300
C	-3.89494300	2.69365100	3.25936800
C	-1.89630900	-1.02844000	3.00301900
F	0.17851900	4.04973600	-4.02792300
F	0.27285900	2.09955100	-3.09237300
F	-1.41910100	2.61837900	-4.34368600
F	-4.42821200	1.48261200	3.18935100
F	-4.82388500	3.56970800	3.60849000
F	-2.91013800	2.70380200	4.14581500
F	-0.85301100	-0.76257100	2.23104700

F	-2.17213500	0.02833900	3.75212200
F	-1.65151500	-2.07205400	3.77320700
Sc	-1.42761300	-3.41603700	-0.09065900
O	-2.21299100	-2.14153600	-1.54375800
O	-0.23015800	-3.49384600	1.50763600
O	-2.15130400	-5.17317100	-0.56121200
S	-1.72078200	-6.67328900	-0.52843400
S	1.12092900	-3.60399500	2.24327300
S	-3.25673200	-1.48500900	-2.40339300
O	2.25139200	-3.79672000	1.33465300
O	1.22185500	-2.63560200	3.33332900
O	-4.40839000	-2.29489100	-2.74344700
O	-3.58151100	-0.10225600	-1.88885600
O	-0.27167300	-6.80070700	-0.42660200
O	-2.46586900	-7.44033700	-1.51574200
C	-2.29529100	-1.11856300	-3.96733800
C	-2.42430800	-7.14453900	1.14618600
C	0.88300600	-5.24663500	3.09826500
F	-1.26347300	-0.33792800	-3.68013300
F	-1.86617600	-2.26199900	-4.47554300
F	-3.09463900	-0.50878800	-4.82629900
F	-3.74297000	-7.26184100	1.06249900
F	-2.12655300	-6.21272800	2.05038400
F	-1.89779600	-8.30287700	1.52272500
F	-0.18651200	-5.19895100	3.88491700
F	0.73450500	-6.21874200	2.20695700
F	1.96097900	-5.49306100	3.83904500
C	0.77639900	3.50016000	2.48863200
C	1.08168400	2.15082000	2.61826900
C	2.40443200	1.72678000	2.87764200
C	3.42601300	2.69457200	3.00918300
C	3.09765600	4.05315500	2.90039800
C	1.79025500	4.45801100	2.63652000
C	2.71518600	0.29316300	2.95967800
C	4.84250800	2.28878900	3.32099200
C	5.15123600	0.82060800	3.20560300
C	4.11615100	-0.13207800	3.08783400
C	4.44168200	-1.50636100	3.05279600
H	3.63787700	-2.23469200	2.95232700
C	5.76469000	-1.92314700	3.12931600
C	6.79250800	-0.97559800	3.24133500
C	6.48031300	0.38202700	3.27955700
H	1.99498100	-0.31973900	3.52171300
H	-0.24467400	3.80384300	2.26077600
H	0.29336000	1.40665400	2.50052800
H	3.88497800	4.80252500	3.01329800
H	1.55841300	5.52052300	2.53520900
H	5.54056300	2.85484500	2.68563600
H	5.99949300	-2.98931600	3.09705300
H	7.83445800	-1.29733700	3.29288900
H	7.28014700	1.12124700	3.37120900
H	5.08243800	2.61676900	4.35070300
H	2.28334100	-0.23853400	1.87667800

TS2

Os	-1.27461900	-1.31256100	-1.00812100
C	-2.17140900	-3.11023600	-1.26226000
N	-2.65704100	-4.16324900	-1.32359400
C	2.14857500	-3.74333400	0.35742500
C	3.15256500	-2.80791500	0.55694700
C	2.78727800	-1.43453700	0.50004300
C	1.46618500	-1.11580500	0.12618600
C	0.86094900	-3.34802300	0.01799000
C	3.66963700	-0.35203100	0.80434600
C	1.05899400	0.24950900	-0.00792400
C	1.95396500	1.29712600	0.29140100
C	3.27698600	0.94344700	0.69781800
C	1.48864200	2.63355500	0.14041900
C	0.17904200	2.81109800	-0.27288600
C	-0.63719400	1.72066900	-0.56039100
H	4.67338400	-0.57543400	1.13015600
H	2.37011900	-4.79905900	0.40579800
H	0.08492100	-4.07233600	-0.18087400
H	3.98285600	1.72804900	0.91814900
H	-0.22743100	3.80590000	-0.37985200
H	-1.65307400	1.85340800	-0.90335000
N	0.53103900	-2.06958200	-0.13623900
N	-0.20984400	0.47220000	-0.44690600
O	-0.50437600	-1.29715900	-2.62792900
O	-2.13714100	-1.20812400	0.85058000
C	-2.94812400	-0.38580600	-1.70350200
N	-3.88623600	0.19722700	-2.05946500
C	4.54125300	-3.25690500	0.77920400
C	4.81268600	-4.31192600	1.65590200
C	5.60285100	-2.68760900	0.06664100
C	6.11163900	-4.76377400	1.84024800
H	4.00287900	-4.77112100	2.20327800
C	6.89986400	-3.14858700	0.24309300
H	5.40801300	-1.90404600	-0.65233500
C	7.16045500	-4.18142600	1.13702500
H	6.30404500	-5.57295300	2.53236000
H	7.70669000	-2.70476800	-0.32497900
H	8.17286400	-4.53633200	1.27746200
C	2.33982900	3.82229300	0.36182000
C	3.09257500	4.00151500	1.52587700
C	2.36814900	4.82426200	-0.61445000
C	3.84888400	5.15073200	1.70959600
H	3.06757100	3.25371800	2.30278900
C	3.13441900	5.96745000	-0.43520100
H	1.80159600	4.69443300	-1.52693800
C	3.87592300	6.13576500	0.72881600
H	4.41505600	5.27728900	2.62296200
H	3.15282600	6.72593600	-1.20657600
H	4.47014400	7.02867900	0.87110100
H	-2.93410200	-1.74425100	0.93957600
C	0.40542300	-4.81834700	3.64157500
C	-0.48910700	-3.78151700	3.46843200
C	-0.05513500	-2.45311000	3.49738600

C	1.33141000	-2.19152400	3.69035400
C	2.23067300	-3.27272500	3.86709800
C	1.77237300	-4.56382500	3.85207500
C	0.92038200	0.23831100	3.62525500
C	-0.47379000	0.01744800	3.43817400
C	-1.32104000	1.12472900	3.32998600
H	-2.37831500	0.96467600	3.16630300
C	-0.81767800	2.40597000	3.41580100
C	0.55674700	2.62998300	3.62102300
C	1.41140600	1.56543200	3.72231000
H	0.05093400	-5.84030200	3.61919800
H	-1.53879600	-3.99104700	3.30783600
H	3.28089400	-3.05751500	4.01346200
H	2.45514200	-5.39033700	3.99496500
H	-1.48638600	3.25157100	3.32514800
H	0.93016100	3.64223900	3.68786800
H	2.47203600	1.71301000	3.87812500
C	1.77945300	-0.86282200	3.73719300
H	2.83668200	-0.67973700	3.88817200
C	-0.98548200	-1.34465600	3.29038700
H	-1.94450100	-1.49596500	3.79260600
H	-1.38187400	-1.38580600	2.17465800

$^{3}OsO_2$

Os	-2.66608800	-0.17722400	0.00083000
C	-4.27811600	-1.42763100	0.04841200
N	-5.17884000	-2.15278000	0.07622200
C	0.42657200	-3.45015400	0.07513400
C	1.62371900	-2.76179000	0.00363400
C	1.57536400	-1.33938400	-0.00743300
C	0.30786600	-0.72251900	0.00680200
C	-0.78573600	-2.76164100	0.08947800
C	2.73193000	-0.50264100	-0.00819900
C	0.19542900	0.70681200	-0.01300100
C	1.35717000	1.50494700	-0.00134000
C	2.62687400	0.84988300	-0.00812500
C	1.20329200	2.91928600	-0.01045000
C	-0.08110700	3.42601300	-0.08493900
C	-1.18053500	2.57550200	-0.09744900
H	3.70774800	-0.96261600	-0.00266400
H	0.41606500	-4.53013300	0.08665300
H	-1.72947000	-3.28743700	0.12340400
H	3.51995200	1.45459600	-0.02222300
H	-0.24968200	4.49252300	-0.09718100
H	-2.18491400	2.96925200	-0.13101800
N	-0.83569800	-1.44401300	0.04304400
N	-1.05350700	1.25565000	-0.04713100
O	-2.43028400	-0.59018800	-1.73925700
O	-2.42876300	-0.46320700	1.76656200
C	-4.06300200	1.29572500	-0.05202100
N	-4.80257100	2.18462500	-0.08487600
C	2.89643300	-3.50989700	-0.05866000
C	3.17898600	-4.48326000	0.90289300
C	3.80965500	-3.29125400	-1.09404400

C	4.35947400	-5.21144200	0.83950500
H	2.47984100	-4.65531100	1.71070500
C	4.98252600	-4.03032500	-1.16235300
H	3.58919300	-2.55982000	-1.85996400
C	5.26319800	-4.98758700	-0.19320400
H	4.57262600	-5.95371900	1.59725100
H	5.67566100	-3.86065000	-1.97560200
H	6.18070800	-5.55871300	-0.24470300
C	2.35094500	3.84521400	0.05689800
C	3.29488400	3.74970000	1.08370200
C	2.47496700	4.86653500	-0.88880500
C	4.34296200	4.65630700	1.15838000
H	3.19242900	2.98204900	1.83878800
C	3.53316700	5.76232200	-0.81986700
H	1.75092000	4.94497600	-1.68916900
C	4.46845900	5.66022000	0.20399700
H	5.05979700	4.58057100	1.96516500
H	3.62629400	6.54043300	-1.56575300
H	5.28990100	6.36212700	0.26032300

$^{3}OsO_2/2Sc(OTf)_3$

Os	2.05930900	0.85907500	-0.17226400
C	0.66971300	2.37822600	-0.06711700
N	-0.24045000	3.10376000	-0.03975200
C	5.30652300	3.81270400	0.48701800
C	6.46121700	3.03069700	0.51902100
C	6.32333300	1.62952700	0.25254700
C	5.01828900	1.12604400	0.04432600
C	4.05162000	3.23499700	0.26963900
C	7.41612300	0.70502700	0.15814500
C	4.80693600	-0.26300800	-0.23230200
C	5.89981800	-1.15258100	-0.33573500
C	7.21386600	-0.62035100	-0.11494600
C	5.61546500	-2.52764300	-0.62959100
C	4.27654900	-2.90099200	-0.74549800
C	3.25311400	-1.95724400	-0.63841000
H	8.43038000	1.07318000	0.30051800
H	5.36121000	4.88442500	0.67704200
H	3.14300700	3.83815500	0.27388900
H	8.06842500	-1.29270400	-0.16166900
H	4.00019200	-3.93237500	-0.96311000
H	2.21467100	-2.26473800	-0.75888500
N	3.91208500	1.92332300	0.08308000
N	3.51214700	-0.66965700	-0.39489900
O	1.85341300	0.67936400	1.58424400
O	2.26878300	1.43201800	-1.99693300
C	0.46669900	-0.27860600	-0.66605000
N	-0.44971000	-0.92268800	-0.97100300
C	7.76593900	3.65875300	0.81786100
C	8.15034100	4.82973500	0.13989700
C	8.61355200	3.13295500	1.81098000
C	9.36618300	5.44780700	0.43328000
H	7.50263500	5.24222800	-0.63632800
C	9.82139000	3.76339800	2.11135600

H	8.31126100	2.24745400	2.37290600
C	10.20370900	4.91702600	1.41942700
H	9.66066700	6.34759900	-0.11090100
H	10.46426800	3.35423300	2.89355600
H	11.15287700	5.40479200	1.65193000
C	6.67511200	-3.54150300	-0.81078800
C	7.75400900	-3.31937100	-1.68704800
C	6.58032100	-4.77495700	-0.14041600
C	8.71931700	-4.30810000	-1.87929700
H	7.81778600	-2.38466400	-2.24679600
C	7.55679900	-5.75405500	-0.32391100
H	5.74911100	-4.95593600	0.54416500
C	8.62748100	-5.52346900	-1.19355500
H	9.54376100	-4.13027100	-2.57295500
H	7.48051800	-6.70146900	0.21372100
H	9.38791000	-6.29342100	-1.34122400
Sc	-2.24917800	-2.14110400	-1.16239100
O	-1.33222800	-3.43993400	0.06271600
O	-2.93179500	-0.69070700	-2.50988200
O	-3.35535500	-3.71135900	-2.16105100
S	-2.29890800	-4.02807900	-3.21281600
S	-2.83536700	0.62905800	-3.21454000
S	-0.07186800	-4.30280200	0.25911300
O	-3.97837800	0.97703000	-4.03107700
O	-2.37691900	1.68758000	-2.24136600
O	-0.41411200	-5.68399200	0.57075300
O	0.95167400	-4.02749800	-0.75250000
O	-1.90846200	-5.41367600	-3.37402700
O	-1.21642800	-3.00909500	-2.93157000
C	0.58280200	-3.56535600	1.84976500
C	-3.07860700	-3.50526700	-4.83723500
C	-1.33622600	0.46144800	-4.33999600
F	1.53539300	-4.35673000	2.33076400
F	1.10528500	-2.35899100	1.61489000
F	-0.38960500	-3.44975000	2.74480500
F	-3.71737800	-2.35456900	-4.69388100
F	-3.93248800	-4.44444700	-5.21443900
F	-2.12051900	-3.37744500	-5.74344700
F	-0.22778500	0.46624400	-3.62143600
F	-1.43899500	-0.68397800	-4.99924600
F	-1.33461600	1.46910600	-5.19147700
Sc	-2.35965300	2.95309500	-0.66710800
O	-2.32535400	1.26254400	0.61446200
O	-2.05006700	4.50885700	-1.88704900
O	-4.21329600	3.38024800	-0.16642500
S	-5.48389300	4.21897200	-0.48434000
S	-1.05291300	4.77251800	-3.04275600
S	-3.14335200	0.16700200	1.23674200
O	-0.44600300	6.09312200	-2.92914200
O	-0.21088600	3.60485400	-3.30650600
O	-4.46644500	0.52158400	1.70774000
O	-3.09353500	-1.07845400	0.38275800
O	-5.14515300	5.60250300	-0.79642300
O	-6.54418400	3.91389000	0.46687700

C	-2.09218900	-0.31190500	2.70838000
C	-5.99144800	3.42141600	-2.10189700
C	-2.25548700	4.87086900	-4.46827300
F	-2.57789700	-1.42026800	3.24154300
F	-0.84405400	-0.51263900	2.31038900
F	-2.12829000	0.67248100	3.59224000
F	-6.04546300	2.10059600	-1.96025800
F	-5.11144200	3.72544100	-3.05074600
F	-7.18852500	3.87686400	-2.45090200
F	-2.86875700	3.70137300	-4.62683100
F	-3.16188700	5.81373400	-4.24164400
F	-1.58343200	5.16397400	-5.57802600

^{3}INT5

Os	-2.71897600	-0.00596600	0.00836700
C	-4.12270800	1.41178100	-0.33878400
N	-4.87274500	2.26115200	-0.59240900
C	0.07319400	3.43810300	-0.13174900
C	1.32394300	2.85116800	-0.04897100
C	1.38898300	1.42934200	-0.03829500
C	0.17638200	0.71453400	-0.05159000
C	-1.07972700	2.65875500	-0.14751700
C	2.60794300	0.68364900	-0.03677600
C	0.17953400	-0.71656800	-0.02595900
C	1.39567000	-1.42548900	-0.03907900
C	2.61110200	-0.67380200	-0.03535600
C	1.33760100	-2.84764100	-0.02695500
C	0.08897000	-3.44058100	0.04447100
C	-1.06778200	-2.66699400	0.06065500
H	3.54456700	1.21897800	-0.04479400
H	-0.02483400	4.51363500	-0.14299400
H	-2.06148600	3.10794200	-0.18406200
H	3.55026000	-1.20444900	-0.02290800
H	-0.00379200	-4.51657600	0.05747600
H	-2.04716200	-3.12113100	0.09873200
N	-1.03566800	1.33339500	-0.09131600
N	-1.03022600	-1.34092600	0.01324400
O	-2.77889200	0.07424000	1.76904500
C	-4.11169900	-1.45941200	-0.20538900
N	-4.85612200	-2.33404700	-0.37617100
C	2.52745500	3.70227900	0.03128100
C	2.72170700	4.72382900	-0.90236300
C	3.45775500	3.53761200	1.06194700
C	3.83253800	5.55202200	-0.81704900
H	2.00904200	4.85600300	-1.70590800
C	4.55972400	4.37637800	1.15280900
H	3.30277000	2.77054300	1.80861800
C	4.75321200	5.38130000	0.21096400
H	3.97810500	6.33132600	-1.55328700
H	5.26509400	4.24757200	1.96302000
H	5.61570900	6.03084800	0.28043500
C	2.54648500	-3.69317800	-0.08577000
C	3.49356800	-3.53044100	-1.10124400
C	2.73154200	-4.70586800	0.85948200

C	4.60296500	-4.36191600	-1.16578500
H	3.34746700	-2.76963000	-1.85607600
C	3.84943300	-5.52675500	0.80047400
H	2.00655300	-4.83585800	1.65223600
C	4.78701700	-5.35765400	-0.21237300
H	5.32215900	-4.23403100	-1.96391100
H	3.98765900	-6.29852200	1.54600500
H	5.65565700	-6.00090400	-0.26071100

[3]INT6

Os	-2.70138700	-0.23271900	-0.56538000
C	-4.27708700	-1.53068500	-0.52655400
N	-5.15691200	-2.28105700	-0.50427400
C	0.50898500	-3.39621700	-0.54894700
C	1.67632600	-2.66804000	-0.68277900
C	1.57689400	-1.24974100	-0.73081700
C	0.29023300	-0.67779200	-0.66737600
C	-0.72663900	-2.75113500	-0.50961900
C	2.70224600	-0.37418200	-0.79569300
C	0.12844800	0.74596800	-0.65982600
C	1.26222700	1.58208100	-0.67029500
C	2.55084400	0.97372400	-0.77032600
C	1.06302900	2.98734900	-0.58437100
C	-0.23844800	3.45395000	-0.57843900
C	-1.30946300	2.56832800	-0.59592700
H	3.69205900	-0.80003000	-0.84622600
H	0.53947100	-4.47481600	-0.50311500
H	-1.64902000	-3.30891100	-0.42978400
H	3.42002000	1.61081900	-0.81834000
H	-0.44126900	4.51285300	-0.51730400
H	-2.32651800	2.92846300	-0.56916900
N	-0.82522600	-1.43759200	-0.57953500
N	-1.13795500	1.25222000	-0.61485700
O	-2.47964900	-0.60600800	-2.31633500
O	-2.44042600	-0.57516200	1.18525700
C	-4.14476400	1.19415500	-0.56073500
N	-4.91180900	2.06005500	-0.55871500
C	2.97457300	-3.36983900	-0.75019800
C	3.32581200	-4.27164300	0.25712400
C	3.84602500	-3.17471800	-1.82498900
C	4.53397200	-4.95354400	0.19832200
H	2.65810800	-4.42249300	1.09526100
C	5.04678400	-3.86830900	-1.88772700
H	3.57212900	-2.49784300	-2.62335200
C	5.39617900	-4.75446000	-0.87418000
H	4.80106900	-5.64022300	0.99061900
H	5.70820600	-3.71879200	-2.73079500
H	6.33532500	-5.28961800	-0.92183900
C	2.18469400	3.93983300	-0.47926900
C	3.15080100	3.79547800	0.52114100
C	2.26136000	5.03005800	-1.34947600
C	4.17536300	4.72340400	0.64342800
H	3.08249800	2.97220100	1.21956800
C	3.29680900	5.94744200	-1.23404700

H	1.51902400	5.14609100	-2.12821500
C	4.25473900	5.79707000	-0.23729100
H	4.90981300	4.60964700	1.42962900
H	3.35454600	6.78014400	-1.92233900
H	5.05812700	6.51580400	-0.14458900
C	-0.92316300	-0.54469700	4.43061400
C	0.23985400	-1.52186800	4.23872900
C	0.94911200	-1.28646100	2.90358700
C	1.41131700	0.16573400	2.77386800
C	0.24258300	1.13656400	2.94773500
C	-0.46827000	0.90979900	4.28344000
H	1.79774200	-1.96695400	2.79409000
H	0.96035800	-1.39283200	5.05480000
H	-0.12093200	-2.55202600	4.29831600
H	-1.68917000	-0.75278200	3.67786900
H	-1.38546700	-0.69736100	5.40937300
H	2.16520800	0.37180000	3.54252200
H	1.90218700	0.32410500	1.81330400
H	0.59065600	2.16992800	2.86815000
H	-0.47807900	0.98011000	2.13916900
H	0.21712900	1.16022700	5.10158300
H	-1.32504000	1.58240800	4.37399800
H	0.25286800	-1.51495300	2.09136100

[3]INT6-2Sc(OTf)$_3$

Os	2.44577900	0.83753600	0.38929700
C	1.00874700	2.27586000	0.78466400
N	0.07654200	2.93861300	1.00061200
C	5.62154500	3.93310100	0.58510300
C	6.79323000	3.22178600	0.32599200
C	6.67096100	1.82634500	0.02249400
C	5.37879300	1.25458700	0.07545400
C	4.37878000	3.29180200	0.60758000
C	7.75980600	0.97364000	-0.35847300
C	5.18195100	-0.13254700	-0.21398600
C	6.26642800	-0.95096700	-0.59782600
C	7.56771600	-0.34957200	-0.65001000
C	5.99189300	-2.32809600	-0.89152400
C	4.68719500	-2.78244300	-0.70335700
C	3.66611100	-1.90482500	-0.33769000
H	8.76063600	1.39605700	-0.42421000
H	5.66262900	4.99741100	0.81572800
H	3.46276600	3.83934400	0.83216800
H	8.42179700	-0.96725200	-0.92143900
H	4.41960000	-3.81895800	-0.90648200
H	2.64211600	-2.26668300	-0.25812900
N	4.26837200	1.98259400	0.38836000
N	3.90295300	-0.60828900	-0.11515600
O	2.47083200	0.61534800	2.15336400
O	2.33630300	1.55434700	-1.38594800
C	0.87003400	-0.36442000	0.04092100
N	-0.04889900	-1.03116500	-0.19905800
C	8.09805900	3.91539100	0.36792600
C	8.26300900	5.13744800	-0.30901100

C	9.16984100	3.39922600	1.12031000
C	9.48061400	5.81605200	-0.25273800
H	7.43958900	5.54333400	-0.90017000
C	10.38050600	4.08941400	1.18589300
H	9.04422700	2.47256700	1.68306000
C	10.54123600	5.29455100	0.49481400
H	9.60145500	6.75606200	-0.79518600
H	11.20021200	3.68635500	1.78433900
H	11.49222100	5.82955100	0.54250100
C	7.02211800	-3.25426900	-1.40149700
C	7.82867300	-2.90261500	-2.50060600
C	7.16087100	-4.53265300	-0.83074200
C	8.75779300	-3.81006900	-3.00986700
H	7.70261900	-1.93059400	-2.98045100
C	8.10379700	-5.42952400	-1.33264400
H	6.54155300	-4.81354400	0.02350900
C	8.90299100	-5.07116900	-2.42312300
H	9.36674500	-3.53254500	-3.87273900
H	8.21365300	-6.41329900	-0.87166800
H	9.63562400	-5.77746500	-2.81962700
Sc	-1.83978600	-2.24923700	-0.39722700
O	-0.81292600	-3.72732100	0.48354500
O	-2.72734200	-0.58916700	-1.31142400
O	-2.96785900	-3.65172900	-1.60239900
S	-1.97597300	-3.72707700	-2.75615600
S	-2.99461500	0.74796400	-1.93110000
S	0.50966200	-4.51475800	0.50111400
O	-4.22505200	0.85451500	-2.68470000
O	-2.77562400	1.84229600	-0.91703600
O	0.27390800	-5.94568100	0.63601300
O	1.45581100	-4.03549000	-0.51091600
O	-1.54607600	-5.03982900	-3.19093100
O	-0.91819800	-2.72161300	-2.35734000
C	1.20493900	-3.95111100	2.14922700
C	-2.87337800	-2.95218400	-4.20881500
C	-1.55357200	1.00488900	-3.10683700
F	2.16210100	-4.79205900	2.52315300
F	1.72942300	-2.72865800	2.02913200
F	0.25319000	-3.92483900	3.07302000
F	-3.56376300	-1.89497200	-3.80844700
F	-3.69456800	-3.85045000	-4.72856300
F	-1.97505500	-2.58667600	-5.11281600
F	-0.44061200	1.15497500	-2.40760500
F	-1.45371800	-0.06166500	-3.88782800
F	-1.78456600	2.07425800	-3.84339800
Sc	-2.12571600	2.78037500	0.76161100
O	-1.78871600	0.86538200	1.61084500
O	-2.12935300	4.53925000	-0.19480700
O	-3.79262000	3.01247700	1.75771400
S	-5.33234400	3.19168900	1.86125500
S	-1.46988500	5.09013400	-1.48135500
S	-2.50359000	-0.26830300	2.29540600
O	-1.01672300	6.46391000	-1.30014900
O	-0.57560000	4.10624200	-2.09269400

O	-3.77938400	0.03386900	2.91059300
O	-2.49846600	-1.49487300	1.41389400
O	-5.71556000	4.56491400	1.55631500
O	-5.85502400	2.52541600	3.04518600
C	-1.28545600	-0.73590600	3.63439900
C	-5.88843600	2.16897700	0.39038200
C	-2.97656000	5.18723500	-2.58566900
F	-1.69870000	-1.84304100	4.22772800
F	-0.09152000	-0.93385800	3.09307600
F	-1.22021000	0.25377400	4.51144400
F	-5.31770100	0.96322700	0.41405000
F	-5.55137800	2.78903100	-0.73451500
F	-7.20620500	2.02634800	0.43981700
F	-3.54741000	3.99094700	-2.70125100
F	-3.85640500	6.04212100	-2.07774300
F	-2.59896100	5.60713500	-3.78966800
C	2.86500300	-1.93681500	-3.82308800
C	4.17549100	-1.14531900	-3.74817900
C	4.28508500	-0.12878300	-4.88977800
C	3.07037200	0.80675600	-4.91767300
C	1.75881700	0.01695600	-4.99545700
C	1.65048800	-1.00460700	-3.85859900
H	5.21725600	0.45365900	-4.79398100
H	4.20854500	-0.59475600	-2.79266600
H	5.04175800	-1.82759400	-3.75169900
H	2.87380800	-2.55982900	-4.73653300
H	2.77771100	-2.63524100	-2.97475700
H	3.06290200	1.41564400	-3.99594500
H	3.14731500	1.51044400	-5.76353600
H	0.89869100	0.70536500	-4.97714200
H	1.71068000	-0.51374200	-5.96474200
H	1.58795000	-0.46313800	-2.89880500
H	0.72226700	-1.58801200	-3.94024600
H	4.35080700	-0.66967000	-5.85192600

^{3}INT7

Os	-0.06965700	-2.64293000	-0.49548700
C	0.89044700	-4.34800600	0.06229200
N	1.49097000	-5.28285300	0.38723100
C	3.88793000	-0.78103500	0.34244900
C	3.64030600	0.57330100	0.21698300
C	2.31265300	0.98059300	-0.09733600
C	1.34609200	-0.02231600	-0.30599500
C	2.87355500	-1.71120400	0.12627900
C	1.90206900	2.34580100	-0.19004100
C	-0.00075900	0.33755300	-0.64666200
C	-0.37402100	1.69294200	-0.73623800
C	0.62449400	2.68533100	-0.49636500
C	-1.72238300	1.99276300	-1.08208700
C	-2.56011200	0.93700500	-1.39215600
C	-2.11566700	-0.37720300	-1.27653100
H	2.63071300	3.12009500	-0.00753200
H	4.87990500	-1.13613900	0.57992100
H	3.05881900	-2.77237400	0.20806500

H	0.35209200	3.72622900	-0.57409000
H	-3.58785300	1.11686500	-1.67051900
H	-2.77211300	-1.21257600	-1.47209900
N	1.64516100	-1.34501300	-0.20100100
N	-0.88625600	-0.66691500	-0.88245300
O	0.30611100	-2.74502900	-2.19680400
O	-0.39423200	-2.18086000	1.36313800
C	-1.86155100	-3.58669100	-0.61627000
N	-2.92028700	-4.05213300	-0.67622900
C	4.74072700	1.53979000	0.40849600
C	5.51528700	1.48925400	1.57019300
C	5.05272100	2.48054300	-0.57723300
C	6.57001200	2.37401000	1.74999000
H	5.27705500	0.76715400	2.34007100
C	6.11672200	3.35410100	-0.40070700
H	4.47818900	2.51003900	-1.49335300
C	6.87382000	3.30730200	0.76508600
H	7.15446300	2.33360600	2.65957300
H	6.35669500	4.06919900	-1.17633500
H	7.69911100	3.99314800	0.90354900
C	-2.25363700	3.36902600	-1.08713100
C	-2.10969400	4.19191600	0.03406000
C	-2.96853600	3.84192500	-2.19056900
C	-2.66906500	5.46153800	0.04862800
H	-1.58442600	3.82601400	0.90549600
C	-3.51308800	5.11866400	-2.17918200
H	-3.08123500	3.21342100	-3.06411500
C	-3.36671100	5.93028800	-1.05942500
H	-2.56359900	6.08298300	0.92790800
H	-4.05286900	5.47894800	-3.04480000
H	-3.79726900	6.92279400	-1.04897600
C	-4.12372000	-0.87391900	1.82394200
C	-2.85776700	-0.80331900	2.60473900
C	-2.11206200	0.48756100	2.58575600
C	-3.05116200	1.66036000	2.94898300
C	-4.31086600	1.64488500	2.08309900
C	-5.05082600	0.31037600	2.18573200
H	-1.25245800	0.46095900	3.25614200
H	-2.69719300	-1.49771100	3.42143200
H	-1.29325300	-1.93027600	1.64512200
H	-3.90730400	-0.80446500	0.75084800
H	-4.63524800	-1.82389200	1.98015000
H	-3.33344500	1.57724100	4.00278700
H	-2.51729000	2.60688500	2.83513100
H	-4.97385100	2.46276400	2.37574400
H	-4.03449800	1.82754600	1.03977200
H	-5.41629600	0.17634300	3.20838800
H	-5.92428000	0.30406600	1.52922800
H	-1.72207300	0.68052700	1.57933700

^{3}INT8

Os	-2.22957300	-1.11853500	-0.73194900
C	-3.11190500	-2.89847400	-0.33069600
N	-3.56515000	-3.92167600	-0.02027900

C	1.51732000	-3.36928600	0.03349900
C	2.50480800	-2.40326600	-0.04458100
C	2.09923600	-1.06110200	-0.28599900
C	0.72748000	-0.81314100	-0.48293700
C	0.17905800	-3.03795400	-0.16314900
C	2.99339700	0.05424000	-0.29964500
C	0.26060900	0.52120300	-0.71326700
C	1.16151500	1.60308400	-0.69805900
C	2.54786000	1.32107900	-0.49668600
C	0.63460100	2.91054800	-0.88573100
C	-0.71947500	3.02879600	-1.14423400
C	-1.54362900	1.90720200	-1.14037100
H	4.04521700	-0.12139300	-0.13579100
H	1.77755200	-4.40159200	0.21659800
H	-0.59760500	-3.78758400	-0.11977600
H	3.25074100	2.13970800	-0.50506600
H	-1.16204600	4.00103700	-1.30410900
H	-2.60837100	1.99170200	-1.30223000
N	-0.20906100	-1.79833400	-0.42878400
N	-1.07685400	0.68908300	-0.89839200
O	-2.13594900	-1.36667800	-2.49638900
O	-2.30740900	-0.98657500	1.55633200
C	-4.03648500	-0.20772600	-0.77579700
N	-5.04202600	0.37282000	-0.76460200
C	3.92106300	-2.78506900	0.12723000
C	4.31132600	-3.52710800	1.24509400
C	4.87837200	-2.45344200	-0.83592900
C	5.63494600	-3.91473800	1.40464200
H	3.57788200	-3.78446200	1.99800500
C	6.19823200	-2.85379200	-0.68044200
H	4.58300100	-1.90420500	-1.71980000
C	6.58117100	-3.57984300	0.44237900
H	5.92663800	-4.47857600	2.28081300
H	6.92712500	-2.60181800	-1.43927400
H	7.61161800	-3.88627800	0.56472900
C	1.47298000	4.12222700	-0.79426500
C	2.24491100	4.37113400	0.34437800
C	1.46186200	5.06223400	-1.82756100
C	2.99299100	5.53595000	0.44357100
H	2.23744000	3.66363700	1.16278500
C	2.22233400	6.21990500	-1.73147600
H	0.86964800	4.87510300	-2.71370900
C	2.98846100	6.45991200	-0.59609400
H	3.57662200	5.72373500	1.33505700
H	2.21585100	6.93452000	-2.54380600
H	3.57678200	7.36475500	-0.51995800
C	-2.78932600	1.32994700	2.25345800
C	-2.03881100	0.04572900	2.56160100
C	-0.53844600	0.25069800	2.65191600
C	-0.19826800	1.34118400	3.67066200
C	-0.92753500	2.64467500	3.34396000
C	-2.43834600	2.42068200	3.26964700
H	-0.05398900	-0.69388500	2.90473200
H	-2.39680600	-0.36899000	3.50716600

H	-3.09907700	-1.47553200	1.81224300
H	-2.51353500	1.66730800	1.25407500
H	-3.86299800	1.13776000	2.23965500
H	-0.48277300	1.00788700	4.67396800
H	0.88216200	1.49734300	3.68627900
H	-0.69483900	3.40492300	4.09258600
H	-0.56973300	3.02713600	2.38136100
H	-2.80960600	2.13257500	4.25872300
H	-2.95172100	3.34543100	2.99993000
H	-0.16302500	0.55825100	1.67704600

^{3}INT8-2Sc(OTf)$_3$

Os	2.16276200	0.39209200	0.75085800
C	0.80074900	1.81559800	1.13766900
N	-0.09097500	2.55972800	1.30064100
C	5.24218500	3.59639800	0.71041100
C	6.40214200	2.93336900	0.31649800
C	6.30883000	1.52898800	0.05444100
C	5.05794700	0.90018800	0.26338500
C	4.04156400	2.89818100	0.88412200
C	7.38612600	0.73056400	-0.45772500
C	4.89248000	-0.50036400	-0.01883400
C	5.96435400	-1.25766500	-0.54593100
C	7.22275800	-0.59646100	-0.74130900
C	5.71933100	-2.63396900	-0.85710700
C	4.47016100	-3.15767200	-0.53371400
C	3.45862200	-2.33732700	-0.02025300
H	8.35041700	1.20227200	-0.63750200
H	5.26177700	4.66675000	0.91504000
H	3.13214800	3.41208700	1.19788800
H	8.06403200	-1.17148900	-1.12438800
H	4.23632500	-4.20300300	-0.73554800
H	2.46654300	-2.74594000	0.17822300
N	3.95419800	1.58580700	0.68253000
N	3.65625500	-1.03737500	0.19433500
O	2.37084800	-0.00548300	2.48249300
O	1.90590300	0.96210100	-1.39254700
C	0.58979500	-0.80779200	0.45002700
N	-0.33728000	-1.46385800	0.18546300
C	7.66556200	3.68949900	0.16123900
C	7.68039300	4.87354400	-0.59711700
C	8.84897000	3.26849800	0.79464000
C	8.85809700	5.60965200	-0.73482100
H	6.76812200	5.20517800	-1.09751500
C	10.02134900	4.01435700	0.66480000
H	8.84419300	2.36979100	1.41422200
C	10.03067700	5.18227400	-0.10440000
H	8.85952400	6.52024700	-1.33779500
H	10.93076600	3.68374600	1.17116400
H	10.95104200	5.76096800	-0.20940800
C	6.72684300	-3.48700600	-1.52592500
C	7.33858600	-3.06946700	-2.72244000
C	7.03644000	-4.75564500	-1.00587900
C	8.24608700	-3.90246800	-3.37795300

H	7.07830900	-2.10231100	-3.15685100
C	7.95476000	-5.58016800	-1.65768600
H	6.56761400	-5.08723700	-0.07702400
C	8.56098100	-5.15624200	-2.84437300
H	8.70436200	-3.57282500	-4.31287200
H	8.19699500	-6.55850900	-1.23712500
H	9.27520600	-5.80493500	-3.35631800
Sc	-2.16367700	-2.57572600	0.06911100
O	-1.24262600	-4.02131400	1.09886000
O	-2.98541500	-0.96502300	-1.02338500
O	-3.44461400	-4.06833500	-0.85089100
S	-2.56531300	-4.33162100	-2.06383400
S	-3.06317400	0.22910400	-1.92233700
S	0.08205600	-4.77422200	1.32295300
O	-4.29861600	0.37692800	-2.66280300
O	-2.61563400	1.47374600	-1.19083900
O	-0.15967000	-6.17281100	1.65479900
O	1.08675000	-4.44171600	0.31053000
O	-2.20355900	-5.70483700	-2.35134500
O	-1.45122200	-3.31729100	-1.91366600
C	0.66428600	-3.96705500	2.91446200
C	-3.60428300	-3.74793000	-3.50853500
C	-1.67639300	-0.00590900	-3.16856400
F	1.53800800	-4.77352900	3.50832500
F	1.25727900	-2.80583200	2.65148000
F	-0.36385800	-3.75702400	3.73002000
F	-4.15677200	-2.57625500	-3.22770000
F	-4.55344800	-4.64403700	-3.73177900
F	-2.83163800	-3.63642800	-4.57904800
F	-0.53207700	-0.21717400	-2.53734900
F	-1.96244500	-1.04887600	-3.93240800
F	-1.59243500	1.08141000	-3.91552600
Sc	-2.12036700	2.51228000	0.49364600
O	-2.22017900	0.70226500	1.52048700
O	-1.48868800	4.07908200	-0.69408200
O	-3.76791900	3.27753900	1.19235800
S	-5.29906600	3.50989800	1.35894400
S	-0.35859300	4.01735700	-1.72242200
S	-2.95470300	-0.27872000	2.39264300
O	0.71487500	4.97061600	-1.48229000
O	0.00783200	2.60630300	-1.99138000
O	-4.26341500	0.12403300	2.86293600
O	-2.88436700	-1.65615100	1.77888300
O	-5.65694900	4.85313300	0.92282900
O	-5.76825900	2.99342800	2.63575000
C	-1.80192800	-0.43415800	3.85795800
C	-5.94930300	2.35468300	0.03312200
C	-1.25578700	4.54302200	-3.27291900
F	-2.26243300	-1.36896100	4.67192000
F	-0.59190100	-0.76051800	3.43203500
F	-1.75918200	0.73327800	4.48223900
F	-5.55578500	1.10358700	0.26816400
F	-5.48864700	2.73950000	-1.15232000
F	-7.27395600	2.40352200	0.03283800

F	-2.33751500	3.79314400	-3.43618600
F	-1.60658400	5.81743700	-3.17479300
F	-0.44261700	4.38255500	-4.31006300
C	3.65388900	-0.57126300	-4.39940000
C	4.78011800	0.46004100	-4.53425500
C	4.27880400	1.87421800	-4.22001300
C	3.63391800	1.93891700	-2.83074100
C	2.53365800	0.89024900	-2.69800800
C	3.02114600	-0.51720800	-3.00400300
H	5.10267400	2.60277500	-4.28261200
H	5.59520900	0.20016500	-3.83361000
H	5.21184100	0.42419300	-5.54733900
H	2.88160100	-0.37219000	-5.16353100
H	4.03298500	-1.58730700	-4.59248600
H	4.40142800	1.75309000	-2.06370400
H	3.20824800	2.93559200	-2.63245700
H	1.16686100	1.61166600	-1.49015400
H	1.72955100	1.13704400	-3.41054000
H	3.78252600	-0.80832600	-2.26985600
H	2.18385400	-1.22543200	-2.91013700
H	3.53624300	2.17413400	-4.98086000

^{3}TS3

Os	-2.78470300	0.11390400	-0.39039100
C	-4.35106200	-1.13888500	-0.02134500
N	-5.20078500	-1.89231400	0.20327400
C	-0.29312700	-3.54861700	-0.14841800
C	0.98563100	-3.09369300	-0.42035200
C	1.16778600	-1.69416100	-0.59868000
C	0.02773400	-0.87045100	-0.53982700
C	-1.36948100	-2.66693400	-0.10863200
C	2.43967000	-1.06688400	-0.77280500
C	0.15195700	0.54809200	-0.67022900
C	1.42025400	1.14117100	-0.81504700
C	2.55961000	0.28159200	-0.87634600
C	1.48529000	2.56022000	-0.89579400
C	0.29330400	3.26301500	-0.88129400
C	-0.92227700	2.60072000	-0.73481500
H	3.32354500	-1.68441900	-0.80451000
H	-0.47718400	-4.60223000	0.00108900
H	-2.37538900	-3.01439300	0.07679500
H	3.53535000	0.72236500	-1.00857000
H	0.29439400	4.34148700	-0.93745100
H	-1.85572100	3.14238000	-0.69128800
N	-1.21654400	-1.36702000	-0.31640600
N	-0.99117800	1.28204900	-0.61590600
O	-2.88061000	-0.09224800	-2.13790900
O	-2.64093900	0.26711600	1.53530800
C	-4.11117900	1.66401500	-0.35511500
N	-4.81588900	2.58201000	-0.33317200
C	2.10420700	-4.05305800	-0.50017900
C	2.31116000	-4.96627400	0.53715300
C	2.94094000	-4.09243400	-1.61934400
C	3.34582500	-5.88885900	0.46399000

H	1.67151300	-4.93849100	1.40952500
C	3.96513800	-5.02581600	-1.69581600
H	2.77183500	-3.40985500	-2.44133700
C	4.17403400	-5.92161000	-0.65252900
H	3.50453900	-6.58220000	1.27920700
H	4.59789200	-5.05577700	-2.57286000
H	4.97687300	-6.64453000	-0.71129400
C	2.76580200	3.29090400	-0.96454500
C	3.75533000	3.09010500	0.00229200
C	2.97982600	4.23417600	-1.97259900
C	4.93647500	3.81718500	-0.04220800
H	3.58811100	2.38474000	0.80479600
C	4.16908400	4.94863900	-2.02320500
H	2.22015800	4.39273000	-2.72675300
C	5.14900600	4.74292300	-1.05823700
H	5.68927300	3.66284100	0.71936200
H	4.32964000	5.66670500	-2.81633700
H	6.07314700	5.30430800	-1.09548500
C	0.20032400	1.94012500	2.71615300
C	-0.40241800	0.56631800	2.93237100
C	0.61770800	-0.55111300	2.84146000
C	1.74782100	-0.31109700	3.85727400
C	2.38481100	1.06242700	3.64777800
C	1.34480300	2.18071800	3.71586000
H	0.14919100	-1.52235400	3.00995000
H	-0.94392800	0.52194700	3.88206100
H	-1.26191800	0.39776000	2.17014600
H	0.60578200	2.01126500	1.70562600
H	-0.56008700	2.71651500	2.81368100
H	1.34227600	-0.37540500	4.87185100
H	2.49627500	-1.10139500	3.76200800
H	3.16690300	1.23223000	4.39195300
H	2.87390800	1.08200700	2.66756000
H	0.93263800	2.23151700	4.72851300
H	1.80753400	3.14921800	3.51157400
H	1.06013000	-0.57247200	1.84434900

^{3}TS3-2Sc(OTf)$_3$

Os	2.03486300	0.15327200	0.98428200
C	0.60239700	1.56094400	1.47982500
N	-0.35204300	2.22102200	1.59074300
C	5.01570700	3.40444200	0.57846000
C	6.14282400	2.75562600	0.08233300
C	6.06264100	1.34093100	-0.12624500
C	4.84348800	0.70067100	0.19149400
C	3.84733000	2.69376100	0.87815300
C	7.11258400	0.53886200	-0.68439500
C	4.67095300	-0.69989600	-0.04876500
C	5.71126900	-1.46562100	-0.61928000
C	6.94925800	-0.80082900	-0.90787100
C	5.45498400	-2.85228300	-0.87319300
C	4.22855800	-3.37247000	-0.45800100
C	3.24802400	-2.54997900	0.10154200
H	8.05612100	1.01458400	-0.94459600

H	5.02958800	4.48079200	0.74780700
H	2.95929800	3.19819600	1.25335000
H	7.77324700	-1.38006000	-1.32000800
H	3.99190100	-4.42452600	-0.61557300
H	2.28091300	-2.96218000	0.39857600
N	3.77241600	1.37779400	0.69892800
N	3.45653100	-1.23971900	0.27498000
O	2.33943700	-0.01734800	2.72105800
O	1.54459100	0.72123100	-0.79500900
C	0.45576200	-1.07669500	0.78204500
N	-0.47945700	-1.70330800	0.50191500
C	7.34836900	3.53731000	-0.27081500
C	7.22126000	4.64700900	-1.12549300
C	8.61501400	3.20743400	0.24304900
C	8.34399700	5.39898100	-1.47486500
H	6.24228700	4.90457100	-1.53474700
C	9.73188800	3.97195900	-0.09705500
H	8.72077200	2.36728900	0.93201100
C	9.60048000	5.06373400	-0.96140600
H	8.23585300	6.24939400	-2.15136500
H	10.70903000	3.71532200	0.31765300
H	10.47779000	5.65532900	-1.23208100
C	6.42619700	-3.72215000	-1.56721600
C	6.99683000	-3.32772100	-2.79234900
C	6.74530200	-4.98529600	-1.03712300
C	7.87379400	-4.17827700	-3.46561600
H	6.72451000	-2.36803400	-3.23515900
C	7.63602700	-5.82501100	-1.70617900
H	6.31034700	-5.29821000	-0.08569700
C	8.20112100	-5.42428000	-2.92112800
H	8.29876100	-3.86853900	-4.42266500
H	7.88908700	-6.79689800	-1.27738600
H	8.89322600	-6.08573300	-3.44683400
Sc	-2.30956100	-2.81042500	0.13117100
O	-1.54374300	-4.13340500	1.42120500
O	-2.72484500	-1.29653800	-1.26518700
O	-3.43107300	-4.38305700	-0.85197200
S	-2.34846300	-4.77967500	-1.84700300
S	-2.44351500	-0.17963000	-2.22787500
S	-0.24233700	-4.77270000	1.94252400
O	-3.50196900	0.08396500	-3.18156600
O	-1.91576400	1.03310100	-1.50101000
O	-0.47244400	-6.14564700	2.37299600
O	0.90627700	-4.47503100	1.08454200
O	-1.99505800	-6.18161100	-1.93524000
O	-1.24493000	-3.77885700	-1.58070200
C	0.02011900	-3.78370200	3.52272200
C	-3.07463500	-4.30748000	-3.50715600
C	-0.91982200	-0.71117300	-3.19290100
F	0.73147100	-4.51756700	4.37058900
F	0.68596300	-2.66040800	3.26186200
F	-1.14735900	-3.47710900	4.07797300
F	-3.58750300	-3.08642000	-3.44606200
F	-4.02731000	-5.17489000	-3.81255600

F	-2.11815400	-4.34938200	-4.42240100
F	0.02447200	-1.12763400	-2.36648700
F	-1.26019400	-1.69369800	-4.01439000
F	-0.49007800	0.32605200	-3.88773700
Sc	-1.96212400	2.31080800	0.10956200
O	-2.80151500	0.69170100	1.13501100
O	-0.80721500	3.60149600	-0.89025900
O	-3.51419900	3.51132000	0.35918700
S	-4.70022800	3.60419500	1.36402100
S	0.51644800	3.76148100	-1.66819200
S	-3.49756000	-0.27356200	2.04869300
O	1.67937000	3.67865000	-0.78127000
O	0.49716700	3.03544000	-2.93242400
O	-4.88762300	-0.01331300	2.35372300
O	-3.21848200	-1.69028100	1.59670900
O	-5.24419600	4.95462700	1.39634800
O	-4.38635200	2.90968600	2.60932300
C	-2.49502700	-0.16260900	3.62710700
C	-5.96708500	2.56096400	0.44140400
C	0.38358700	5.56800700	-2.11600600
F	-3.00788500	-0.99698300	4.51507300
F	-1.23700700	-0.49928900	3.36784100
F	-2.54118400	1.07618100	4.08164000
F	-5.38722700	1.49976500	-0.11889100
F	-6.52787200	3.29499700	-0.51227800
F	-6.89755900	2.15576300	1.29321600
F	-0.69121900	5.77965500	-2.86622700
F	0.30793800	6.31104600	-1.01708500
F	1.47177500	5.91566000	-2.80285700
C	5.15214500	0.63009200	-4.69218500
C	5.88494600	1.97612400	-4.65118000
C	4.91085800	3.13578600	-4.41343900
C	4.08622200	2.91909000	-3.13881800
C	3.37187100	1.56701800	-3.15344500
C	4.33641000	0.40993600	-3.41212900
H	5.45860500	4.09164700	-4.35439800
H	6.62862100	1.95682000	-3.83246600
H	6.45084700	2.13422400	-5.58460400
H	4.47440600	0.61126800	-5.56505700
H	5.86999900	-0.19586700	-4.83400500
H	4.76311700	2.95970200	-2.26886300
H	3.35525500	3.72878300	-2.99996100
H	2.82962400	1.41738300	-2.20005300
H	2.58164100	1.57595200	-3.92329700
H	5.04144000	0.32976900	-2.57076100
H	3.78984600	-0.54698400	-3.45513700
H	4.22709900	3.21746200	-5.27803900

^{3}TS4

Os	-1.85143000	-1.99982800	-0.38272800
C	-2.28025600	-3.94332800	-0.04846400
N	-2.48094100	-5.06584600	0.15333800
C	2.64758900	-3.08043700	-0.02750200
C	3.31247700	-1.87012500	-0.06117000

C	2.53538300	-0.68380500	-0.17644900
C	1.13366400	-0.81101200	-0.30041900
C	1.26001400	-3.12068300	-0.14474900
C	3.10589500	0.62509500	-0.14888900
C	0.31923100	0.37283900	-0.44421200
C	0.92159100	1.65082600	-0.41287000
C	2.33860000	1.73624700	-0.26234400
C	0.08515200	2.79305800	-0.55624000
C	-1.26012400	2.58690200	-0.79488400
C	-1.77719700	1.29418600	-0.80944800
H	4.17372800	0.72277600	-0.03055500
H	3.19660300	-4.00655800	0.05978200
H	0.72818000	-4.06188400	-0.13230900
H	2.80246200	2.71017600	-0.25205800
H	-1.93094600	3.42420800	-0.91880600
H	-2.83392300	1.11765100	-0.95423300
N	0.53202900	-2.02744700	-0.29126600
N	-1.01776400	0.23190800	-0.61375900
O	-1.83304800	-2.11451600	-2.12699600
O	-1.65528400	-1.63088100	1.51661300
C	-3.82006200	-1.62811000	-0.26814900
N	-4.94160700	-1.35366100	-0.17001400
C	4.78837300	-1.84551600	0.02161800
C	5.43598500	-2.49031300	1.07808000
C	5.55499900	-1.22518500	-0.96874100
C	6.82266600	-2.49984000	1.15158100
H	4.84944400	-2.96982900	1.85083800
C	6.94119400	-1.24766600	-0.89979200
H	5.06493900	-0.74509400	-1.80528500
C	7.57830700	-1.87965200	0.16286800
H	7.31218000	-2.99230600	1.98136000
H	7.52389100	-0.77423700	-1.67889800
H	8.65882700	-1.89135400	0.21799600
C	0.59663600	4.17313000	-0.43320000
C	1.31136400	4.57199000	0.69989200
C	0.31244000	5.11649400	-1.42357700
C	1.73389200	5.88665100	0.83685200
H	1.51364200	3.85747800	1.48589000
C	0.74876100	6.42784400	-1.29095100
H	-0.23756800	4.81565600	-2.30557300
C	1.45863700	6.81649500	-0.16015100
H	2.27447300	6.18559600	1.72510900
H	0.53316200	7.14606400	-2.07087100
H	1.79323700	7.84001200	-0.05470600
H	-2.42694900	-1.53707700	2.09281900
C	-0.90089800	1.25449100	3.11286600
C	-0.77429000	2.72276900	3.57568000
C	-1.79552900	3.61417800	2.86875100
C	-3.22467300	3.10554500	3.06450600
C	-3.36711300	1.63416300	2.60991600
C	-2.30856500	0.79040300	3.22085000
H	-1.71160500	4.64043200	3.23482000
H	-0.93672900	2.77193800	4.65670200
H	0.24135900	3.08054700	3.38991600

H	-0.22548500	0.61136900	3.67779100
H	-3.49253000	3.17641400	4.12308400
H	-3.93188700	3.72916200	2.51218500
H	-4.36313800	1.25567900	2.84162400
H	-3.27024900	1.61497100	1.51577900
H	-2.57012300	-0.00565100	3.90582700
H	-1.56675900	3.64628600	1.79961300
H	-0.57764300	1.20652900	2.06573700
